# RAVEN: Robust, generalizable, multi-resolution structural MRI upsampling using autoencoders

**DOI:** 10.1162/IMAG.a.1326

**Published:** 2026-08-04

**Authors:** Walter Adame-Gonzalez, Roqaie Moqadam, Yashar Zeighami, Mahsa Dadar

**Affiliations:** Integrated Program in Neuroscience, McGill University, Montreal, Quebec, Canada; Douglas Mental Health University Institute, Verdun, Quebec, Canada; Department of Medicine, Université de Montréal, , Montreal, Quebec, Canada; Department of Psychiatry, McGill University, Montreal, Quebec, Canada

**Keywords:** single image super-resolution, magnetic resonance imaging, deep learning, contrast agnostic, resolution agnostic

## Abstract

Due to their high inter-tissue contrast, Magnetic resonance images (MRIs) can reflect neuroanatomical changes related to healthy aging and pathological processes. However, standard brain MRI acquisition resolutions hinder the ability to measure the more subtle changes that occur in early disease stages. Increasing the resolution during acquisition poses multiple challenges, including increased noise, higher acquisition times and cost, and discomfort of the scanned individual. In this work, we propose a robust, generalizable single-image super-resolution network for brain MRIs named Resolution Augmentation with Variational auto-Encoder Networks (RAVEN) with generative adversarial networks (GANs). We show RAVEN is capable of upsampling in-vivo and ex-vivo MRIs of diverse modalities (e.g. T1-weighted, T2-weighted, and T2*) and varying field strengths (3T to 7T) to target voxel sizes as small as 0.5 mm isotropic using arbitrary upsampling factors. RAVEN achieved state-of-the-art performance against deep learning and non-deep learning methods, best preserving true anatomical information. We have also made RAVEN open access, with the source code as well as training and evaluation scripts available and ready to use at: https://github.com/waadgo/raven.

## 1 Introduction

Magnetic Resonance Imaging (MRI) is a non-invasive technique widely used in clinical and research protocols for studying human brains in-vivo. In research settings, MRI scans are commonly acquired at voxel sizes of approximately 1 mm isotropic and analyzed with automatic image processing pipelines (Dadar et al., 2025; Iglesias et al., 2021; Lüsebrink et al., 2018). However, early detection of fine brain atrophy patterns in neurodegenerative disorders (e.g., Alzheimer’s disease) requires accurate morphometry of small brain regions for which standard resolutions are suboptimal (Shafiee et al., 2024).

Acquisition voxel size is a hindering factor when performing brain morphometry, since it limits the minimum size of brain regions that would be measurable on an MRI. Therefore, it is desirable to acquire MRIs with the highest resolution possible. However, increasing acquisition resolution substantially increases noise level as well as time, noise, discomfort of scanned individuals, and makes the scan more prone to movement artifacts (Zhu et al., 2009). Therefore, in clinical studies and practical applications, it is uncommon to use high-resolution full brain MRI acquisitions. Furthermore, numerous legacy datasets have been scanned using standard-resolution sequences, but contain a great amount of non-imaging biomarker information. As an example. the the Alzheimer’s Disease Neuroimaging Initiative (ADNI) (Mueller et al., 2005), one of the largest databases of individuals on the continuum of Alzheimer’s Disease containing longitudinal demographic, genetic, and clinical information, as well as positron-emission tomography (PET) scans, provides longitudinal MRI data acquired at ~1 mm isotropic voxel sizes.

Single-Image Super-Resolution (SISR) is a technique to artificially increase the resolution of images after their acquisition, generating a high-resolution (HR) image from a single low-resolution (LR) input. SISR has gained popularity in naturalistic image processing (Fischer et al., 2024; Jo et al., 2020; Rombach et al., 2022; Shi et al., 2016; Umer et al., 2020; Umer & Micheloni, 2020; X. Wang et al., 2018; Zeng et al., 2017; K. Zhang et al., 2021), as well as in medical image processing (Avants et al., 2023; Y. Chen et al., 2018; Iglesias et al., 2021; Lin et al., 2023; J. Wang et al., 2023; Zhao et al., 2025). The latter implies potential valuable gains in accuracy for medical diagnostic and prognostic purposes, and could even make it possible to measure anatomical structures that would be too small to capture in the LR regime (Shafiee et al., 2024). SISR is an ill-posed problem since a single LR sample can be upsampled into many different HR images. Therefore, caution should be taken when utilizing image-reconstruction techniques in the medical field, and thorough validations should be performed before publicly releasing an SISR method.

Several strategies have been proposed for MRI SISR in the literature. Early non-learning approaches include Non-local Means Upsampling (NLMUPSAMPLE) (Manjón et al., 2010), a contrast- and resolution-agnostic self-similarity method that iteratively refines a 3D estimate while enforcing consistency (i.e., voxel-level similarity) between the input LR image and a downsampled version of the provisional HR image. More recently, deep learning (DL) has achieved state-of-the-art performance owing to its capacity to model non-linear mappings. For example, Y. Chen et al. (2018) proposed a 3D densely connected convolutional neural network (CNN) for SISR of T1w MRIs using an upsampling factor of 2 × 2 × 2, demonstrating improvements over 2D CNN counterparts and interpolation. Another relevant study (Lyu et al., 2018) introduced a Generative Adversarial Network (GAN) trained on 2D patches for upsampling single-modality T2-weighted (T2w) MRIs, likewise outperforming interpolation. Additionally, Avants et al. (2023) proposed ANTsPyNet for MRI SISR based on the previous work of Tustison et al. (2021) based on a 3D Deep Back-Projection Networks (DBPN) (Haris et al., 2018) for upsampling of T1-weighted (T1w) MRIs. More recently, J. Wang et al. (2023) proposed brain MRI SISR using latent diffusion models, and later Zhao et al. (2025) proposed partial diffusion models, in which a compressed representation of the low-resolution input is degraded with normal gaussian noise and then gradually denoised to match the latent representation of a high-resolution MRI. While most other works for MRI SISR aimed for through-plane upsampling of 2D MRI samples (Lin et al., 2023; Zhao et al., 2025), or propose independent weights for a finite set of upsampling factors (Avants et al., 2023; Haris et al., 2018; J. Wang et al., 2023), Wu et al. (2023) proposed ArSSR, an arbitrary scale super-resolution method for T1w brain MRIs capable of handling arbitrary upsampling factors for all three anatomical axes using an encoder-decoder approach.

Despite these advances, these prior works mainly focus on in-vivo T1w and T2w contrasts, and are limited to specific target resolutions or upsampling factors, limiting their flexibility for use in applications with target upsampling factors that are outside of their predetermined options in the case of the former or where input images have different ranges of resolutions (e.g. in multi-center/multi-cohort applications) and the goal is to upsample all images to a consistent target resolution in the case of the latter. The present work aimed to address these gaps. To achieve this, we trained a single network that is capable of processing multiple MRI contrasts, magnetic field strengths, in-vivo and ex-vivo images of in situ brains as well as ex situ hemispheres, enabling generalizability of the network to multiple inter-tissue contrasts. Additionally, we trained and validated our network using multiple input and target voxel sizes, modeling different degradation functions, and further validating on out-of-distribution data, ensuring the robustness and generalizability of its performance. Moreover, we evaluated segmentation performance on the upsampled images as a downstream task after super-resolution, demonstrating the ability of our method to reconstruct true anatomical details. Finally, to validate the biological plausibility of the reconstructed high-resolution data, we used features derived from deformation-based morphometry analysis obtained from upsampled MRIs from an age- and sex-matched unseen cohort from the ADNI dataset to study group differences between cognitively unimpaired and cognitively impaired groups. We observed that the features obtained from RAVEN-upsampled MRIs consistently yielded statistically significant differences between cognitively unimpaired and three cognitively impaired groups of differing severities, suggesting that RAVEN preserves true structural characteristics of the MRIs.

## 2 Methods

### 2.1 Datasets

To train deep neural networks for brain MRI super-resolution that generalize across varying tissue contrasts, voxel sizes, and acquisition parameters (i.e., different modalities), it is essential to use datasets that are as diverse as possible. As reported in Table 1, we used an aggregate training dataset that includes native images acquired at isotropic voxel sizes ranging from 0.4 mm to 1 mm from in-vivo T1w, T2w, T2*, and T1-maps, and ex-vivo T1w and T2w head and hemisphere scans acquired at multiple resolutions (see Supplementary Materials-Data Description section for a detailed description of the used datasets).

**Table 1.** Datasets used for training, validating, and testing RAVEN.

Dataset	Split	Modality	Field strength	N	Voxel size [mm]	Individuals	Ages
AHEAD (in-vivo) (Alkemade et al., 2020)	Train	T1 map	7T	66	0.7 × 0.7 × 0.7	66	18-80
		T1w	7T	66	0.7 × 0.7 × 0.7		
	Validation	T1 map	7T	19	0.7 × 0.7 × 0.7	19	
		T1w	7T	19	0.7 × 0.7 × 0.7		
	Test	T1 map	7T	10	0.7 × 0.7 × 0.7	10	
		T1w	7T	10	0.7 × 0.7 × 0.7		
AOMICID1000 (in-vivo) (Snoek et al., 2021)	Train	T1w	3T	648	1.0 × 1.0 × 1.0	648	19-26
	Validation	T1w	3T	185	1.0 × 1.0 × 1.0	185	
	Test	T1w	3T	94	1.0 × 1.0 × 1.0	94	
AOMICPIOP1 (in-vivo) (Snoek et al., 2021)	Train	T1w	3T	151	1.0 × 1.0 × 1.0	151	19-26
	Validation	T1w	3T	43	1.0 × 1.0 × 1.0	43	
	Test	T1w	3T	22	1.0 × 1.0 × 1.0	22	
AOMICPIOP2 (in-vivo) (Snoek et al., 2021)	Train	T1w	3T	158	1.0 × 1.0 × 1.0	158	19-26
	Validation	T1w	3T	45	1.0 × 1.0 × 1.0	45	
	Test	T1w	3T	22	1.0 × 1.0 × 1.0	22	
CHEN (in-vivo) (X. Chen et al., 2023)	Train	T1w	3T	7	0.8 × 0.8 × 0.8	7	25-41
		T1w	7T	7	0.65 × 0.65 × 0.65		
	Validation	T1w	3T	2	0.8 × 0.8 × 0.8	2	
		T1w	7T	2	0.65 × 0.65 × 0.65		
	Test	T1w	3T	1	0.8 × 0.8 × 0.8	1	
		T1w	7T	1	0.65 × 0.65 × 0.65		
DBCBB** (post-mortem) (Dadar et al., 2024)	Train	T1w	3T	152 (3*)	varying	103	50-105
		T2w	3T	152 (3*)	varying		
	Validation	T1w	3T	43 (2*)	varying	34	
		T2w	3T	43 (2*)	varying		
	Test	T1w	3T	21	varying	21	
		T2w	3T	21	varying		
DBCBB** (post-mortem) (Dadar et al., 2024)	Test	T1w	3T	21 (2*)	0.7 × 0.7 × 0.7	21	56-96
		T1w	3T	21 (2*)	1.4 × 1.4 × 1.4		
		T1w	3T	21 (2*)	2.1 × 2.1 × 2.1		
		T2w	3T	21 (2*)	0.64 × 0.64 × 0.64		
		T2w	3T	21 (2*)	1.68 × 1.68 × 1.68		
HBA (in-vivo) (Schira et al., 2023)	Train	T1w	7T	1	0.5 × 0.5 × 0.5	1	30, 45
		T2w	7T	1	0.4 × 0.4 × 0.4		
	Validation	T1w	7T	0	0.5 × 0.5 × 0.5	0	
		T2w	7T	0	0.4 × 0.4 × 0.4		
	Test	T1w	7T	1	0.5 × 0.5 × 0.5	1	
		T2w	7T	1	0.4 × 0.4 × 0.4		
HCP (in-vivo) (Van Essen et al., 2012)	Train	T1w	3T	773	0.7 × 0.7 × 0.7	773	22, 35
		T2w	3T	771	0.7 × 0.7 × 0.7		
	Validation	T1w	3T	119	0.7 × 0.7 × 0.7	119	
		T2w	3T	119	0.7 × 0.7 × 0.7		
	Test	T1w	3T	110	0.7 × 0.7 × 0.7	110	
		T2w	3T	110	0.7 × 0.7 × 0.7		
OSCBS (in-vivo) (Tardif et al., 2016)	Train	T1w	7T	18	0.5 × 0.5 × 0.5	18	26-27
		T2*	7T	6	0.5 × 0.5 × 0.5		
	Validation	T1w	7T	5	0.5 × 0.5 × 0.5	5	
		T2*	7T	3	0.5 × 0.5 × 0.5		
	Test	T1w	7T	3	0.5 × 0.5 × 0.5	3	
		T2*	7T	0	0.5 × 0.5 × 0.5		
LUSEBRINK (in-vivo) (Lüsebrink et al., 2018)	Train	T1w	7T	0	1.0 × 1.0 × 1.0	0	35
		T1w	7T	0	0.5 × 0.5 × 0.5		
		T1w	7T	0	0.25 × 0.25 × 0.25		
	Validation	T1w	7T	0	1.0 × 1.0 × 1.0	0	
		T1w	7T	0	0.5 × 0.5 × 0.5		
		T1w	7T	0	0.25 × 0.25 × 0.25		
	Test	T1w	7T	1	1.0 × 1.0 × 1.0	1	
		T1w	7T	1	0.5 × 0.5 × 0.5		
		T1w	7T	1	0.25 × 0.25 × 0.25		

* Indicates full-head post-mortem specimens.

** Full details on per-specimen pathologies can be found in Supplementary Table S1.

### 2.2 Data preprocessing

Preprocessing consisted of denoising the images with FONDUE (Adame-Gonzalez et al., 2024), a DL-based method capable of denoising multiple input modalities and resolutions, followed by reorientation to Right-Anterior-Superior (RAS) and robust intensity rescaling between 0 and 255 (clamping the intensities beyond the 99.5 percentiles). The axes were then randomly swapped (changing the effective brain orientation) and a foreground mask was created through an intensity threshold to separate background (empty) voxels from the foreground (tissue) consistently across different input modalities. Non-overlapping patches of shape 192 × 192 × 32 (center cropped during training to 160 × 160 × 32) were used for memory efficiency. The patch was centered in the high-resolution plane, with the sliding window for extracting the patches moving along the third axis. As expected, some patches contained no foreground information. Since the goal was to train a model that upsamples the foreground but still has some training on how to handle background patches, 50% of the patches containing less than 20% voxels with foreground information were randomly dropped. Finally, the individual patches were normalized to the intensity range of [-1, 1] using min-max normalization (linear rescaling).

### 2.3 Architecture

**Fig. 1.**
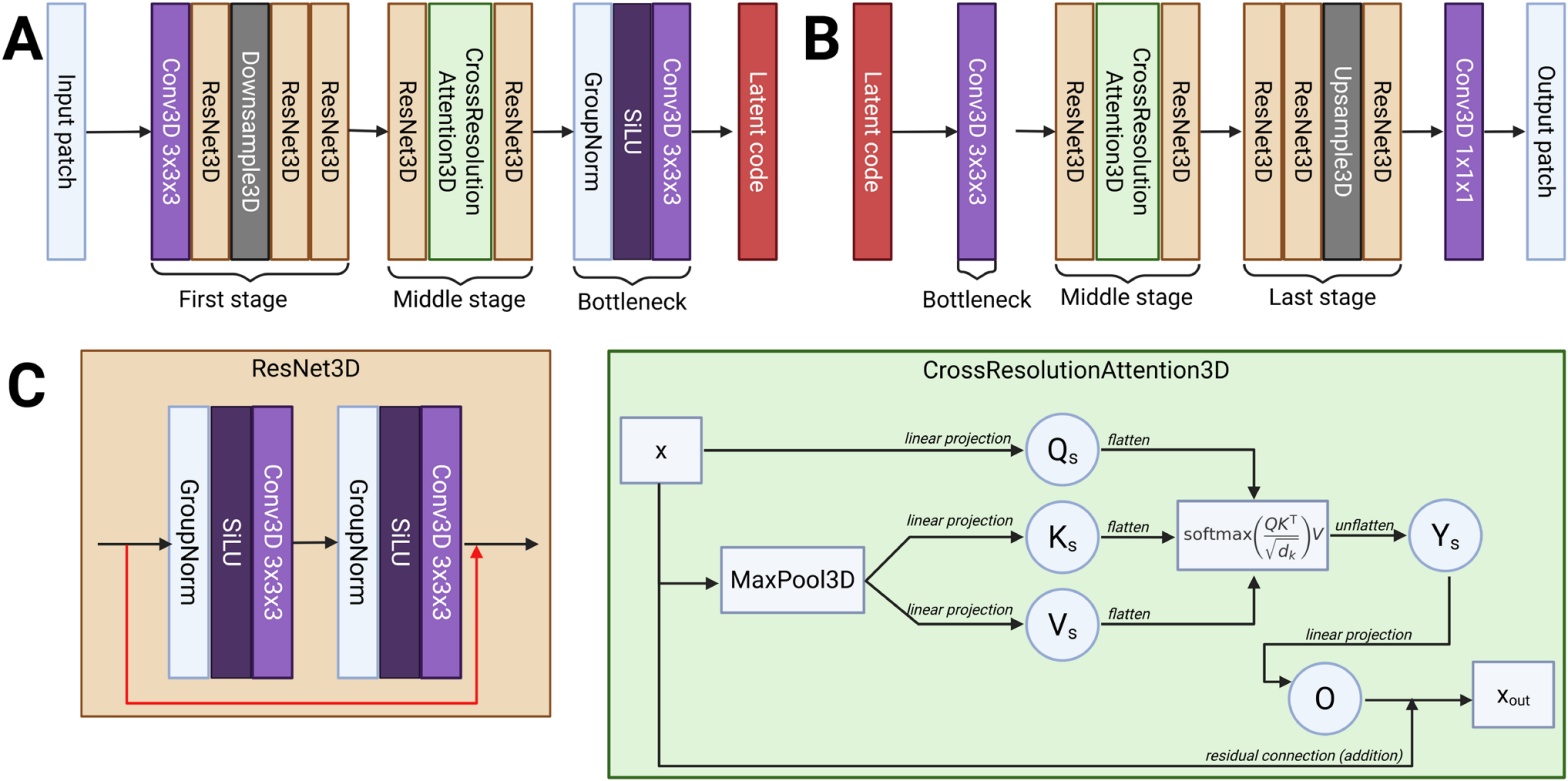
RAVEN architecture: Panel (A) depicts the encoder, receiving the input patch of dimensions (D)×(H)×(W) with three main stages and as output a 3D latent code of shape (D÷2)×(H÷2)×(W÷2). Panel (B) shows the decoder receiving as input the latent code, and generating an output patch with dimensions (D)×(H)×(W). Panel (C) depicts the internal architecture of ResNet3D and the CrossResolutionAttention3D module.

We trained a Variational AutoEncoder Generative Adversarial Network (VAE-GAN) with small KL-regularization adapted from the method by Rombach et al. (2022) named Resolution Augmentation with Variational auto-Encoder Networks with GANs (RAVEN). Figure 1 shows the architecture of RAVEN.

#### 2.3.1 Encoder

**The generator encoder ${\text{G}}_{\text{E}}$** consists of three stages (Fig. 1-A):

***Stage 1:*** Conv3D with 3×3×3 kernel, stride of 1 and padding of 1, followed by three consecutive ResNet3D blocks composed of two consecutive GroupNorm, SiLU non-linearity, and Conv3D with 3×3×3 kernel, stride of 1 and padding of 1 and the resulting tensor is then added to the input (residual operation). After the first ResNet3D block, there is a Downsample3D block consisting of a Conv3D with kernel size of 3×3×3, stride of 2 and padding of 0. The three ResNet3D blocks in this stage gradually increase the number of filters (64, 128, and 256).

***Stage 2:*** The second stage of $G_{E}$ is composed of a ResNet3D block (two stacked layers, each composed of GroupNorm, SiLU, and Conv3D operations, followed by a residual connection, see Fig. 1, panel C) followed by a CrossResolutionAttention3D layer – in which the feature map attends to a downsampled version of itself – followed by another ResNet3D block. The proposed CrossResolutionAttention3D creates attention maps between the raw input and its respective downsampled version by a factor of 2 using MaxPool3d. We base our approach on previous work by us and other groups (Adame-Gonzalez et al., 2024; Henschel et al., 2022) in which feature maps were downsampled using MaxPooling operations showing efficient and robust results, proposing an efficient modified self-attention mechanism that enables global patch attention operation using 8x less memory and allowing for a faster forward step. More specifically, we define an input feature map $x\in {ℜ}^{BS\times C\times \left(\frac{H}{2}\right)\times \left(\frac{W}{2}\right)\times \left(\frac{D}{2}\right)}$ which is normalized using group normalization as $\hat{x}=\;GN\left(x\right)$. The Queries tensor is computed through a 1×1×1 convolution as $\;Q_{S}=\;W_{q}*\hat{x}\in {ℜ}^{BS\times C\times \left(\frac{H}{2}\right)\times \left(\frac{W}{2}\right)\times \left(\frac{D}{2}\right)}$. Keys and Values are computed from the downsampled $\hat{x}$ tensor ${\hat{x}}_{↓}\in {ℜ}^{BS\times C\times \left(\frac{H}{4}\right)\times \left(\frac{W}{4}\right)\times \left(\frac{D}{4}\right)}$ defined as ${\hat{x}}_{↓}\;=Pool\left(\hat{x}\right)$, where Pool(⋅) represents a MaxPool3d using a pooling factor of 2. Then, the final Keys and Values tensors are computed with $K_{S}=\;W_{k}*$ ${\hat{x}}_{↓}$ and $V_{S}=\;W_{v}*\;{\hat{x}}_{↓}$ respectively. Finally, $Q_{S}$, $W_{S}$, and $V_{S}$ are flattened to the shapes: $Q\in {ℜ}^{BS\times C\times N_{q}}$, $K\in {ℜ}^{BS\times C\times N_{k}}$, and $V\in {ℜ}^{BS\times C\times N_{v}}$, respectively, with $N_{q}\;=\left(\frac{H}{2}\right)\left(\frac{W}{2}\right)\left(\frac{D}{2}\right)=\frac{HWD}{8}$ and $N_{k}\;=N_{v}\;=\left(\frac{H}{4}\right)\left(\frac{W}{4}\right)\left(\frac{D}{4}\right)=\frac{HWD}{64}$ The final Attention weight matrix A is computed with $A=softmax\left(\frac{QK^{⊤}}{\sqrt{C}}\right)$ $\in {ℜ}^{BS\times N_{q}\times N_{k}}$ and the attention output Y  being $Y=AV\in {ℜ}^{BS\times N_{q}\times C}$ which gets “unflattened” as $Y_{s}\;=Unflatten\left(Y\right)\in {ℜ}^{BS\times C\times \left(\frac{H}{2}\right)\times \left(\frac{W}{2}\right)\times \left(\frac{D}{2}\right)}$. $Y_{s}$ is then projected with a 1×1×1 convolution with $O=W_{o}*Y_{s}$ and is added to the input feature map as a residual connection to produce the final output feature map $x_{out}\;=x\;+\;O$ .

***Stage 3:*** a GroupNorm followed by SiLU non-linearity and a Conv3D operation with 3×3×3 kernel, stride of 1 and padding of 1.

#### 2.3.2 Decoder

**The generator decoder ${\text{G}}_{\text{D}}$** is similar in architecture to the encoder in reverse order (Fig. 1-B):

***Stage 3:*** an initial Conv3D layer with stride of 1 and padding of 1 with a kernel size of 3×3×3 and 256 channels.

***Stage 2:*** two ResNet3D blocks with a CrossResolutionAttention3D block between them, with ResNet3D blocks using 256 channels each.

***Stage 1:*** Three ResNet3D blocks of decreasing channel count, with 256, 128, and 64 channels for the first, second, and third ResNet3D blocks, respectively. Between the second and third ResNet3D blocks, we apply an Upsample3D block that upsamples the 3D feature maps using nearest neighbor interpolation with an upsampling factor of 2.0 for all three axes to bring the dimensionality of the feature maps to match the output dimensions, followed by a Conv3D of 128 channels, kernel size of 3×3×3, padding and stride of 1. Finally, to generate the output image, we utilize a Conv3D unit with 1 output channel, kernel size of 3×3×3, padding and stride of 1.

#### 2.3.3 Discriminator

**The discriminator D** is a patch-based discriminator (Demir & Unal, 2018; Rombach et al., 2022) consisting of 4 stages:

***Stage 1:*** initial Conv3D with 64 channels, kernel size of 4 × 4 × 4, stride of 2 and padding of 1 followed by a LeakyReLU non-linearity.

***Stage 2:*** a stack of 3 sequentially placed Conv3d with kernel size of 4 × 4 × 4, stride of 2 and padding of 1, followed by a BatchNorm3D layer and a LeakyReLU layer. In this stage, the first, second, and third Conv3d and BatchNorm3D operations have 128, 256, and 512 channels, respectively.

***Stage 3:*** A Conv3d with 512 filters, kernel size of 4 × 4 × 4, stride of 1 and padding of 1 followed by a BatchNorm3D and a LeakyReLU non-linearity.

***Stage 4:*** A single Conv3d layer with 512 input channels and 1 output channel using a kernel size of 4, with stride and padding of 1.

### 2.4 Training procedure

#### 2.4.1 Generation of LR-HR image pair

**Fig. 2.**
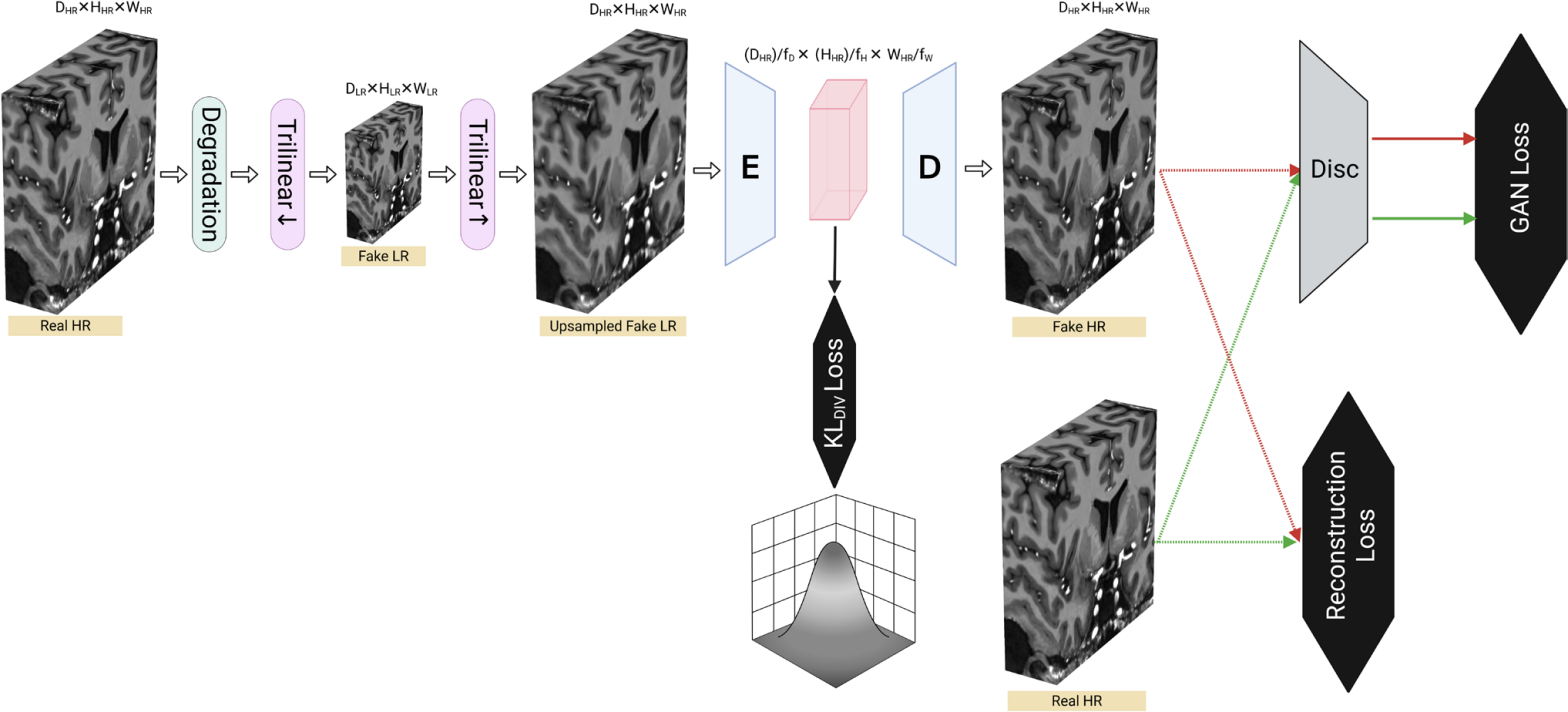
Training procedure for RAVEN: A surrogate of low-resolution image patch (Fake LR) is generated by degrading and downsampling (through trilinear interpolation) an input non-degraded image patch (Real HR). Then, the input to the encoder (E) is generated by resampling the Fake LR image to the target space using trilinear interpolation (Upsampled Fake LR). The latent space is a compressed representation of the input patch and is regularized using KL Divergence. The generated Fake HR and the Real HR patches get parsed to the discriminator network (Disc) to compute the discriminator loss and the GAN loss, as well as the Reconstruction loss.

Given a reference ground-truth 3D volume by the 5-dimensional tensor $x\in R^{BS\times C\times H\times W\times D}$ , where BS indicates the batch size, C indicates the number of channels (1 in our case), and H, W, and D the indicate dimensions of our 3D patch respectively (160, 160, 32), we define a set of random degradations by first defining our downsampling factors $df\;=\left[df_{H},\;df_{W},\;df_{D}\right]$, each of the three sampled from [1, 2, 3] with probabilities of [0.1, 0.45, 0.45] to ensure that downsampling factor of 1, i.e., no downsampling at all, is included in the training but with a small frequency compared to the other two downsampling factors. $df_{H}$, $df_{W}$, and $df_{D}$ will be sampled independently during runtime. Then, an adaptive gaussian blurring function $\varphi_{blur}$ will be applied by first defining the σ values for each axis in our image, i.e., defining $\sigma =\left[\sigma_{H},\;\sigma_{W},\;\sigma_{D}\right]$ with $\sigma_{H}=\frac{df_{H}}{2}\left[1+U\left(-0.5,\;0.5\right)\right]$*, $\sigma_{W}=\frac{df_{W}}{2}\left[1+U\left(-0.5,\;0.5\right)\right]\;$ ,* and *$\sigma_{D}=\frac{df_{D}}{2}\left[1+U\left(-0.5,\;0.5\right)\right]\;$ ,* where U(−0.5, 0.5) represents a random number drawn from a uniform distribution and was included as a blurring augmentation step during training. We then define the kernel size k for each axis as $k=\left[k_{H},\;k_{W},\;k_{D}\right]$, $k_{H}\;=\left(2ceil\left(2\sigma_{H}+1\right)\right)\;$ *, $k_{W}\;=\left(2ceil\left(2\sigma_{W}+1\right)\right)\;$ , $k_{D}\;=\left(2ceil\left(2\sigma_{D}+1\right)\right)\;$* and apply the blurring independently to each axis using Conv3D from PyTorch. The mentioned blurring will be applied randomly to 50% of the training batches. Our low-resolution degraded image $x_{LR}\in R^{BS\times C\times \frac{H}{df_{H}}\times \frac{W}{df_{W}}\times \frac{D}{df_{D}}}$ will be generated with $x_{LR}\;=\;\varphi_{↓,df}\varphi_{blur,df}\left(x\right)\;$ *w*here $\varphi_{↓,df}$ is a trilinear downsampling interpolation with downsampling factors 1​/​df . Finally, since our network design requires the input image to be in the same space (resolution) as the target image, we generate the final input to our network $x_{LR\_fake}$ as $x_{LR\_fake}=\;\varphi_{↑,df}\left(x_{LR}\right)$ with $x_{LR\_fake}\in R^{BS\times C\times H\times W\times D}$ , where $\varphi_{↑,df}$ is a trilinear upsampling interpolation with factors df . The task of the network $G_{\theta }$ is to learn the mapping between $x_{LR\_fake}$ and $x_{HR\_fake}$ , which is an approximation of x (Fig. 2).

#### 2.4.2 Loss functions

D is optimized by minimizing $L_{D}$ defined as

$$L_{D}\;=E_{x}\left[max\left(0,\;1-D_{\psi }\left(x\right)\right)\right]+\;E_{x}\left[max\left(0,\;1+D_{\psi }\left(x_{HR\_fake}\right)\right)\right]\;$$

with D being the discriminator network parametrized by ψ. The generator G is optimized by minimizing the loss function

$$L_{G}\;:=L_{NLL}+\omega_{KL}L_{KL}+\omega_{GAN}L_{G}^{GAN}\;$$

where $L_{NLL}$ is defined as:

$$L_{NLL}\;=\;\frac{L_{rec}}{\sigma^{2}}+log\sigma^{2}\;$$

$\sigma^{2}$ is a learnable parameter and $L_{rec}$ is defined as:

$$L_{rec}\;=2.0L_{LPIPS}\;+0.2L_{MAE}\;$$

Both $L_{LPIPS}$ and $L_{MAE}$ are defined as $LPIPS\left(x_{HR\_fake},x\right)$ and $MAE\left(x_{HR\_fake},x\right)$. $L_{KL}$ is the KL divergence between z defined as $z=G_{E}\left(x_{LR\_fake}\right)$, and a normal distribution N(0, I) defined as

$$L_{KL}\;=\;E_{x\sim p_{data}}\left[D_{KL}\left(q_{\phi }\left(z|x\right)\;||N\left(0,\;I\right)\right)\right]\;$$

where q is the probability density mass of $G_{E}$ parametrized by ϕ. $L_{G}^{GAN}$ is defined as

$$L_{G}^{GAN}\;=-E_{x}\left[D_{\psi }\left(x_{HR\_fake}\right)\right]\;$$

$\omega_{KL}$ is set to 1e-8, and $\omega_{GAN}$ is an adaptive function set to (Esser et al., 2021),

$$\omega_{GAN}=0.1\frac{\nabla G_{L}\left[L_{rec}\right]}{\nabla G_{L}\left[L_{G}^{GAN}\right]+\;\delta }\;$$

with $\nabla G_{L}\left[L_{rec}\right]$ denotes the L2 norm of the gradients of $L_{rec}$ with respect to the last layer of $G_{D}$, $\nabla G_{L}\left[L_{G}^{GAN}\right]$ is the L2 norm of the gradients of $L_{G}^{GAN}$ with respect to the last layer of $G_{D}$, and δ is a stability constraint set to 10e-6.

#### 2.4.3 Optimization

We trained RAVEN for 30 epochs, reaching convergence at epoch 22 (step ~250k) using a batch size of 1, accumulating gradients for 2 iterations for G and 2 for D. $\omega_{KL}$ was set to 1e-8, and G was trained decoupled from D (G warmup) during one epoch. The learning rate was set to constant 9e-4 for the generator and 1.8e-3 for D, updating the discriminator 4 times per 1 update of the generator. Mixed-precision (bf16-mixed) was the datatype used for data efficiency, and the optimizer was set to AdamW with β1 = 0.5 and β2 = 0.9.

#### 2.4.4 Generating LR-HR image pairs

As stated above, few datasets contain images of the same participant/specimen acquired using the same scanner, same acquisition parameters, with the only difference being using two different resolutions (i.e., voxel sizes; either in-plane or slice-thickness). As such, it is necessary to generate LR surrogates that can closely resemble those acquired natively at LR. To achieve this, one could either apply k-space cropping (Forigua et al., 2022; Wu et al., 2023) (effectively removing the parts of the Fourier space corresponding to high-frequencies) or downsampling in the image domain using, e.g., interpolation after having blurred the image to prevent aliasing artifacts as described above (K. Zhang et al., 2021).

**Fig. 3.**
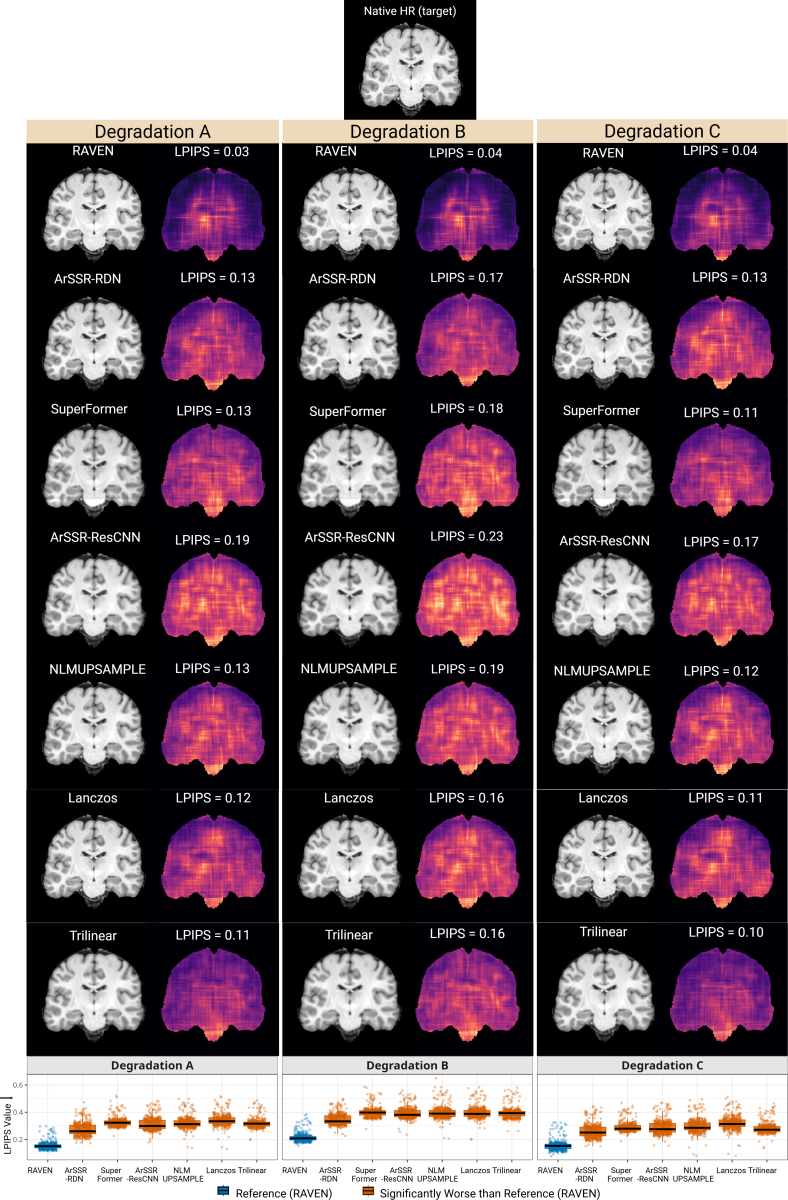
Top: Examples of coronal slices from a T1w image (the HCP dataset) downsampled by a factor of 2x isotropic using degradation schemes A, B, and C and then upsampled with different methods (left) as well as performance as measured by LPIPS (lower is better). Bottom: Boxplots reflecting the distribution of LPIPS values obtained for each method across the test dataset. Boxplot colors indicate significance of paired t-test vs. RAVEN after FDR correction. Each datapoint represents the average metric value of an individual across the different tested degradations (A, B, and C) and upsampling factors (×2 and ×3). The color represents the significance after performing paired t-tests on RAVEN vs each of the methods tested separately.

While the majority of proposed SISR methods report test results using the same degradation functions as those used for training, we tested the robustness of the trained network with the proposed degradation function $\varphi_{↓,df}\varphi_{blur,df}$ to different degradation regimes such as interpolation after different intensities of gaussian blurring and k-space cropping. We opted to do so since no consensus exists on which degradation regime is best (i.e., most realistic) to generate low-resolution surrogates of an MRI for SISR tasks. Therefore, we assessed three most widely used test degradation schemes, two using varying parameters of $\varphi_{↓,df}\varphi_{blur,df}$ , and one using k-space cropping (Fig. 3):

- *Degradation A*: using $\varphi_{↓,df}\varphi_{blur,df}$ defined for isotropic df values of [2, 2, 2] and [3, 3, 3]. $\sigma =\left[\sigma_{H},\;\sigma_{W},\;\sigma_{D}\right]$ are computed as $\sigma =\frac{df-1}{2}$.
- *Degradation B*: Same as *Degradation A*, but all σ were multiplied by a factor of 1.5, i.e., the blurring effect is more pronounced for this type of degradation.
- *Degradation C*: Given an input $x\in R^{H\times W\times D}$ and a downsampling factor $df=\left[df_{H},\;df_{W},\;df_{D}\right]$, we apply the discrete Fourier transform F as ϖ= F(x) and perform the PyTorch *fftshift* (to bring the low-frequency components to the center-most locations of ϖ, obtaining $\varpi_{shift}$ ). We then crop a volume of shape $=\left[\frac{H}{df_{H}},\frac{W}{df_{W}},\frac{D}{df_{D}}\right]$ from the center-most elements of $\varpi_{shift}$ , obtaining $\varpi_{LR-shift}$ and apply the inverse shifting operation, i.e., *ifftshift*. Finally, we generate the final downsampled image with the inverse Fourier transform as:
  $$x_{LR}\;=abs\left(F^{-1}\left(\varpi_{LR-shift}\right)\right)$$
  which is the magnitude of the complex voxel-wise volume returned by $F^{-1}$ .

## 3 Performance Evaluation

The performance of RAVEN was compared against 4 state-of-the-art SISR methods, namely ArSSR-RDN (Wu et al., 2023), ArSSR-ResCNN (Wu et al., 2023), SuperFormer (Forigua et al., 2022), and NLMUPSAMPLE (Manjón et al., 2010), as well as trilinear and Lanczos interpolation. ArSSR-RDN and ArSSR-ResCNN were trained using convolutional neural networks, while SuperFormer is an implementation of Vision Transformers (ViT). NLMUPSAMPLE is a non-local patch image similarity method which iteratively optimizes the upsampled image until a convergence condition is met. RAVEN was run using 160 × 160 × 32 patches with half-patch size stride, while the rest of the methods were run using their default settings.

### 3.1 Synthetic benchmark

A synthetic benchmark was set using the degradations A, B, and C, with downsampling-upsampling factor of 2 and 3 in which Mean Absolute Error (MAE), Peak Signal to Noise Ratio (PSNR), Structural Similarity Index Measure (SSIM), and Learned Perceptual Image Patch Similarity (LPIPS) (R. Zhang et al., 2018) were computed on the foreground mask by merging all the intracranial labels produced by SynthSeg (Billot et al., 2023), after resampling the results to the native image space using nearest neighbor interpolation. In the case of LPIPS and SSIM, we computed the slice-wise metrics averaged across the three anatomical planes, and the LPIPS foreground maps were obtained by setting the “spatial” argument of the LPIPS class to “True” from the official LPIPS repository (https://github.com/richzhang/PerceptualSimilarity), computing one per anatomical plane and averaging the three per-plane maps. It is worth noting that we used the available pre-trained LPIPS weights (AlexNet version) without any additional fine-tuning.

### 3.2 Downstream segmentation of upsampled MRIs

Furthermore, to assess the impact of the methods on downstream derived measures, we generated SynthSeg masks from the upsampled MRIs and computed their similarity with the masks generated by the non-degraded native resolution HR image. We merged the bilateral Cerebral Gray Matter (GM), Cerebral White Matter (WM), Hippocampus, Ventricles, Deep GM, Cerebellum GMr, Cerebellum WM, and Brainstem to yield 8 ROIs for which we computed Dice (Dice, 1945) and IOU (Jaccard, 1901) similarity metrics. As all segmentations generated by SynthSeg have voxel sizes of 1 mm isotropic, the resulting labels were resampled using nearest neighbor interpolator to match the space of the source MRIs prior to computing the segmentation agreement measures.

### 3.3 Synthetic benchmark in out-of-distribution upsampling factors

To further evaluate the super resolution performance of the methods beyond the upsampling factors used for training, we examined their performance using an upsampling factor of 4 across all three degradation modes.

### 3.4 Application of super resolution in morphometry analysis

To examine the biological plausibility and utility of the upsampled images in common downstream tasks, we performed a deformation-based morphometry (DBM) cross-sectional analysis on in vivo T1w MRIs from the Alzheimer’s Disease Neuroimaging Initiative (ADNI) database (adni.loni.usc.edu). The ADNI was launched in 2003 as a public-private partnership, led by Principal Investigator Michael W. Weiner, MD.The original goal of ADNI was to test whether serial magnetic resonance imaging (MRI),positron emission tomography (PET), other biological markers, and clinical and neuropsychological assessment can be combined to measure the progression of mild cognitive impairment (MCI) and early Alzheimer’s disease (AD). The current goals include validating biomarkers for clinical trials, improving the generalizability of ADNI data by increasing diversity in the participant cohort, and to provide data concerning the diagnosis and progression of Alzheimer’s disease to the scientific community. For up-to-date information, see adni.loni.usc.edu. More specifically, we included a total of 443 age- and sex-matched participants with baseline diagnoses of cognitively unimpaired (CN, N=92, 48 female), subjective memory complaint (SMC, N=83, 40 female), early mild cognitive impairment (EMCI, N=88, 45 female), late mild cognitive impairment (LMCI, N=89, 45 female), and Alzheimer’s dementia (AD, N=91, 45 female). The input voxel sizes of the utilized samples (x-, y-, and z- direction steps, respectively) were 1.20 × 1.00 × 1.00 mm^3^ (N=306), 1 mm isotropic (N=98), 1.2 mm isotropic (N=56), and 1.20 × 1.30 × 1.30 mm3 (N=3). We then generated DBM maps following linear and non-linear registration of the native and high resolution images using PELICAN (Dadar et al., 2025). To achieve this, the determinant of the Jacobian matrix was calculated based on the high resolution volumes generated after upsampling the native volumes and reference templates to 0.5 mm isotropic voxel sizes using the different methods listed in Section 3.1. We then examined the differences in the voxelwise DBM values between diagnostic groups and controls using following linear regression model:

DBM~Age+Sex+Diagnosis Group

where Diagnosis Group∈{CN, SMC, eMCI, LMCI, AD}. The results were corrected for multiple comparisons using the false discovery rate (FDR) controlling method (Benjamini & Hochberg, 1995). Similar models were also completed on mean DBM values in the hippocampus ROI.

### 3.5 Statistical tests

Paired t-tests were used to compare performance of methods for each metric, followed by false discovery rate (FDR) correction (Benjamini & Hochberg, 1995). Spearman’s and Pearson’s correlations were used to examine the associations between reconstruction performance metrics and segmentation performance.

### 3.6 Ablation experiments

We further investigated the effect of different training regimes and loss variants on the performance of RAVEN. More specifically, we compared the following versions of RAVEN using the same benchmark as before (detailed in Section 3.1):

**In-vivo RAVEN:** Using the same architecture and training regime as RAVEN, we excluded all ex-vivo data from the training.
**VGG-16 RAVEN:** Using the same architecture and dataset for training as RAVEN, but replacing LPIPS with VGG16 (Ledig et al., 2017) loss as the perceptual component of our cost function. Optimization remained similar to RAVEN.
**L1 RAVEN:** Using the same architecture and dataset for training as RAVEN, but removing any perceptual losses from the training and keeping only L1 for the reconstruction term. Optimization remained similar to RAVEN.
**Augmented RAVEN:** Using the same data and training regime as RAVEN, but introducing more aggressive augmentations based on those used in SynthSeg (Billot et al., 2023): every training patch was subjected to spatial warps consisting of random 3D affine perturbations (rotations up to ±15° per axis, isotropic scaling in 0.85–1.15, shearing in ±0.02, plus small translations) followed by an elastic displacement field, intensity inhomogeneity via a low‑frequency multiplicative bias field, contrast modulation with gamma correction (γ ~ U[0.5, 1.5]), and additive zero‑mean Gaussian noise (σ ~ U[0, 10/255] in normalized units). No blurring-related degradations were applied.
**4× RAVEN:** Using the same architecture and training regime as RAVEN, but adding a factor of 4x in the possible downsampling factors during training.

### 3.7 From true LR to true HR upsampling

To assess the performance of the proposed method based on ground-truth LR-HR image pairs, we scanned stabilized post-mortem head and hemisphere specimens in LR and HR during the same scanning session and using the same acquisition parameters. This post-mortem setting allowed for no movement of the scanned specimens during the entirety of the scanning sessions, which led to perfect alignment between LR and HR acquisitions and between the two different contrasts obtained (T1w and T2w) since they were acquired during a single session. Specifically, we scanned 23 specimens at native high (T1w: 0.7 mm isotropic and T2w: 0.64 mm isotropic) and low (T1w: 1.4 mm and 2.1 mm isotropic, and T2w: 1.28 mm isotropic) resolutions. We then used the tested methods to upsample the LR images to the HR grids (i.e. ×2 and ×3 T1w, and ×2 T2w), and computed the similarity metrics (MAE, PSNR, SSIM, and LPIPS) between the two images within foreground brain masks, created by thresholding the T1w images.

## 4 Results

### 4.1 Across degradation schemes

RAVEN consistently outperformed all the tested methods on all four metrics, achieving lower LPIPS (see Fig. 3) and MAE and higher PSNR and SSIM, for degradations A–C (see detailed stratified results in Supplementary Table S3 and Supplementary Figs. S1 to S4). These results indicate that, irrespective of the degradation model, RAVEN yielded the most accurate and perceptually faithful reconstructions. Interestingly, Trilinear was generally the second-best method in terms of LPIPS for upsampling factors of 2x, with Lanczos ranking second under degradation B. For the rest of the metrics, NLMUPSAMPLE was the second-best method across factors, although Lanczos ranked second for SSIM under degradation B.

### 4.2 Across datasets

**Fig. 4.**
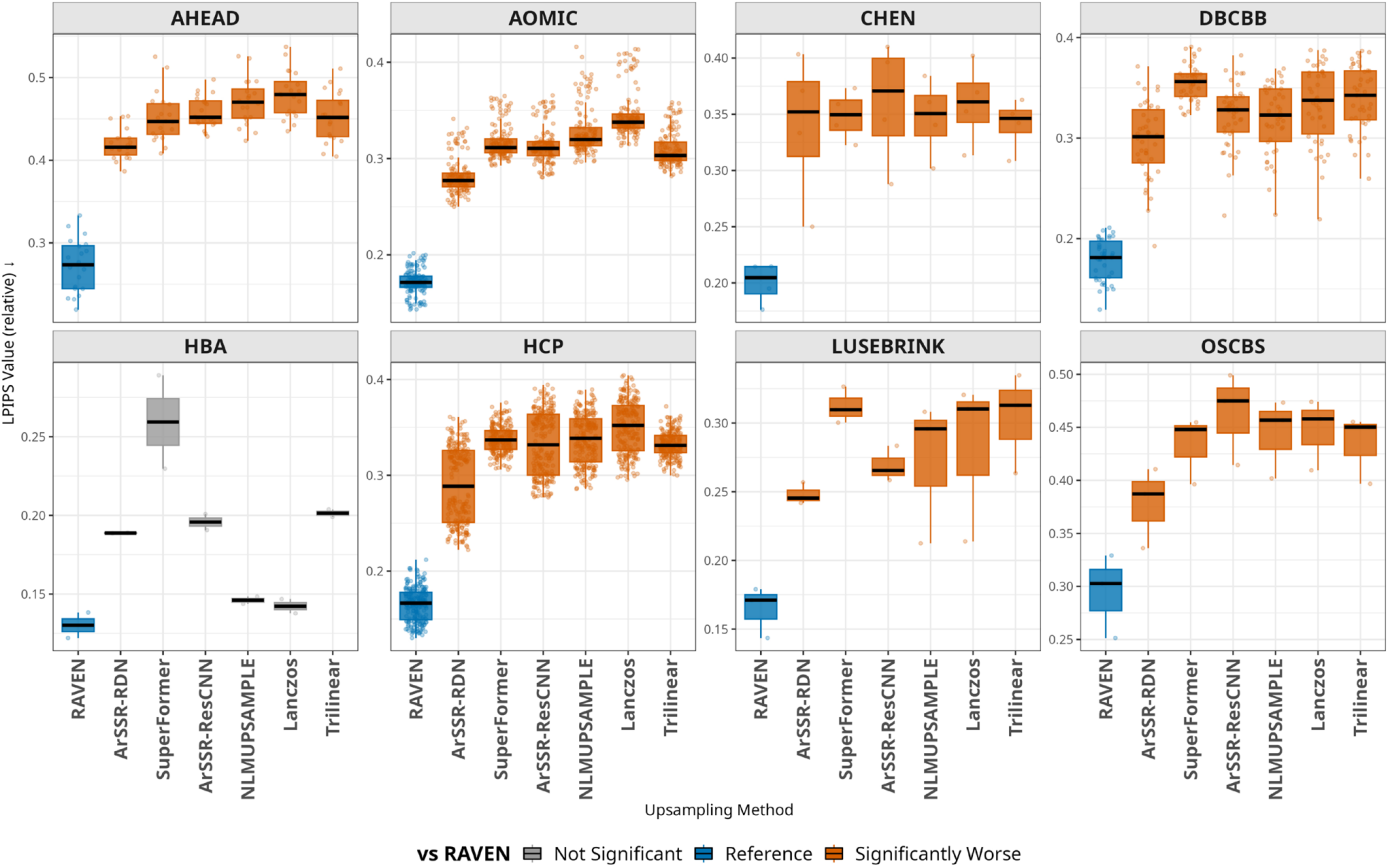
Performance per dataset as measured by LPIPS (lower is better). Boxplot colors indicate significance of paired t-test comparing other methods to RAVEN as the reference method following FDR correction. Note that the lack of statistical significance in the differences for HBA and LUSEBRINK datasets is driven by their small sample sizes, rendering the tests and results unreliable. Each datapoint represents the average metric value of an individual across the different tested degradations (A, B, and C) and upsampling factors (×2, ×3, and ×4). The color represents the significance after FDR correction (p < 0.05) from the paired t-tests on RAVEN vs each of the methods tested separately.

RAVEN was the top performer in the majority of cases. For LPIPS, RAVEN led on 7 out of 8 datasets (Fig. 4; see detailed stratified results in Supplementary Table S3 and Supplementary Figs. S1–S4), tying with NLMUPSAMPLE on HBA. ArSSR-RDN was generally second-best across degradations and datasets for upsampling factors of ×3, while for factor of ×2 the second-best method was Trilinear, with Lanczos occasionally ranking second. For MAE, it led on 5 out of 8, being outperformed by Lanczos on AHEAD and by NLMUPSAMPLE on OSCBS. For this metric, NLMUPSAMPLE was generally the second-best method; for PSNR, RAVEN led on 6 out of 8, with AHEAD favoring Lanczos and OSCBS favoring NLMUPSAMPLE; and for SSIM, RAVEN outperformed all other methods across datasets except for HBA, in which NLMUPSAMPLE and Trilinear outperformed RAVEN at × 2 upsampling factors.

### 4.3 By target voxel size

**Fig. 5.**
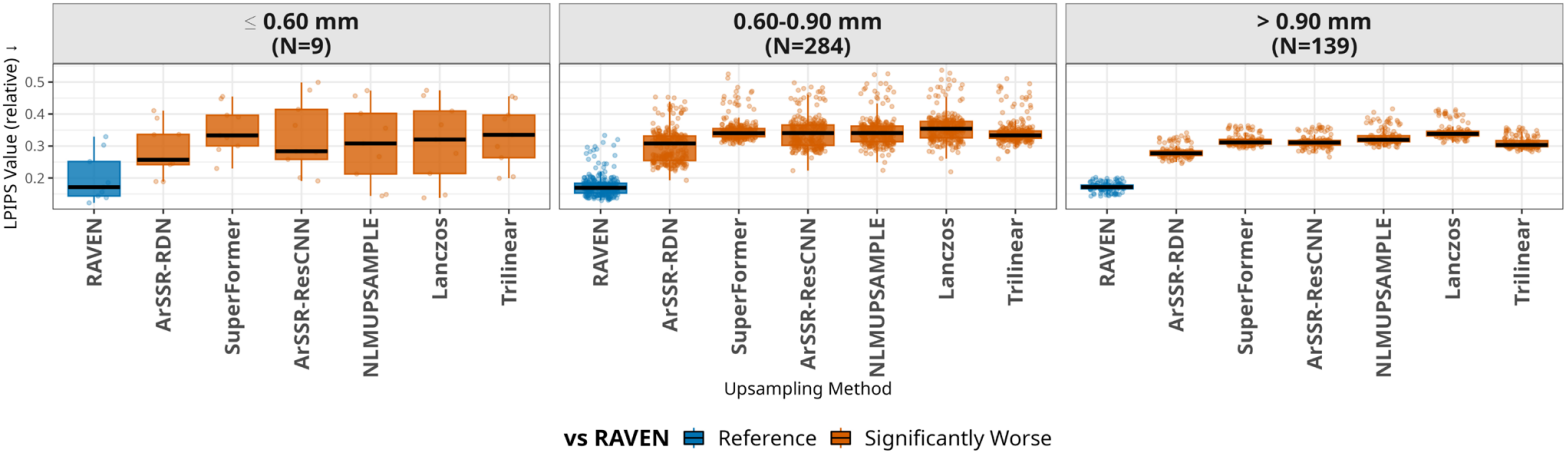
Performance per dataset as measured by LPIPS (lower is better). Boxplot colors indicate significance of paired t-tests comparing other methods to RAVEN as the reference method following FDR correction. Each datapoint represents the average metric value of an individual across the different tested degradations (A, B, and C) and upsampling factors (×2 and ×3). The color represents the significance after FDR correction (p < 0.05) from the paired t-tests on RAVEN vs each of the methods tested separately.

Stratifying by voxel size (Supplementary Table S4; Supplementary Figs. S5–S8) further supported RAVEN’s robustness. RAVEN led LPIPS across all bins (≤0.60 mm, 0.60–0.90 mm, >0.90 mm) (see Fig. 5), and achieved the best MAE in two out of three bins while matching NLMUPSAMPLE and Trilinear at ≤0.60 mm. For PSNR, RAVEN dominated at 0.60–0.90 mm and >0.90 mm, whereas NLMUPSAMPLE led at ≤0.60 mm. For SSIM, RAVEN led across all voxel bins (see detailed stratified results on Supplementary Figs. S5–S8).

### 4.4 Downstream segmentation of upsampled MRIs

**Fig. 6.**
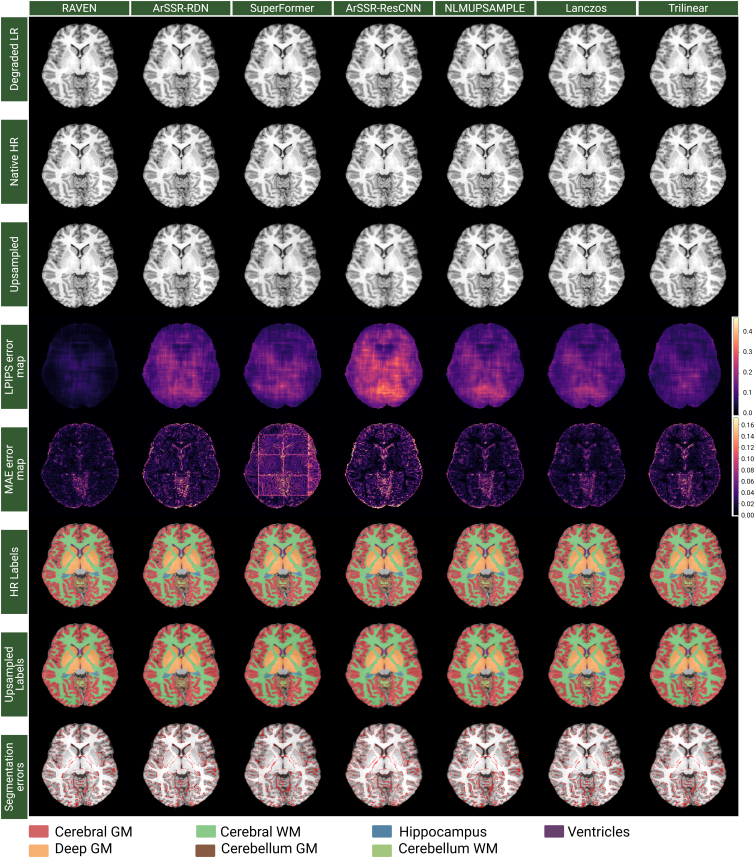
Example of an image from the HCP dataset degraded using Degradation scheme C and downsampling factor of 2 on all tested super-resolution methods. From top to bottom rows: degraded image (Native LR), non-degraded native image (Native HR), upsampled image (Native LR upsamped to Native HR space), LPIPS error map produced between Native HR and Upsampled rows, MAE error map (difference between Native HR and Upsampled rows), HR labels (SynthSeg masks merged into 8 ROIs), Upsampled LR Labels (SynthSeg masks from the Upsampled row), Difference between HR and Upsampled LR masks.

RAVEN yielded the best results across metrics, upsampling factors, datasets, degradation methods, and ROIs with 0.906 and 0.870 DICE and IOU, followed by Trilinear interpolation with 0.880 and 0.835, and SuperFormer with 0.856 and 0.801 DICE and IOU respectively. ArSSR (both resCNN and RDN versions) yielded the lowest segmentation agreement metrics with DICE of 0.815 and IOU of 0.73 for both ArSSR-ResCNN and ArSSR-RDN. For all 8 regions of interest, whole-brain LPIPS was the image similarity metric that better predicted segmentation agreement with the reference in terms of both Spearman’s and Pearson’s correlations (see Fig. 6 as well as Supplementary Fig. S9 and Fig. S10).

To further evaluate the accuracy in downstream brain volumetry using SynthSeg, we computed the volumes of 7 ROIs from the upsampled MRI labels. We then calculated the MAE between the volumes of the upsampled and reference MRIs labels in mm^3^ (Table 2). RAVEN showed superior performance across all the ROIs tested. ArSSR-RDN was second-best for Cerebral GM and Deep GM with MAE of 7490 ± 7576 and 383 ± 346 mm^3^, respectively. SuperFormer was second-best for Cerebellar GM with MAE of 1525 ± 1322 mm^3^. NLMUPSAMPLE performed second-best for Hippocampus and Ventricles with MAE Of 163 ± 140 and 294 ± 314 mm^3^, respectively. Trilinear interpolation was second best for Cerebellar WM and Cerebral WM with MAE of 659 ± 490 and 2455 ± 2066 mm^3^, respectively.

**Table 2.** MAE between ROI volumes based on reference and upsampled MRIs for all upsampling methods.

Method	Cerebellar GM [mm^3^]	Cerebellar WM [mm^3^]	Cerebral GM [mm^3^]	Cerebral WM [mm^3^]	Deep GM [mm^3^]	Hippocampus [mm^3^]	Ventricles [mm^3^]
RAVEN	**870.02 ± 982.14**	**268.66 ± 246.63**	**5291.70 ± 5294.30**	**1743.21 ± 1193.11**	**218.78 ± 209.20**	**65.65 ± 54.32**	**180.50 ± 183.36**
ArSSR-RDN	1665.50 ± 1516.74	712.17 ± 569.68	7489.69 ± 7575.70	3884.98 ± 3009.52	382.56 ± 345.83	180.16 ± 155.05	604.83 ± 489.03
SuperFormer	1525.43 ± 1322.49	693.30 ± 585.53	8543.50 ± 7311.95	5240.46 ± 4609.20	869.41 ± 729.28	205.63 ± 166.95	575.04 ± 479.61
ArSSR-ResCNN	3466.83 ± 2979.10	725.64 ± 534.16	20255.86 ±15222.10	4579.30 ± 4113.40	1196.99 ± 877.62	181.28 ± 146.65	891.57 ± 634.71
NLMUPSAMPLE	3146.30 ± 2836.93	671.92 ± 534.05	16481.81 ± 14218.64	2697.84 ± 2058.71	692.10 ± 662.86	162.78 ± 140.33	293.78 ± 313.90
Lanczos	2808.02 ± 2690.14	744.48 ± 631.10	14364.39 ± 12690.58	3815.65 ± 2753.57	553.18 ± 585.54	166.17 ± 153.25	333.98 ± 361.18
Trilinear	3535.48 ± 2908.90	659.34 ± 490.41	20994.84 ± 13970.72	2454.54 ± 2065.80	838.25 ± 695.94	184.67 ± 147.22	396.41 ± 428.83

The best and second-best performing methods for each ROI are indicated as bold and underlined, respectively. Values are displayed as “mean ± standard deviation”, with lower values indicating better performance.

### 4.5 ×4 upsampling factor

**Fig. 7.**
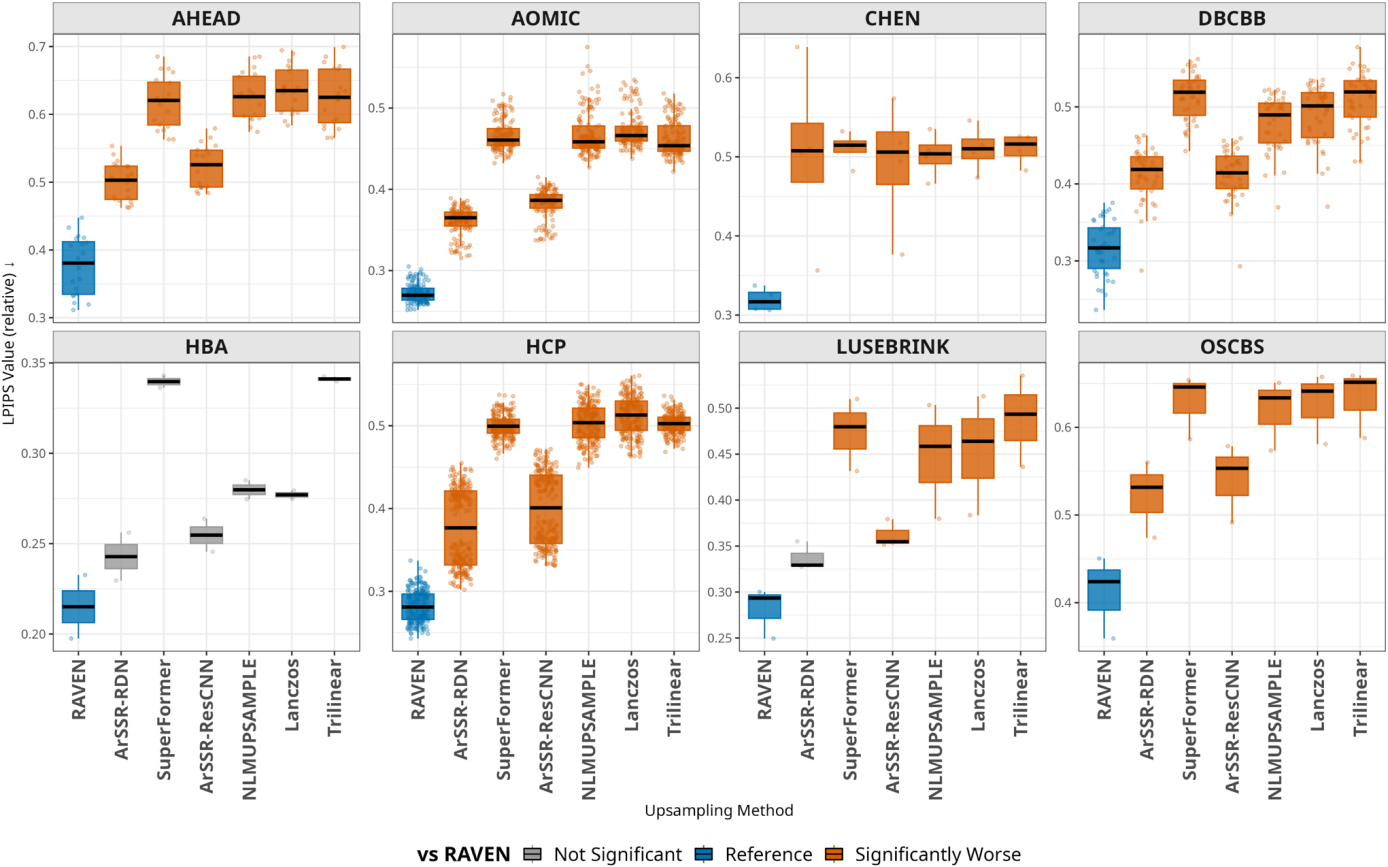
Out-of-distribution performance of each method (upsampling factor: 4) as measured by LPIPS (lower is better). Boxplot colors indicate significance of paired t-tests comparing other methods to RAVEN as the reference method following FDR correction.

RAVEN outperformed its counterparts in the out-of-distribution test for 4 isotropic upsampling factor using degradations A, B, and C across all datasets (Fig. 7). RAVEN achieved the best overall performance, yielding the lowest LPIPS (0.287 ± 0.033), followed by ArSSR-RDN (0.382 ± 0.052), whereas the remaining approaches exhibited substantially lower performances, i.e., higher LPIPS values (>0.4). In contrast, for distortion-oriented fidelity metrics, Lanczos provided the strongest reconstruction accuracy, attaining the lowest MAE (0.093 ± 0.025) and the highest PSNR (25.70 ± 2.30), with NLMUPSAMPLE closely trailing on both (MAE 0.095 ± 0.026; PSNR 25.56 ± 2.32). For structural similarity, Lanczos marginally outperformed RAVEN on SSIM (0.517 ± 0.057) whereas RAVEN obtained SSIM of 0.510 ± 0.058. Detailed results are provided in Supplementary Table S2.

### 4.6 From true LR to true HR data

**Fig. 8.**
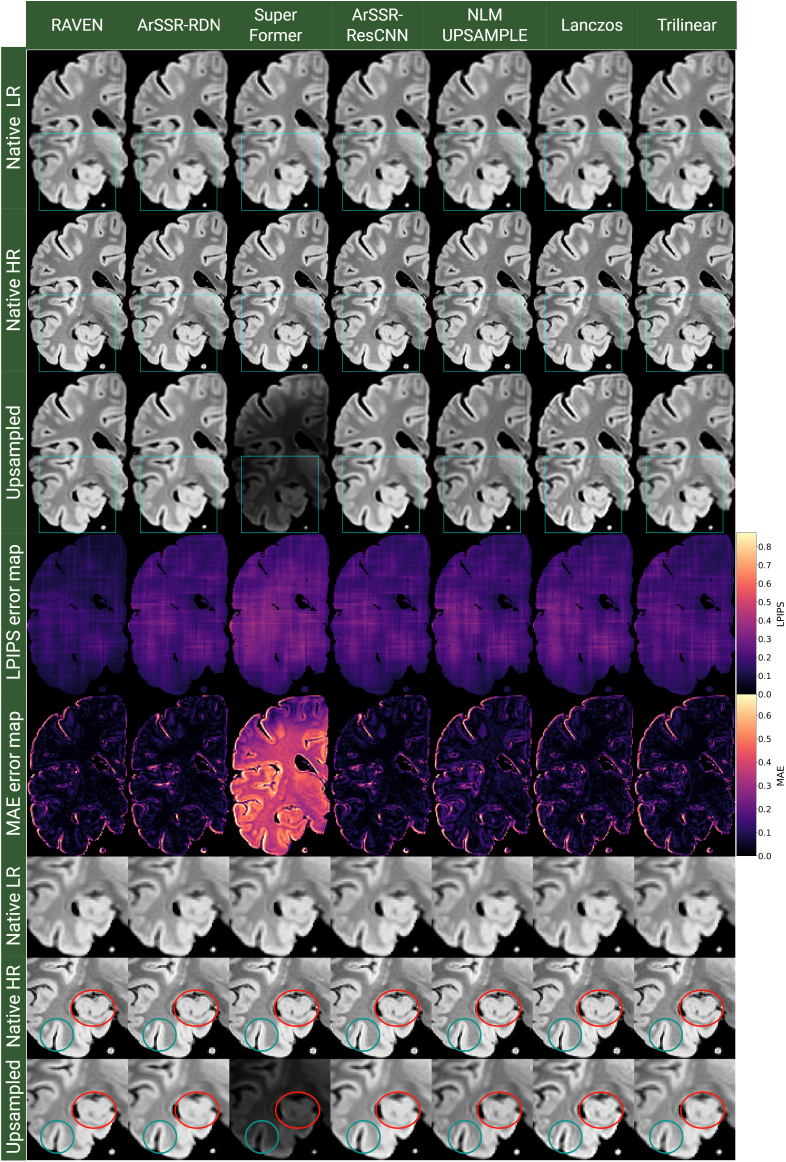
Example of real-world gold-standard post-mortem T1w MRI upsampling. The same post-mortem specimen was scanned during the same session at 1.4 mm isotropic (Native LR rows) and at 0.7 mm isotropic (Native HR rows). Different methods were applied to upsample the Native LR by a factor of 2 in all anatomical axes and then compared to the ground-truth high-resolution samples. RAVEN shows lower LPIPS and MAE values (fourth and fifth rows) as well as sharper and more similar details to the reference ground truth.

RAVEN showed the strongest overall performance across metrics and contrasts and upsampling factors (Fig. 8 and Table 3; see Supplementary Table S5 for detailed results). It achieved the lowest LPIPS in all three settings, with mean values of 0.20, 0.29, and 0.17 for T1w ×2, T1w ×3, and T2w ×2 upsampling, respectively. Performance differences between RAVEN and the rest of the methods remained significant after multiple-comparison correction (q < 0.0001). RAVEN also yielded the highest SSIM across all three settings, reaching 0.58, 0.46, and 0.72 for T1w ×2, T1w ×3, and T2w ×2, respectively. For PSNR, RAVEN was best in T1w ×2 and T2w ×2, with 25.61 and 30.00 dB, while NLMUPSAMPLE was slightly better in T1w ×3 with 22.87 dB versus 22.45 dB for RAVEN. A similar pattern was observed for MAE: RAVEN achieved the lowest error in T1w ×2 and T2w ×2, with 0.10 and 0.06, whereas NLMUPSAMPLE obtained a marginally lower MAE in T1w ×3.

**Table 3.** Full results for true LR to true HR super-resolution using different upsampling methods on postmortem MRI data.

Metric	Method	T1w ×2 (1.4 mm iso → 0.7 mm iso)	T1w ×3 (2.1 mm iso → 0.7 mm iso)	T2w ×2 (1.28 mm iso → 0.64 mm iso)
LPIPS ↓	RAVEN	**0.20 ± 0.08**	**0.29 ± 0.08**	**0.17 ± 0.04**
	ArSSR-RDN	0.25 ± 0.08 (q = <0.0001)	0.35 ± 0.08 (q = <0.0001)	0.24 ± 0.03 (q = <0.0001)
	SuperFormer	0.31 ± 0.07 (q = <0.0001)	0.40 ± 0.06 (q = <0.0001)	0.29 ± 0.03 (q = <0.0001)
	ArSSR-ResCNN	0.25 ± 0.08 (q = <0.0001)	0.35 ± 0.08 (q = <0.0001)	0.25 ± 0.03 (q = <0.0001)
	NLMUPSAMPLE	0.23 ± 0.07 (q = <0.0001)	0.35 ± 0.07 (q = <0.0001)	0.24 ± 0.04 (q = <0.0001)
	Lanczos	0.23 ± 0.07 (q = <0.0001)	0.37 ± 0.07 (q = <0.0001)	0.26 ± 0.04 (q = <0.0001)
	Trilinear	0.25 ± 0.07 (q = <0.0001)	0.38 ± 0.07 (q = <0.0001)	0.26 ± 0.03 (q = <0.0001)
PSNR (dB) ↑	RAVEN	**25.61 ± 4.83**	22.45 ± 3.45	**30.00 ± 2.81**
	ArSSR-RDN	24.63 ± 3.88 (q = 0.0632)	20.97 ± 2.87 (q = <0.0001)	27.67 ± 2.81 (q = 0.0063)
	SuperFormer	13.80 ± 5.08 (q = <0.0001)	13.88 ± 4.79 (q = <0.0001)	24.19 ± 2.35 (q = <0.0001)
	ArSSR-ResCNN	24.90 ± 4.02 (q = 0.1541)	21.12 ± 2.94 (q = <0.0001)	27.07 ± 2.44 (q = <0.0001)
	NLMUPSAMPLE	25.03 ± 3.74 (q = 0.1690)	**22.87 ± 3.48 (q = 0.1541)**	28.65 ± 2.54 (q = 0.0002)
	Lanczos	24.93 ± 3.90 (q = 0.1538)	21.77 ± 3.11 (q = 0.0683)	29.04 ± 2.67 (q = <0.0001)
	Trilinear	24.69 ± 3.91 (q = 0.0070)	21.64 ± 2.92 (q = 0.0057)	29.38 ± 2.61 (q = 0.0002)
SSIM ↑	RAVEN	**0.58 ± 0.17**	**0.46 ± 0.13**	**0.72 ± 0.14**
	ArSSR-RDN	0.56 ± 0.16 (q = 0.0318)	0.40 ± 0.11 (q = <0.0001)	0.66 ± 0.13 (q = 0.0004)
	SuperFormer	0.37 ± 0.15 (q = <0.0001)	0.30 ± 0.10 (q = <0.0001)	0.57 ± 0.11 (q = <0.0001)
	ArSSR-ResCNN	0.57 ± 0.16 (q = 0.0927)	0.41 ± 0.11 (q = 0.0002)	0.63 ± 0.11 (q = <0.0001)
	NLMUPSAMPLE	0.56 ± 0.16 (q = <0.0001)	0.45 ± 0.13 (q = <0.0001)	0.70 ± 0.13 (q = <0.0001)
	Lanczos	0.57 ± 0.17 (q = <0.0001)	0.45 ± 0.13 (q = <0.0001)	0.70 ± 0.13 (q = <0.0001)
	Trilinear	0.54 ± 0.15 (q = <0.0001)	0.42 ± 0.11 (q = <0.0001)	0.69 ± 0.12 (q = <0.0001)
MAE ↓	RAVEN	**0.10 ± 0.06**	0.13 ± 0.06	**0.06 ± 0.03**
	ArSSR-RDN	0.10 ± 0.06 (q = 0.5536)	0.14 ± 0.05 (q = <0.0001)	0.08 ± 0.03 (q = 0.0048)
	SuperFormer	0.28 ± 0.10 (q = <0.0001)	0.28 ± 0.09 (q = <0.0001)	0.10 ± 0.03 (q = <0.0001)
	ArSSR-ResCNN	0.10 ± 0.06 (q = 0.8313)	0.14 ± 0.05 (q = 0.0001)	0.08 ± 0.03 (q = <0.0001)
	NLMUPSAMPLE	0.10 ± 0.05 (q = 0.4831)	**0.13 ± 0.06 (q = 0.2149)**	0.07 ± 0.03 (q = <0.0001)
	Lanczos	0.10 ± 0.06 (q = 0.4507)	0.13 ± 0.06 (q = 0.1201)	0.07 ± 0.03 (q = <0.0001)
	Trilinear	0.11 ± 0.05 (q = 0.1174)	0.14 ± 0.05 (q = 0.0222)	0.07 ± 0.03 (q = 0.0002)

Results presented as “mean ± standard deviation (p-value of t-test vs RAVEN after FDR correction)”. In bold the measurement with the best mean value.

Finally, to extract MRI-derived metrics after applying super-resolution, we applied SynthSeg (Fig. 7) and generated 8 regions of interest (ROI): Cerebral and cerebellar Gray and White matter, hippocampus, ventricles, deep Gray matter, and brainstem. We observed consistent results across the performed tests: RAVEN obtained the best performance across ROIs with mean DICE and IOU of 0.91 (±0.03) and 0.83 (±0.05) respectively, followed by trilinear interpolation with 0.90 (±0.031) and 0.83 (±0.052) and then SuperFormer with 0.89 (±0.032) and 0.81 (±0.051) DICE and IOU respectively.

### 4.7 Application of super resolution in morphometry analysis

**Fig. 9.**
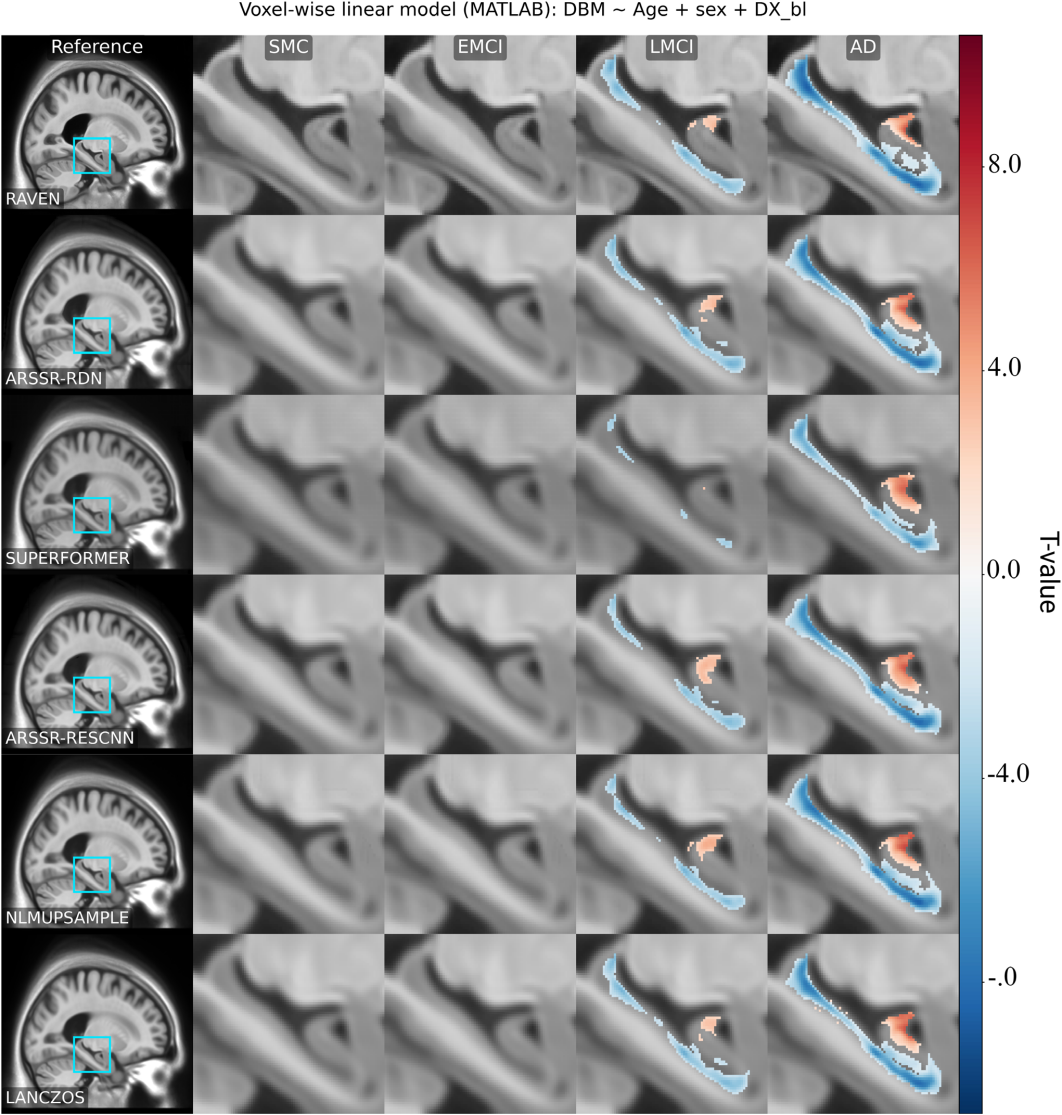
Voxel-wise t-maps (p_FDR_ < 0.05) of the hippocampus obtained from the linear model *DBM ~ Age + Sex + Diagnosis Group* contrasting SMC, EMCI, LMCI, and AD groups against controls, using DBM values obtained from the upsampled MRIs using different upsampling methods (rows) across diagnostic groups (columns). The blue box in the left column indicates the region of the full sagittal slice shown in the zoomed-in panels in the remaining columns.

When mean DBM values within the hippocampal mask were compared using linear models adjusted for age and sex (Table 4), no method showed a significant SMC versus CN effect (all p > 0.05). For EMCI, ArSSR-ResCNN and RAVEN showed significant EMCI versus CN effects, with t-values of -2.29 (p = 0.022) and -2.05 (p = 0.041), respectively. For LMCI, significant effects versus CN were observed for all methods except SuperFormer (p = 0.077), and Lanczos and RAVEN showed the largest absolute t-values (-3.56 and -3.07, respectively). For AD, all methods showed significant AD versus CN effects, with RAVEN and NLM-Upsample yielding the largest absolute t-values (-7.47 and -7.29, respectively). Figure 9 shows the voxelwise t statistics maps of the significant group differences (model described in Section 3.4), thresholded at p_fdr_ < 0.05. Negative t-values reflecting significantly smaller volumes are indicated in blue, and positive t-values reflecting larger volumes (due to partial volume effects) are indicated in red.

**Table 4.** Results from the linear model *DBM_mean_ROI_ ~ Age + Sex + Diagnosis Group* for mean DBM within the hippocampal mask, with SMC, EMCI, LMCI, and AD compared against the CN reference group.

	SMC			EMCI			LMCI			AD		
Method	t-val	p-val	cohen’s d	t-val	p-val	cohen’s d	t-val	p-val	cohen’s d	t-val	p-val	cohen’s d
NLMUPSAMPLE	-0.659	0.511	-0.111	-1.598	0.111	-0.25	-2.868	**0.004**	-0.433	-7.291	**0**	-1.045
RAVEN	-1.066	0.287	-0.173	-2.05	**0.041**	-0.32	-3.074	**0.002**	-0.468	-7.471	**0**	-1.068
SuperFormer	-0.85	0.396	-0.132	-1.535	0.126	-0.213	-1.773	0.077	-0.27	-4.137	**0**	-0.63
ArSSR-RDN	-0.25	0.803	-0.043	-0.91	0.363	-0.142	-2.868	**0.004**	-0.396	-6.299	**0**	-0.853
ArSSR-ResCNN	-1.157	0.248	-0.186	-2.291	**0.022**	-0.332	-2.19	**0.029**	-0.446	-5.795	**0**	-1.013
Lanczos	-1.089	0.277	-0.175	-1.315	0.189	-0.245	-3.557	**0**	-0.498	-6.497	**0**	-1.115

Significant p-values after FDR-correction (<0.05) are indicated in bold.

### 4.8 CrossResolutionAttention3D contribution on speed and memory

**Fig. 10.**
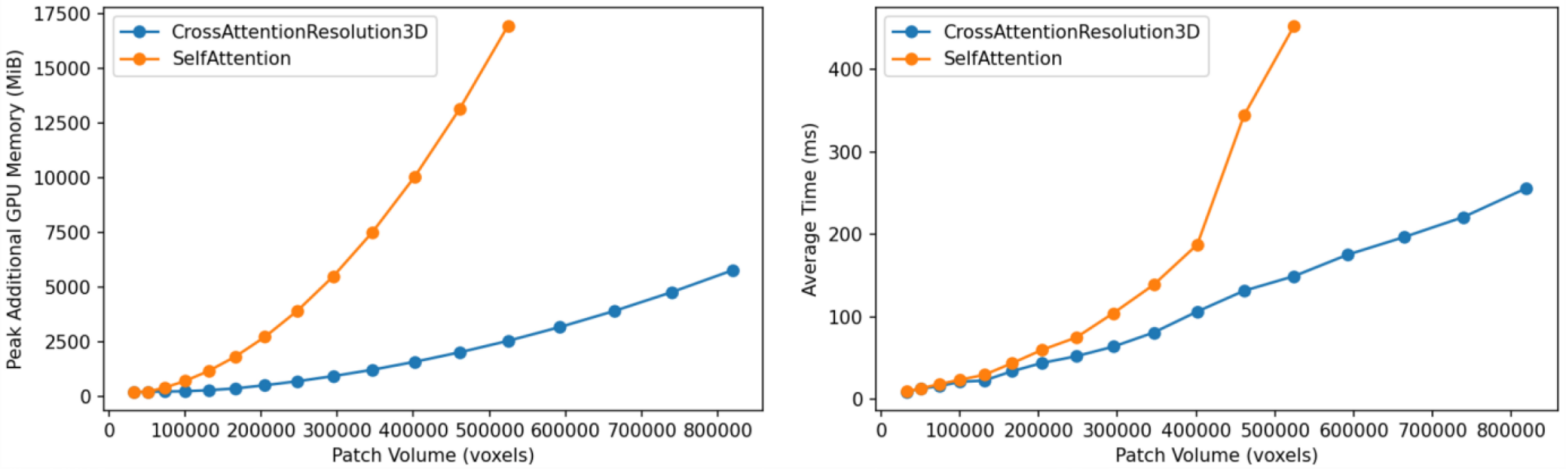
Comparison between full forward passes using CrossResolutionAttention3D and full-resolution Self Attention using different input patch volumes. Left: peak additional GPU memory after loading the weights. Right: forward inference time after the input patch was preprocessed and loaded into GPU.

We assessed the computational cost and time of using our implementation of the proposed CrossResolutionAttention3D module versus a standard self-attention module. To achieve this, we benchmarked an end-to-end patch processing pipeline using an increasing volume of voxels and compared our attention module to its counterpart without the pooling layer as an equivalent to a standard self-attention module. We measured the added VRAM burden to the baseline (only model loaded into the GPU) and the total processing time per patch (Fig. 10). As can be noted, input patch volume of 591,872 voxels leads to OOM errors (using an RTX 4090 GPU with 24 GB of VRAM) for the self-attention module network, while using CrossResolutionAttention3D allows for increasing the input patch size beyond 800k voxels while keeping the GPU usage low and is also faster than the maximum patch volume supported by self-attention (~600k voxels).

### 4.9 Ablation study

Ablation variants (RAVEN, 4× RAVEN, L1 RAVEN, VGG-16 RAVEN, Augmented RAVEN, In-vivo RAVEN) were evaluated at ×2 and ×3 using LPIPS, MAE, PSNR, and SSIM (mean ± SD) (Table 5). At ×2, RAVEN yielded the lowest LPIPS (0.059 ± 0.024) and MAE (0.030 ± 0.016) and the highest PSNR (35.607 ± 3.267), while L1 RAVEN attained the highest SSIM (0.902 ± 0.038); second‑best values were Invivo RAVEN for LPIPS (0.062 ± 0.024), L1 RAVEN for MAE (0.031 ± 0.017) and PSNR (35.607 ± 3.505), and RAVEN for SSIM (0.902 ± 0.040). At ×3, LPIPS was lowest for In-vivo RAVEN (0.167 ± 0.044), MAE and PSNR were best for RAVEN (0.048 ± 0.022; 31.339 ± 3.069), and SSIM was highest for VGG-16 RAVEN (0.806 ± 0.046); runner‑up results were VGG-16 RAVEN for LPIPS (0.167 ± 0.040), In-vivo RAVEN for MAE (0.050 ± 0.022) and PSNR (30.996 ± 3.037), and In-vivo RAVEN for SSIM (0.801 ± 0.048). In the global analysis, In-vivo RAVEN yielded the lowest LPIPS (0.117 ± 0.065), whereas RAVEN was best overall for MAE (0.040 ± 0.022), PSNR (33.345 ± 3.934), and SSIM (0.849 ± 0.070); VGG-16 RAVEN was the closest competitor for LPIPS/SSIM and L1 RAVEN for MAE/PSNR.

**Table 5.** Ablation study for different versions of RAVEN.

Method	LPIPS (mean ± SD)	MAE (mean ± SD)	PSNR (mean ± SD) dB	SSIM (mean ± SD)
RAVEN	0.118 ± (0.031)	**0.039 ± (0.019)**	**33.494 ± (3.087)**	**0.851 ± (0.043)**
4× RAVEN	0.125 ± (0.034)	0.043 ± (0.017)	32.286 ± (2.609)	0.836 ± (0.042)
L1 RAVEN	0.158 ± (0.039)	0.041 ± (0.019)	33.305 ± (3.205)	0.841 ± (0.042)
VGG-16 RAVEN	0.116 ± (0.031)	0.046 ± (0.016)	31.444 ± (2.727)	0.846 ± (0.041)
Augmented RAVEN	0.124 ± (0.038)	0.042 ± (0.020)	32.631 ± (2.948)	0.839 ± (0.044)
In-vivo RAVEN	**0.115 ± (0.033)**	0.042 ± (0.019)	32.530 ± (2.937)	0.845 ± (0.042)

Bold and underlined values indicate best and second-best values for each metric, respectively.

## 5 Discussion

In this work, we presented RAVEN, an open-source, resolution and contrast agnostic, SISR method. We demonstrated that RAVEN obtains competitive results when compared against other state-of-the-art methods across degradation modes, resolutions, and upsampling factors. RAVEN also outperformed other methods with respect to downstream task performance: the segmentations generated by SynthSeg on the upsampled volumes were more similar to the reference ground truth than those obtained with interpolation. RAVEN obtained higher overall segmentation performance across metrics, downsampling factors, degradation modes, and datasets, indicating higher fidelity in tissue reconstruction in high-resolution domains.

Despite being a 3D model, RAVEN’s patch-based inference design allows it to run on domestic-grade GPUs with less than 24 GB. Additionally, to limit the peak VRAM usage through the patch-based inference, the inclusion of the CrossResolutionAttention3D module allows for a pseudo-self-attention operation for global context inclusion using 8x less memory, making it a suitable option for its usage in contexts where high-performance clusters are out of reach. In concrete, replacing self-attention (Esser et al., 2021) with our CrossResolutionAttention3D module allowed processing relatively-large patches using ~7-8x less GPU memory in at least ~30% less time. As a reference, a Latent Diffusion Model that uses a similar architecture as ours, but using self-attention modules was described in Pinaya et al. (2022), in which the minimum GPU memory for inference was 80 GB, making it prohibitive at large scale or in environments outside the high-performance-compute regime.

Degradation C (k-space cropping) follows MRI-physics more closely than gaussian blurring followed by downsampling and should be used as a degradation function for generating LR-HR image pairs. While the first statement is true, there is evidence in the SISR literature (Rombach et al., 2022; K. Zhang et al., 2021) that supports the usage of a diverse set of degradations with multiple blurring and downsampling operations in favor of better generalizability. We posit that simply cropping the k-space as it is done in recent literature (Wu et al., 2023; Zhao et al., 2025) does not provide sufficient input variability. These observations are supported by the results reported in this work, in which our simple but varied set of degradations applied during training enabled a well-behaved network when dealing with unseen degradations as shown by the performance metrics on Degradation C.

While ArSSR-RDN (Wu et al., 2023), ArSSR-ResCNN (Wu et al., 2023), and SuperFormer (Forigua et al., 2022) are recent state-of-the-art methods, only ArSSR-RDN consistently improved reconstruction over trilinear interpolation among the tested deep-learning algorithms. This improvement, however, was observed only at 3 × 3 × 3 upsampling factors. This is likely since the method was trained using upsampling factors in the range of [2-4] and performed better at 3 × 3 × 3 (i.e. mean of the selected range) compared to 2 × 2 × 2 in the results reported in the original article as well. We hypothesize that optimizing for voxel-space metrics and using a single dataset with only T1w images are detrimental to generalizability across other datasets and upsampling factors.

All metrics were carefully quality-controlled, and our procedure was adapted to produce robust measurements: only voxels corresponding to brain tissue were considered; robust outlier cropping was applied uniformly to all images (clamping intensities to the 0.5th and 99.5th percentiles); and LPIPS and SSIM were computed as the average across the three anatomical planes. This procedure prevents the metrics from being severely affected by outliers.

Another relevant contribution of this work is demonstrating that LPIPS is a superior image-quality metric that better detects true structural differences than voxel-space, intensity-based metrics such as PSNR and MAE. LPIPS showed the highest correlation with DICE and IOU, followed by SSIM, in our downstream segmentation tests with SynthSeg (Supplementary Fig. S10). Although RAVEN did not outperform all methods across all scenarios for MAE or PSNR, it achieved the best LPIPS and the highest IOU and DICE. These findings support the view that deep-feature similarity metrics and loss functions better preserve true anatomy, and suggest that a future 3D-LPIPS distance for medical-image applications could further improve robustness. Our findings showing better correlations between LPIPS and segmentation quality (Supplementary Fig. S10) are in line with recent literature comparing different image quality metrics for MR reconstruction (Adame-Gonzalez et al., 2024; Adamson et al., 2025; Dohmen et al., 2025; Yang et al., 2018; Ye et al., 2023). In Adamson et al. (2025), it was reported that deep feature distance metrics such as LPIPS outperform commonly used image quality metrics like PSNR and MAE in radiologist-perceived diagnostic image quality and have similar or better radiologist inter-observer variability. Additionally, other studies on medical image enhancement (Adame-Gonzalez et al., 2024; Dohmen et al., 2025) and non-medical image super-resolution tasks (Ye et al., 2023) have reported higher agreement with human visual perception when using LPIPS compared to PSNR, MAE, or SSIM. Nevertheless, we have also provided detailed results across these metrics, which provides the necessary information for future comparison for those interested in evaluating the correspondence across these metrics or in using them to compare the methods used in the current manuscript.

Our loss function was designed with fine-detail reconstruction in mind (small structures, high-frequency components); thus, LPIPS was the dominant component of the reconstruction loss. An L1 term was added to prevent shifts in the overall intensity distribution. Because the network uses 3D patches while LPIPS is inherently 2D, we adapted the LPIPS component to compute a loss for each anatomical plane and average the three per-plane values, regularizing the network and preventing slicing artifacts that could lead to a “jagged” appearance on perpendicular planes. GAN training is known to present multiple challenges, especially with training dynamics. The best stability was achieved by training the generator for two epochs without a discriminator and then introducing the discriminator. Additionally, tuning the contribution of the adaptive weight was the second most crucial factor for a well-behaved, convergent network.

Note that all generated results for all networks were obtained using the same set of author-released pretrained weights and no test-specific retraining was performed. Thus, the generalization capability of a single set of weights per method was tested across all resolutions and MRI modalities described above. Furthermore, ArSSR-RDN (Wu et al., 2023), ArSSR-ResCNN (Wu et al., 2023), and SuperFormer (Forigua et al., 2022) were trained and validated based on the HCP dataset in the original articles, and as such, the results based on the HCP dataset reflect their within sample performance, and the other datasets can be considered out of sample. Importantly, even in this within sample comparisons, RAVEN outperformed all these models. We provided examples of the methods’ performances for 1 HCP image in Figure 3, to demonstrate RAVEN’s superior performance even in the dataset the other methods had specialized on. Additionally, Supplementary Table S2 and Supplementary Figures S1–S4 provide detailed information about the results obtained per method and further stratified by experiment (dataset, degradation mode, and upsampling factor).

It is worth noting that the alternative DL-based methods (ArSSR-RDN, ArSSR-RDN, and SuperFormer) were generally outperformed by Lanczos, Trilinear and NLMUPSAMPLE at upsampling scale of 2 × 2 × 2 even on the same HCP data they were trained on. This is in line with the findings reported by Wu et al. (2023) in the ArSSR article, where they found ArSSR underperforming in HCP dataset using an upsampling factor of 2 across all metrics (including LPIPS). They reported increased performance over other methods with upsampling factors of 3 and above, which matched our own results. Other DL-based methods in general outperformed Lanczos, Trilinear, and NLMUPSAMPLE in experiments with factors of 3 and 4. One potential explanation may come from the optimization process during training; since the largest errors come from larger upsampling factors and the gradients for optimizing the weights are directly linked to these errors, the models may be biased towards performing better for higher factors than for lower factors if the optimization has not reached its global minima, for example, in early stages of the training process. In the case of SuperFormer, we noticed patch-artifacts every 64 voxels, matching the input patch size for the model. Despite this, we noticed high MAE values (e.g. error maps in Fig. 6), from voxels in central patch positions which rules out “stitching” artifacts as explanation for SuperFormer’s poor performance. We hypothesize that SuperFormer may introduce intensity shifts (see Supplementary Fig. S11), and that adopting and optimizing a patch-merging strategy can potentially reduce patch artifacts and increase the performance of this method. Additionally, SuperFormer was trained using a degradation scheme identical to Degradation C with a factor of 2 × 2 × 2 (Forigua et al., 2022). From the data in Supplementary Table S3, we can notice that SuperFormer outperforms Lanczos and NLMUPSAMPLE with Degradation C and factor of 2 × 2 × 2 in HCP dataset, which may indicate a network optimized for these specific degradation conditions. Finally, DL-based methods overall outperformed Lanczos, NLMUPSAMPLE, and Trilinear interpolation in upsampling factors beyond 2 × 2 × 2.

Our ablation study showed that RAVEN was the best method for 3 out of 4 metrics, and was 3rd best in LPIPS falling behind RAVEN-Invivo and RAVEN-VGG16. RAVEN-L1 was the worst ablated network without surprise given that it did not use any perceptual component in its loss function, and apart from this network, all other ablated versions performed similarly (no drastic drop of performance from any ablated version). This confirms robustness of the design to different optimization choices.

Additionally, our ×4 upsampling test (Section 4.5) showed that RAVEN performs well even in out-of-distribution downsampling factors. It outperformed significantly the alternative methods, with ArSSR-RDN and ArSSR-ResCNN in second and third best consistently.

Furthermore, our true LR to true HR test (Section 4.6) shows that RAVEN outperforms the alternative methods in terms of LPIPS, and is tied as best method in terms of MAE, but NLMUPSAMPLE is consistently the best or second best in PSNR and SSIM.

Finally, our hippocampal DBM analysis in an AD population (Section 4.7) showed that DBM maps derived from RAVEN outputs yielded the largest or second-largest differences between cognitively impaired and cognitively unimpaired groups. Given that atrophy of the hippocampal formation is a well-established hallmark of AD (De Flores et al., 2015; Fjell et al., 2014; Morais‐Ribeiro et al., 2024; Mortimer et al., 2004; Rao et al., 2022; Yushkevich et al., 2015), these findings suggest that the sub-millimeter DBM maps generated from RAVEN outputs retain biologically meaningful anatomical variations relevant to AD-related neurodegeneration.

This work is not without limitations. The most evident limitation is the lack of substantial numbers of true LR–HR image pairs acquired at native low and high resolutions, which would be ideal for network training. Using the proposed degradation functions during training was one strategy to boost RAVEN’s generalization performance. Other strategies for domain randomization have been described in the literature. For example, Zhang et al. (2021) proposed that second order degradation models (applying a series of invertible and non invertible transformations to an image already degraded) could boost the performance of super-resolution models. They introduced a “random shuffle” strategy of degradations, and limited the type of degradations to blur, downsampling, and noise, which are commonly found in data acquired with camera sensors. However, our ablation experiments showed that adding SynthSeg-like (Billot et al., 2023) augmentations does not substantially change the performance of RAVEN. In fact, the different ablated versions of RAVEN (see Table 4) showed a consistent performance across different metrics.

We acknowledge that the LPIPS error maps appear to have cross artifacts, and this is consistent with LPIPS maps reported in other studies (Andersson et al., 2020; Hermann et al., 2025). In LPIPS, feature maps of the input pairs are computed at different depths of the networks, which implies deeper features will have smaller spatial resolutions. The difference maps between the two inputs are computed and resampled to the spatial resolution of the input and then averaged to produce the final LPIPS error maps. If the errors come predominantly from deeper layers rather than early layers, the resampled maps will contribute in a larger extent to the final cross artifact appearance of the map. To the best of our understanding, this is a measurement of how different abstract features are in the latent space, rather than localized voxel-wise difference in the image space.

Alternatively, if one had access to a sufficiently large, varied set of native LR–HR MRI pairs, one could train a GAN that generates degradations to better simulate LR MRIs from HR counterparts and then train a SISR network using those synthetic outputs. Another limitation is model size: larger models typically perform better. Although our environment would allow training larger models, and despite our CrossResolutionAttention3D enabling a more efficient attention mechanism, we opted to design a model that can be trained locally on an NVIDIA RTX 4090 GPU—a relatively low-cost, non-research-grade GPU—to optimize resources, enable efficient deployment by most medical-image processing research groups worldwide, and promote access to state-of-the-art tools in low-income countries. We, therefore, acknowledge that a version of RAVEN with more filters per convolutional layer would likely achieve higher performance in less-constrained environments.

Additionally, we recognize that alternative state-of-the-art methods for MRI SISR exist. The denoising diffusion probabilistic model (DDPM) family has gained popularity as it has demonstrated the ability to outperform GANs and has been applied to brain MRI SISR (J. Wang et al., 2023; Zhao et al., 2025). However, these models typically require substantial computational resources, often operate on 2D slices, may not directly increase in-plane resolution, and, by design, rely on iterative inference along an inverse diffusion Markov chain. An alternative that could enable faster inference is latent flow matching (Fischer et al., 2024). Exploring such iterative algorithms remains future work.

In this work, we proposed and validated RAVEN, a robust, generalizable, contrast-agnostic, and efficient deep-learning method for brain MRI SISR. RAVEN is open-source with open-access weights, and can be run in non-research grade GPUs. We extensively validated RAVEN on in-vivo and ex-vivo datasets acquired at a wide range of voxel sizes and modalities. Furthermore, we assessed the downstream quality of segmenting the upsampled MRIs, demonstrating that RAVEN achieves state-of-the-art performance, significantly improving over other tested super-resolution methods.

## Supplementary Material

Supplementary Material

## Data Availability

All the datasets used in this study (except for DBCBB) are available at the following URLs: ADNI: https://adni.loni.usc.edu/data-samples/adni-data/neuroimaging/mri/mri-image-data-sets/; AHEAD: https://uvaauas.figshare.com/articles/dataset/The_Amsterdam_Ultra-high_field_adult_lifespan_database_AHEAD_A_freely_available_multimodal_7_Tesla_submillimeter_magnetic_resonance_imaging_database/10007840; AOMIC: https://nilab-uva.github.io/AOMIC.github.io/; CHEN: https://springernature.figshare.com/articles/dataset/UNC_Paired_3T-7T_Dataset/23706033; HBA: https://osf.io/ckh5t/files/osfstorage; HCP: https://www.humanconnectome.org/; OSCBS: https://openscience.cbs.mpg.de/bazin/7T_Quantitative/; LUSEBRINK: https://openneuro.org/datasets/ds003563/versions/1.1.0; The code and trained weights are publicly available on https://github.com/waadgo/raven.
