# Supplementary material for: RAVEN: Robust, generalizable, multi-resolution structural MRI upsampling using autoencoders
Source: Imaging Neurosci (Camb). 2026 Aug 4;4:IMAG.a.1326. doi: 10.1162/IMAG.a.1326 (PMC13440154; doi:10.1162/IMAG.a.1326)
Supplement: Supplementary file 1 [file IMAG.a.1326_supp.pdf]

Supplementary Materials

Data Description:

Amsterdam Ultra-high field adult lifespan database (AHEAD): AHEAD (Alkemade et al., 2020) was designed to characterize healthy adult lifespan variation at ultra-high field with submillimeter, quantitative 7T MRI optimized for subcortical anatomy.

Amsterdam Open MRI Collection (AOMIC): Comprises three main datasets (Snoek et al., 2021). **ID1000** is part of the Amsterdam Open MRI Collection and was designed as a large normative 3T cohort to benchmark methods and study variability in brain–behavior relationships. **PIOP1** targets task-based individual-differences mapping in young adults to support robust, reproducible cognitive-neuroimaging pipelines. **PIOP2** complements PIOP1 with additional tasks and phenotypes to facilitate replication and modeling of brain–behavior links under harmonized 3 T acquisitions.

CHEN: This paired 3T/7T T1w/T2w dataset was created to enable development and evaluation of 3T to 7T image synthesis methods using matched contrasts within subjects (X. Chen et al., 2023).

DBCBB: Developed to standardize post-mortem MRI acquisition in a large brain bank and to link MRI with histology for neurodegenerative disease research (Dadar et al., 2024).

Table S1: Characteristics of the specimens used from the DBCBB cohort

Diagnosis	Training (n=103)	Validation (n=34)	Test (n=22)	Ground Truth LR→HR (n=23)
Alzheimer's disease	38	13	8	5
Parkinson's disease	9	4	3	1
Amyotrophic lateral sclerosis (ALS)	3	2	1	6
Frontotemporal dementia	6	1	2	0
Lewy body dementia	4	2	1	0
Vascular dementia / disease	4	4	1	0
Mild cognitive impairment	4	0	0	2
Mixed dementia	3	0	0	0
Corticobasal degeneration	2	0	0	0
Progressive supranuclear palsy	2	0	0	0
Dementia (unspecified)	2	1	2	0
Cognitively normal (control)	6	1	1	0
Bipolar disorder	1	1	0	0
ADHD	1	0	0	0
Hippocampal sclerosis	1	0	0	0
Multiple sclerosis	1	0	0	0
Brain tumor	0	0	1	0
Unknown	16	5	3	9

Human Brain Atlas (HBA): HBA was designed to provide ultra-detailed in-vivo MRI with expert segmentations for fine-scale neuroanatomy, and method validation at very high spatial resolution (Schira et al., 2023).

Human Connectome Project (HCP): HCP established high-quality 3T MRIs in healthy young adults to map structural and functional connectivity and serve as a community benchmark for pipelines/analyses.

Open Science CBS Neuroimaging Repository (OSCBS): OSCBS was created to disseminate ultra-high-field (7T) quantitative/anatomical datasets (e.g., MP2RAGE, multi-echo FLASH) for high-resolution method development and validation (Tardif et al., 2016).

LUSEBRINK: Proposed as an ultra-high-resolution MRI “human phantom”, this dataset was designed to test feasibility of fine-scale morphometry and algorithm testing with 7T in-vivo anatomy (Lüsebrink et al., 2017).

Table S2: Performance of tested methods per degradation type and upsampling factor (scale). Metrics reported as "mean ± (standard deviation)".

Degradation	Scale	Upsampling Method	LPIPS ↓	MAE ↓	PSNR ↑	SSIM ↑
Deg. A	2×2×2	ArSSR-RDN	0.188 +/- (0.052)	0.047 +/- (0.029)	31.140 +/- (3.514)	0.823 +/- (0.063)
		ArSSR-ResCNN	0.233 +/- (0.041)	0.054 +/- (0.022)	29.781 +/- (3.324)	0.782 +/- (0.049)
		Lanczos	0.170 +/- (0.040)	0.033 +/- (0.016)	35.721 +/- (3.682)	0.878 +/- (0.046)
		NLMUPSAMPLE	0.179 +/- (0.041)	0.036 +/- (0.018)	34.382 +/- (3.284)	0.873 +/- (0.042)
		RAVEN	0.052 +/- (0.020)	0.030 +/- (0.016)	35.818 +/- (3.250)	0.899 +/- (0.041)
		4× RAVEN	0.078 +/- (0.026)	0.036 +/- (0.013)	34.015 +/- (2.594)	0.878 +/- (0.039)
		Augmented RAVEN	0.066 +/- (0.027)	0.035 +/- (0.017)	34.385 +/- (3.039)	0.882 +/- (0.041)
		In-vivo RAVEN	0.058 +/- (0.021)	0.036 +/- (0.015)	34.097 +/- (3.008)	0.884 +/- (0.038)
		L1 RAVEN	0.070 +/- (0.029)	0.031 +/- (0.016)	35.790 +/- (3.489)	0.900 +/- (0.039)
		VGG-16 RAVEN	0.057 +/- (0.021)	0.040 +/- (0.013)	32.777 +/- (2.898)	0.883 +/- (0.039)
		SuperFormer	0.147 +/- (0.025)	0.058 +/- (0.029)	29.326 +/- (3.685)	0.826 +/- (0.047)
		Trilinear	0.129 +/- (0.022)	0.039 +/- (0.016)	33.876 +/- (3.165)	0.851 +/- (0.038)
	3×3×3	ArSSR-RDN	0.281 +/- (0.048)	0.073 +/- (0.024)	27.236 +/- (2.723)	0.663 +/- (0.057)
		ArSSR-ResCNN	0.334 +/- (0.046)	0.080 +/- (0.026)	26.363 +/- (2.890)	0.621 +/- (0.054)
		Lanczos	0.353 +/- (0.050)	0.056 +/- (0.022)	30.291 +/- (3.020)	0.741 +/- (0.051)
		NLMUPSAMPLE	0.293 +/- (0.046)	0.054 +/- (0.021)	30.461 +/- (2.947)	0.751 +/- (0.046)
		RAVEN	0.148 +/- (0.039)	0.044 +/- (0.022)	32.144 +/- (3.291)	0.831 +/- (0.047)
		4× RAVEN	0.150 +/- (0.039)	0.048 +/- (0.021)	31.023 +/- (2.744)	0.812 +/- (0.046)
		Augmented RAVEN	0.150 +/- (0.045)	0.047 +/- (0.023)	31.461 +/- (3.192)	0.822 +/- (0.048)
		In-vivo RAVEN	0.146 +/- (0.043)	0.047 +/- (0.023)	31.529 +/- (3.210)	0.828 +/- (0.047)
		L1 RAVEN	0.205 +/- (0.048)	0.046 +/- (0.023)	31.699 +/- (3.265)	0.809 +/- (0.046)
		VGG-16 RAVEN	0.139 +/- (0.039)	0.048 +/- (0.020)	31.141 +/- (3.073)	0.839 +/- (0.045)
		SuperFormer	0.339 +/- (0.038)	0.080 +/- (0.025)	26.243 +/- (2.818)	0.663 +/- (0.046)
		Trilinear	0.332 +/- (0.041)	0.067 +/- (0.023)	28.261 +/- (2.676)	0.681 +/- (0.048)
	4×4×4	ArSSR-RDN	0.352 +/- (0.058)	0.087 +/- (0.027)	26.162 +/- (2.523)	0.556 +/- (0.069)
		ArSSR-ResCNN	0.373 +/- (0.054)	0.086 +/- (0.027)	26.235 +/- (2.644)	0.564 +/- (0.069)
		Lanczos	0.501 +/- (0.044)	0.102 +/- (0.026)	24.842 +/- (2.220)	0.465 +/- (0.063)
		NLMUPSAMPLE	0.492 +/- (0.046)	0.104 +/- (0.028)	24.736 +/- (2.271)	0.460 +/- (0.068)
		RAVEN	0.262 +/- (0.030)	0.103 +/- (0.027)	24.729 +/- (2.263)	0.457 +/- (0.065)
		4× RAVEN	0.210 +/- (0.030)	0.102 +/- (0.027)	25.027 +/- (2.252)	0.466 +/- (0.068)
		Augmented RAVEN	0.247 +/- (0.033)	0.103 +/- (0.028)	24.783 +/- (2.281)	0.455 +/- (0.064)
		In-vivo RAVEN	0.236 +/- (0.034)	0.104 +/- (0.028)	24.848 +/- (2.306)	0.456 +/- (0.066)
		L1 RAVEN	0.328 +/- (0.037)	0.102 +/- (0.027)	24.965 +/- (2.232)	0.465 +/- (0.064)
		VGG-16 RAVEN	0.252 +/- (0.027)	0.102 +/- (0.026)	24.954 +/- (2.354)	0.460 +/- (0.063)
		SuperFormer	0.492 +/- (0.041)	0.110 +/- (0.026)	23.818 +/- (2.056)	0.433 +/- (0.058)
		Trilinear	0.495 +/- (0.042)	0.109 +/- (0.026)	24.046 +/- (2.064)	0.434 +/- (0.059)
Deg. B	2×2×2	ArSSR-RDN	0.226 +/- (0.045)	0.053 +/- (0.022)	29.836 +/- (3.368)	0.804 +/- (0.052)
		ArSSR-ResCNN	0.272 +/- (0.045)	0.060 +/- (0.024)	28.822 +/- (3.281)	0.758 +/- (0.050)
		Lanczos	0.212 +/- (0.043)	0.038 +/- (0.018)	33.732 +/- (3.353)	0.861 +/- (0.043)
		NLMUPSAMPLE	0.240 +/- (0.059)	0.045 +/- (0.034)	32.323 +/- (3.450)	0.832 +/- (0.058)
		RAVEN	0.061 +/- (0.024)	0.030 +/- (0.018)	35.588 +/- (3.453)	0.907 +/- (0.039)
		4× RAVEN	0.085 +/- (0.030)	0.036 +/- (0.016)	33.633 +/- (2.758)	0.881 +/- (0.038)
		Augmented RAVEN	0.074 +/- (0.034)	0.035 +/- (0.019)	34.159 +/- (3.302)	0.890 +/- (0.041)
		In-vivo RAVEN	0.063 +/- (0.027)	0.035 +/- (0.017)	34.098 +/- (3.230)	0.896 +/- (0.037)
		L1 RAVEN	0.087 +/- (0.037)	0.031 +/- (0.019)	35.486 +/- (3.749)	0.906 +/- (0.039)
		VGG-16 RAVEN	0.063 +/- (0.026)	0.039 +/- (0.015)	32.836 +/- (3.091)	0.897 +/- (0.037)
		SuperFormer	0.203 +/- (0.031)	0.063 +/- (0.026)	28.538 +/- (3.411)	0.792 +/- (0.044)
		Trilinear	0.185 +/- (0.033)	0.047 +/- (0.020)	31.756 +/- (3.125)	0.814 +/- (0.041)
	3×3×3	ArSSR-RDN	0.344 +/- (0.045)	0.078 +/- (0.026)	26.714 +/- (2.622)	0.624 +/- (0.056)
		ArSSR-ResCNN	0.408 +/- (0.044)	0.085 +/- (0.026)	25.988 +/- (2.611)	0.579 +/- (0.055)
		Lanczos	0.416 +/- (0.051)	0.068 +/- (0.023)	28.185 +/- (2.689)	0.681 +/- (0.049)
		NLMUPSAMPLE	0.387 +/- (0.049)	0.068 +/- (0.023)	28.064 +/- (2.654)	0.675 +/- (0.049)

		RAVEN	0.206 +/- (0.043)	0.055 +/- (0.024)	29.946 +/- (3.072)	0.771 +/- (0.051)
		4× RAVEN	0.180 +/- (0.042)	0.055 +/- (0.023)	29.692 +/- (2.711)	0.775 +/- (0.049)
		Augmented RAVEN	0.196 +/- (0.047)	0.055 +/- (0.025)	29.791 +/- (3.036)	0.773 +/- (0.051)
		In-vivo RAVEN	0.187 +/- (0.047)	0.054 +/- (0.024)	30.071 +/- (3.047)	0.783 +/- (0.051)
		L1 RAVEN	0.289 +/- (0.049)	0.059 +/- (0.024)	29.277 +/- (2.875)	0.733 +/- (0.049)
		VGG-16 RAVEN	0.182 +/- (0.042)	0.055 +/- (0.022)	29.762 +/- (3.007)	0.795 +/- (0.046)
		SuperFormer	0.424 +/- (0.042)	0.092 +/- (0.026)	24.893 +/- (2.617)	0.597 +/- (0.048)
		Trilinear	0.422 +/- (0.046)	0.079 +/- (0.023)	26.620 +/- (2.422)	0.615 +/- (0.050)
	4×4×4	ArSSR-RDN	0.471 +/- (0.037)	0.100 +/- (0.026)	24.557 +/- (2.229)	0.486 +/- (0.059)
		ArSSR-ResCNN	0.497 +/- (0.034)	0.101 +/- (0.028)	24.427 +/- (2.456)	0.487 +/- (0.062)
		Lanczos	0.559 +/- (0.042)	0.110 +/- (0.025)	23.903 +/- (2.031)	0.432 +/- (0.059)
		NLMUPSAMPLE	0.562 +/- (0.046)	0.114 +/- (0.036)	23.611 +/- (2.392)	0.420 +/- (0.072)
		RAVEN	0.373 +/- (0.033)	0.110 +/- (0.027)	23.792 +/- (2.173)	0.428 +/- (0.059)
		4× RAVEN	0.272 +/- (0.032)	0.107 +/- (0.028)	24.281 +/- (2.210)	0.451 +/- (0.062)
		Augmented RAVEN	0.339 +/- (0.037)	0.110 +/- (0.028)	23.889 +/- (2.201)	0.426 +/- (0.058)
		In-vivo RAVEN	0.321 +/- (0.035)	0.109 +/- (0.028)	24.089 +/- (2.196)	0.430 +/- (0.060)
		L1 RAVEN	0.471 +/- (0.035)	0.110 +/- (0.027)	23.875 +/- (2.101)	0.428 +/- (0.058)
		VGG-16 RAVEN	0.334 +/- (0.031)	0.109 +/- (0.026)	23.889 +/- (2.209)	0.433 +/- (0.057)
		SuperFormer	0.570 +/- (0.038)	0.119 +/- (0.025)	22.932 +/- (1.916)	0.398 +/- (0.054)
		Trilinear	0.575 +/- (0.039)	0.117 +/- (0.025)	23.140 +/- (1.944)	0.399 +/- (0.056)
Deg. C	2×2×2	ArSSR-RDN	0.190 +/- (0.046)	0.046 +/- (0.018)	31.408 +/- (3.096)	0.823 +/- (0.051)
		ArSSR-ResCNN	0.227 +/- (0.045)	0.051 +/- (0.021)	30.186 +/- (3.207)	0.791 +/- (0.048)
		Lanczos	0.168 +/- (0.046)	0.032 +/- (0.016)	35.059 +/- (3.548)	0.886 +/- (0.044)
		NLMUPSAMPLE	0.185 +/- (0.047)	0.034 +/- (0.016)	34.865 +/- (3.317)	0.877 +/- (0.042)
		RAVEN	0.063 +/- (0.027)	0.030 +/- (0.015)	35.444 +/- (3.243)	0.901 +/- (0.039)
		4× RAVEN	0.086 +/- (0.033)	0.035 +/- (0.014)	34.097 +/- (2.765)	0.883 +/- (0.037)
		Augmented RAVEN	0.079 +/- (0.035)	0.034 +/- (0.016)	34.492 +/- (2.883)	0.885 +/- (0.038)
		In-vivo RAVEN	0.066 +/- (0.027)	0.035 +/- (0.014)	33.853 +/- (2.869)	0.884 +/- (0.036)
		L1 RAVEN	0.082 +/- (0.035)	0.030 +/- (0.016)	35.571 +/- (3.423)	0.903 +/- (0.038)
		VGG-16 RAVEN	0.074 +/- (0.029)	0.041 +/- (0.012)	32.131 +/- (2.535)	0.880 +/- (0.037)
		SuperFormer	0.137 +/- (0.027)	0.056 +/- (0.023)	29.409 +/- (3.275)	0.829 +/- (0.041)
		Trilinear	0.122 +/- (0.029)	0.040 +/- (0.017)	33.424 +/- (3.142)	0.845 +/- (0.043)
	3×3×3	ArSSR-RDN	0.272 +/- (0.049)	0.069 +/- (0.022)	27.851 +/- (2.509)	0.658 +/- (0.054)
		ArSSR-ResCNN	0.304 +/- (0.046)	0.073 +/- (0.024)	27.252 +/- (2.743)	0.638 +/- (0.052)
		Lanczos	0.346 +/- (0.054)	0.051 +/- (0.021)	30.924 +/- (3.094)	0.762 +/- (0.053)
		NLMUPSAMPLE	0.276 +/- (0.050)	0.049 +/- (0.018)	31.356 +/- (2.865)	0.765 +/- (0.048)
		RAVEN	0.177 +/- (0.041)	0.045 +/- (0.019)	32.021 +/- (2.914)	0.797 +/- (0.049)
		4× RAVEN	0.174 +/- (0.041)	0.048 +/- (0.018)	31.253 +/- (2.556)	0.788 +/- (0.047)
		Augmented RAVEN	0.179 +/- (0.045)	0.047 +/- (0.020)	31.494 +/- (2.755)	0.784 +/- (0.048)
		In-vivo RAVEN	0.168 +/- (0.042)	0.047 +/- (0.020)	31.528 +/- (2.848)	0.793 +/- (0.047)
		L1 RAVEN	0.211 +/- (0.048)	0.046 +/- (0.020)	32.007 +/- (3.019)	0.794 +/- (0.048)
		VGG-16 RAVEN	0.181 +/- (0.041)	0.052 +/- (0.016)	30.022 +/- (2.338)	0.784 +/- (0.049)
		SuperFormer	0.308 +/- (0.039)	0.073 +/- (0.023)	27.132 +/- (2.719)	0.690 +/- (0.047)
		Trilinear	0.303 +/- (0.044)	0.060 +/- (0.021)	29.355 +/- (2.757)	0.705 +/- (0.050)
	4×4×4	ArSSR-RDN	0.323 +/- (0.065)	0.106 +/- (0.025)	24.369 +/- (2.155)	0.430 +/- (0.051)
		ArSSR-ResCNN	0.337 +/- (0.065)	0.105 +/- (0.026)	24.429 +/- (2.255)	0.436 +/- (0.051)
		Lanczos	0.448 +/- (0.054)	0.066 +/- (0.026)	28.340 +/- (2.984)	0.655 +/- (0.060)
		NLMUPSAMPLE	0.431 +/- (0.055)	0.067 +/- (0.026)	28.345 +/- (2.984)	0.649 +/- (0.067)
		RAVEN	0.227 +/- (0.041)	0.070 +/- (0.032)	27.858 +/- (3.198)	0.643 +/- (0.061)
		4× RAVEN	0.194 +/- (0.054)	0.067 +/- (0.039)	28.392 +/- (3.073)	0.664 +/- (0.066)
		Augmented RAVEN	0.217 +/- (0.038)	0.072 +/- (0.031)	27.678 +/- (3.001)	0.627 +/- (0.058)
		In-vivo RAVEN	0.209 +/- (0.038)	0.072 +/- (0.032)	27.686 +/- (3.065)	0.638 +/- (0.058)
		L1 RAVEN	0.269 +/- (0.051)	0.072 +/- (0.032)	27.588 +/- (3.600)	0.652 +/- (0.060)
		VGG-16 RAVEN	0.269 +/- (0.046)	0.078 +/- (0.025)	26.651 +/- (2.463)	0.597 +/- (0.061)
		SuperFormer	0.425 +/- (0.046)	0.083 +/- (0.022)	26.077 +/- (2.359)	0.589 +/- (0.055)

		Trilinear	0.418 +/- (0.049)	0.078 +/- (0.024)	26.850 +/- (2.559)	0.591 +/- (0.056)

Table S3: Performance of tested methods per dataset, degradation type and upsampling factor (scale). Metrics reported as "mean ± (standard deviation)".

Dataset	Degradation	Scale	Upsampling Method	LPIPS ↓	MAE ↓	PSNR ↑	SSIM ↑
AHEAD	Deg. A	2×2×2	ArSSR-RDN	0.302 +/- (0.023)	0.094 +/- (0.037)	25.317 +/- (3.783)	0.694 +/- (0.039)
			ArSSR-ResCNN	0.353 +/- (0.019)	0.105 +/- (0.045)	24.767 +/- (4.056)	0.662 +/- (0.031)
			Lanczos	0.256 +/- (0.019)	0.093 +/- (0.026)	23.621 +/- (3.321)	0.719 +/- (0.041)
			NLMUPSAMPLE	0.282 +/- (0.021)	0.090 +/- (0.031)	24.808 +/- (3.632)	0.743 +/- (0.028)
			RAVEN	0.120 +/- (0.017)	0.076 +/- (0.035)	28.548 +/- (4.967)	0.751 +/- (0.032)
			4× RAVEN	0.157 +/- (0.019)	0.075 +/- (0.026)	27.742 +/- (4.087)	0.742 +/- (0.026)
			Augmented RAVEN	0.163 +/- (0.023)	0.087 +/- (0.045)	26.873 +/- (6.022)	0.733 +/- (0.040)
			In-vivo RAVEN	0.131 +/- (0.020)	0.079 +/- (0.032)	27.245 +/- (4.201)	0.747 +/- (0.034)
			L1 RAVEN	0.172 +/- (0.025)	0.080 +/- (0.033)	26.625 +/- (4.470)	0.759 +/- (0.039)
			VGG-16 RAVEN	0.129 +/- (0.019)	0.079 +/- (0.026)	26.650 +/- (3.902)	0.745 +/- (0.033)
			SuperFormer	0.228 +/- (0.054)	0.120 +/- (0.083)	22.363 +/- (5.763)	0.679 +/- (0.104)
			Trilinear	0.205 +/- (0.015)	0.094 +/- (0.032)	24.559 +/- (3.599)	0.727 +/- (0.027)
		3×3×3	ArSSR-RDN	0.410 +/- (0.021)	0.123 +/- (0.056)	23.331 +/- (4.586)	0.557 +/- (0.042)
			ArSSR-ResCNN	0.461 +/- (0.019)	0.129 +/- (0.053)	22.836 +/- (4.172)	0.529 +/- (0.035)
			Lanczos	0.494 +/- (0.032)	0.130 +/- (0.051)	21.081 +/- (3.930)	0.593 +/- (0.045)
			NLMUPSAMPLE	0.433 +/- (0.030)	0.123 +/- (0.050)	21.926 +/- (4.008)	0.619 +/- (0.039)
			RAVEN	0.264 +/- (0.036)	0.110 +/- (0.056)	23.799 +/- (5.173)	0.678 +/- (0.048)
			4× RAVEN	0.268 +/- (0.034)	0.111 +/- (0.057)	23.738 +/- (5.276)	0.667 +/- (0.050)
			Augmented RAVEN	0.303 +/- (0.038)	0.113 +/- (0.063)	23.681 +/- (5.829)	0.669 +/- (0.057)
			In-vivo RAVEN	0.289 +/- (0.039)	0.109 +/- (0.057)	23.989 +/- (4.987)	0.682 +/- (0.051)
			L1 RAVEN	0.359 +/- (0.041)	0.113 +/- (0.055)	23.063 +/- (4.708)	0.665 +/- (0.051)
			VGG-16 RAVEN	0.262 +/- (0.039)	0.110 +/- (0.052)	23.192 +/- (4.703)	0.687 +/- (0.053)
			SuperFormer	0.456 +/- (0.026)	0.131 +/- (0.048)	21.286 +/- (3.999)	0.557 +/- (0.037)
			Trilinear	0.463 +/- (0.035)	0.134 +/- (0.056)	21.325 +/- (4.388)	0.560 +/- (0.040)
		4×4×4	ArSSR-RDN	0.472 +/- (0.041)	0.155 +/- (0.057)	21.083 +/- (3.996)	0.372 +/- (0.030)
			ArSSR-ResCNN	0.495 +/- (0.046)	0.151 +/- (0.055)	21.310 +/- (3.788)	0.388 +/- (0.031)
			Lanczos	0.630 +/- (0.038)	0.155 +/- (0.062)	20.225 +/- (3.852)	0.409 +/- (0.139)
			NLMUPSAMPLE	0.620 +/- (0.037)	0.153 +/- (0.062)	20.500 +/- (3.909)	0.411 +/- (0.135)
			RAVEN	0.338 +/- (0.041)	0.149 +/- (0.069)	21.228 +/- (4.618)	0.422 +/- (0.162)
			4× RAVEN	0.299 +/- (0.054)	0.142 +/- (0.071)	21.979 +/- (5.162)	0.452 +/- (0.188)
			Augmented RAVEN	0.346 +/- (0.054)	0.148 +/- (0.074)	21.538 +/- (5.225)	0.430 +/- (0.166)
			In-vivo RAVEN	0.325 +/- (0.050)	0.144 +/- (0.070)	21.802 +/- (4.814)	0.434 +/- (0.173)
			L1 RAVEN	0.435 +/- (0.051)	0.148 +/- (0.066)	21.027 +/- (4.288)	0.428 +/- (0.158)
			VGG-16 RAVEN	0.322 +/- (0.053)	0.151 +/- (0.060)	20.566 +/- (3.712)	0.424 +/- (0.158)
			SuperFormer	0.612 +/- (0.041)	0.154 +/- (0.062)	20.533 +/- (4.077)	0.385 +/- (0.115)
			Trilinear	0.622 +/- (0.048)	0.153 +/- (0.062)	20.723 +/- (4.195)	0.385 +/- (0.115)
	Deg. B	2×2×2	ArSSR-RDN	0.363 +/- (0.024)	0.111 +/- (0.045)	23.274 +/- (3.999)	0.674 +/- (0.039)
			ArSSR-ResCNN	0.411 +/- (0.020)	0.120 +/- (0.048)	22.755 +/- (3.921)	0.635 +/- (0.033)
			Lanczos	0.329 +/- (0.023)	0.104 +/- (0.038)	22.994 +/- (3.705)	0.715 +/- (0.039)
			NLMUPSAMPLE	0.372 +/- (0.026)	0.109 +/- (0.044)	22.946 +/- (3.996)	0.698 +/- (0.034)
			RAVEN	0.142 +/- (0.023)	0.082 +/- (0.043)	27.457 +/- (5.418)	0.764 +/- (0.036)
			4× RAVEN	0.180 +/- (0.024)	0.085 +/- (0.037)	26.316 +/- (4.766)	0.749 +/- (0.032)
			Augmented RAVEN	0.195 +/- (0.030)	0.093 +/- (0.053)	26.093 +/- (6.327)	0.744 +/- (0.046)
			In-vivo RAVEN	0.159 +/- (0.028)	0.086 +/- (0.042)	26.391 +/- (4.732)	0.762 +/- (0.040)
			L1 RAVEN	0.221 +/- (0.034)	0.090 +/- (0.042)	25.427 +/- (4.724)	0.761 +/- (0.045)
			VGG-16 RAVEN	0.153 +/- (0.027)	0.086 +/- (0.036)	25.740 +/- (4.472)	0.761 +/- (0.039)
			SuperFormer	0.312 +/- (0.036)	0.122 +/- (0.060)	21.600 +/- (4.418)	0.660 +/- (0.076)
			Trilinear	0.303 +/- (0.022)	0.114 +/- (0.045)	22.577 +/- (4.002)	0.681 +/- (0.035)
		3×3×3	ArSSR-RDN	0.471 +/- (0.023)	0.134 +/- (0.061)	22.055 +/- (4.706)	0.521 +/- (0.042)
			ArSSR-ResCNN	0.534 +/- (0.018)	0.139 +/- (0.057)	21.565 +/- (4.211)	0.488 +/- (0.037)
			Lanczos	0.566 +/- (0.034)	0.135 +/- (0.058)	21.214 +/- (4.432)	0.557 +/- (0.041)

			NLMUPSAMPLE	0.536 +/- (0.033)	0.134 +/- (0.058)	21.333 +/- (4.472)	0.558 +/- (0.041)
			RAVEN	0.341 +/- (0.038)	0.125 +/- (0.061)	22.318 +/- (4.970)	0.620 +/- (0.050)
			4× RAVEN	0.310 +/- (0.040)	0.121 +/- (0.063)	22.812 +/- (5.310)	0.632 +/- (0.055)
			Augmented RAVEN	0.359 +/- (0.043)	0.125 +/- (0.067)	22.556 +/- (5.525)	0.626 +/- (0.060)
			In-vivo RAVEN	0.340 +/- (0.042)	0.120 +/- (0.061)	22.987 +/- (4.908)	0.640 +/- (0.053)
			L1 RAVEN	0.447 +/- (0.046)	0.127 +/- (0.060)	21.950 +/- (4.749)	0.600 +/- (0.049)
			VGG-16 RAVEN	0.322 +/- (0.041)	0.125 +/- (0.058)	21.774 +/- (4.510)	0.645 +/- (0.057)
			SuperFormer	0.551 +/- (0.031)	0.138 +/- (0.054)	21.122 +/- (4.268)	0.508 +/- (0.039)
			Trilinear	0.562 +/- (0.039)	0.138 +/- (0.059)	21.323 +/- (4.595)	0.513 +/- (0.040)
		4×4×4	ArSSR-RDN	0.540 +/- (0.019)	0.160 +/- (0.062)	20.569 +/- (4.495)	0.352 +/- (0.020)
			ArSSR-ResCNN	0.570 +/- (0.018)	0.161 +/- (0.058)	20.204 +/- (4.030)	0.355 +/- (0.023)
			Lanczos	0.680 +/- (0.034)	0.153 +/- (0.062)	20.760 +/- (4.192)	0.384 +/- (0.109)
			NLMUPSAMPLE	0.676 +/- (0.033)	0.153 +/- (0.062)	20.781 +/- (4.173)	0.379 +/- (0.105)
			RAVEN	0.444 +/- (0.033)	0.154 +/- (0.069)	21.007 +/- (4.726)	0.382 +/- (0.121)
			4× RAVEN	0.357 +/- (0.057)	0.152 +/- (0.076)	21.239 +/- (5.184)	0.421 +/- (0.158)
			Augmented RAVEN	0.437 +/- (0.048)	0.157 +/- (0.074)	20.794 +/- (5.025)	0.389 +/- (0.126)
			In-vivo RAVEN	0.398 +/- (0.035)	0.153 +/- (0.069)	21.012 +/- (4.559)	0.391 +/- (0.129)
			L1 RAVEN	0.572 +/- (0.040)	0.154 +/- (0.065)	20.720 +/- (4.338)	0.382 +/- (0.114)
			VGG-16 RAVEN	0.408 +/- (0.036)	0.161 +/- (0.063)	19.985 +/- (3.974)	0.381 +/- (0.118)
			SuperFormer	0.675 +/- (0.037)	0.155 +/- (0.059)	20.606 +/- (3.953)	0.355 +/- (0.090)
			Trilinear	0.688 +/- (0.042)	0.154 +/- (0.060)	20.731 +/- (4.045)	0.355 +/- (0.090)
	Deg. C	2×2×2	ArSSR-RDN	0.325 +/- (0.024)	0.099 +/- (0.033)	24.114 +/- (3.298)	0.691 +/- (0.036)
			ArSSR-ResCNN	0.366 +/- (0.021)	0.107 +/- (0.038)	23.894 +/- (3.520)	0.666 +/- (0.030)
			Lanczos	0.282 +/- (0.021)	0.094 +/- (0.024)	23.429 +/- (2.970)	0.733 +/- (0.033)
			NLMUPSAMPLE	0.315 +/- (0.023)	0.093 +/- (0.028)	24.007 +/- (3.302)	0.736 +/- (0.031)
			RAVEN	0.157 +/- (0.024)	0.081 +/- (0.024)	25.559 +/- (3.272)	0.758 +/- (0.033)
			4× RAVEN	0.192 +/- (0.026)	0.082 +/- (0.026)	25.798 +/- (3.149)	0.749 +/- (0.031)
			Augmented RAVEN	0.202 +/- (0.029)	0.085 +/- (0.035)	25.661 +/- (4.245)	0.745 +/- (0.039)
			In-vivo RAVEN	0.161 +/- (0.029)	0.081 +/- (0.025)	25.766 +/- (2.896)	0.752 +/- (0.033)
			L1 RAVEN	0.204 +/- (0.029)	0.086 +/- (0.027)	24.782 +/- (3.232)	0.762 +/- (0.038)
			VGG-16 RAVEN	0.178 +/- (0.024)	0.083 +/- (0.020)	25.051 +/- (2.712)	0.744 +/- (0.033)
			SuperFormer	0.236 +/- (0.022)	0.100 +/- (0.038)	23.397 +/- (4.161)	0.696 +/- (0.045)
			Trilinear	0.228 +/- (0.018)	0.103 +/- (0.030)	23.063 +/- (3.288)	0.700 +/- (0.031)
		3×3×3	ArSSR-RDN	0.399 +/- (0.019)	0.117 +/- (0.047)	23.727 +/- (3.828)	0.551 +/- (0.041)
			ArSSR-ResCNN	0.430 +/- (0.018)	0.121 +/- (0.046)	23.459 +/- (3.618)	0.537 +/- (0.035)
			Lanczos	0.485 +/- (0.026)	0.123 +/- (0.037)	21.244 +/- (3.090)	0.600 +/- (0.039)
			NLMUPSAMPLE	0.416 +/- (0.024)	0.112 +/- (0.033)	22.368 +/- (3.092)	0.623 +/- (0.034)
			RAVEN	0.298 +/- (0.032)	0.106 +/- (0.041)	23.434 +/- (3.809)	0.646 +/- (0.041)
			4× RAVEN	0.299 +/- (0.036)	0.108 +/- (0.044)	23.546 +/- (3.898)	0.640 +/- (0.044)
			Augmented RAVEN	0.326 +/- (0.032)	0.108 +/- (0.047)	23.448 +/- (4.168)	0.634 +/- (0.047)
			In-vivo RAVEN	0.301 +/- (0.038)	0.105 +/- (0.044)	23.771 +/- (3.733)	0.645 +/- (0.046)
			L1 RAVEN	0.356 +/- (0.035)	0.108 +/- (0.041)	23.286 +/- (3.583)	0.646 +/- (0.044)
			VGG-16 RAVEN	0.304 +/- (0.031)	0.108 +/- (0.032)	22.799 +/- (2.954)	0.632 +/- (0.039)
			SuperFormer	0.427 +/- (0.020)	0.123 +/- (0.036)	21.639 +/- (3.478)	0.570 +/- (0.033)
			Trilinear	0.433 +/- (0.027)	0.129 +/- (0.046)	21.368 +/- (3.735)	0.570 +/- (0.038)
		4×4×4	ArSSR-RDN	0.489 +/- (0.032)	0.161 +/- (0.046)	19.916 +/- (2.960)	0.350 +/- (0.037)
			ArSSR-ResCNN	0.505 +/- (0.034)	0.153 +/- (0.052)	20.902 +/- (3.623)	0.366 +/- (0.022)
			Lanczos	0.596 +/- (0.031)	0.150 +/- (0.053)	19.549 +/- (3.448)	0.499 +/- (0.059)
			NLMUPSAMPLE	0.585 +/- (0.032)	0.148 +/- (0.054)	19.845 +/- (3.596)	0.504 +/- (0.059)
			RAVEN	0.346 +/- (0.059)	0.168 +/- (0.078)	19.326 +/- (4.906)	0.489 +/- (0.077)
			4× RAVEN	0.320 +/- (0.062)	0.158 +/- (0.077)	20.221 +/- (5.120)	0.507 +/- (0.092)
			Augmented RAVEN	0.340 +/- (0.050)	0.164 +/- (0.079)	19.770 +/- (5.288)	0.482 +/- (0.077)
			In-vivo RAVEN	0.321 +/- (0.056)	0.164 +/- (0.083)	20.000 +/- (5.384)	0.489 +/- (0.081)
			L1 RAVEN	0.419 +/- (0.063)	0.160 +/- (0.074)	19.517 +/- (4.537)	0.500 +/- (0.082)
			VGG-16 RAVEN	0.350 +/- (0.041)	0.145 +/- (0.042)	20.227 +/- (2.775)	0.473 +/- (0.051)

			SuperFormer	0.566 +/- (0.034)	0.133 +/- (0.035)	21.276 +/- (2.597)	0.466 +/- (0.056)
			Trilinear	0.566 +/- (0.036)	0.146 +/- (0.057)	20.417 +/- (4.058)	0.471 +/- (0.052)
AOMIC	Deg. A	2×2×2	ArSSR-RDN	0.193 +/- (0.063)	0.042 +/- (0.039)	32.568 +/- (2.653)	0.825 +/- (0.070)
			ArSSR-ResCNN	0.225 +/- (0.025)	0.045 +/- (0.007)	31.261 +/- (1.446)	0.783 +/- (0.031)
			Lanczos	0.196 +/- (0.025)	0.031 +/- (0.005)	35.445 +/- (1.589)	0.877 +/- (0.026)
			NLMUPSAMPLE	0.195 +/- (0.034)	0.035 +/- (0.017)	33.934 +/- (1.628)	0.867 +/- (0.036)
			RAVEN	0.052 +/- (0.010)	0.028 +/- (0.005)	35.934 +/- (1.730)	0.895 +/- (0.025)
			4× RAVEN	0.086 +/- (0.017)	0.033 +/- (0.006)	34.185 +/- (1.460)	0.876 +/- (0.025)
			Augmented RAVEN	0.066 +/- (0.014)	0.031 +/- (0.006)	34.958 +/- (1.599)	0.881 +/- (0.026)
			In-vivo RAVEN	0.055 +/- (0.010)	0.032 +/- (0.005)	34.390 +/- (1.396)	0.882 +/- (0.025)
			L1 RAVEN	0.069 +/- (0.014)	0.028 +/- (0.005)	36.066 +/- (1.687)	0.897 +/- (0.024)
			VGG-16 RAVEN	0.056 +/- (0.011)	0.039 +/- (0.005)	32.125 +/- (0.991)	0.879 +/- (0.026)
			SuperFormer	0.143 +/- (0.015)	0.051 +/- (0.015)	29.884 +/- (2.563)	0.828 +/- (0.029)
			Trilinear	0.125 +/- (0.012)	0.037 +/- (0.005)	33.486 +/- (1.300)	0.847 +/- (0.025)
		3×3×3	ArSSR-RDN	0.265 +/- (0.020)	0.067 +/- (0.006)	27.756 +/- (0.998)	0.663 +/- (0.032)
			ArSSR-ResCNN	0.309 +/- (0.021)	0.072 +/- (0.007)	27.016 +/- (0.992)	0.614 +/- (0.034)
			Lanczos	0.339 +/- (0.027)	0.052 +/- (0.005)	30.049 +/- (1.026)	0.747 +/- (0.030)
			NLMUPSAMPLE	0.277 +/- (0.023)	0.052 +/- (0.005)	29.982 +/- (1.040)	0.747 +/- (0.030)
			RAVEN	0.157 +/- (0.024)	0.040 +/- (0.005)	32.362 +/- (1.203)	0.826 +/- (0.026)
			4× RAVEN	0.167 +/- (0.022)	0.045 +/- (0.006)	31.011 +/- (1.145)	0.801 +/- (0.028)
			Augmented RAVEN	0.161 +/- (0.020)	0.043 +/- (0.006)	31.696 +/- (1.286)	0.810 +/- (0.029)
			In-vivo RAVEN	0.151 +/- (0.021)	0.041 +/- (0.006)	31.836 +/- (1.213)	0.822 +/- (0.027)
			L1 RAVEN	0.205 +/- (0.026)	0.042 +/- (0.005)	31.697 +/- (1.127)	0.801 +/- (0.026)
			VGG-16 RAVEN	0.151 +/- (0.023)	0.045 +/- (0.004)	30.960 +/- (0.892)	0.835 +/- (0.027)
			SuperFormer	0.311 +/- (0.021)	0.074 +/- (0.014)	26.464 +/- (1.712)	0.661 +/- (0.038)
			Trilinear	0.307 +/- (0.020)	0.065 +/- (0.005)	27.808 +/- (0.844)	0.673 +/- (0.030)
		4×4×4	ArSSR-RDN	0.330 +/- (0.018)	0.083 +/- (0.006)	26.067 +/- (0.836)	0.542 +/- (0.028)
			ArSSR-ResCNN	0.351 +/- (0.020)	0.082 +/- (0.006)	26.090 +/- (0.842)	0.550 +/- (0.029)
			Lanczos	0.472 +/- (0.019)	0.099 +/- (0.009)	24.514 +/- (1.009)	0.434 +/- (0.032)
			NLMUPSAMPLE	0.465 +/- (0.034)	0.103 +/- (0.024)	24.300 +/- (1.568)	0.424 +/- (0.054)
			RAVEN	0.249 +/- (0.013)	0.100 +/- (0.010)	24.460 +/- (1.051)	0.425 +/- (0.032)
			4× RAVEN	0.210 +/- (0.015)	0.099 +/- (0.010)	24.673 +/- (1.072)	0.434 +/- (0.030)
			Augmented RAVEN	0.239 +/- (0.013)	0.099 +/- (0.010)	24.624 +/- (1.117)	0.421 +/- (0.030)
			In-vivo RAVEN	0.221 +/- (0.014)	0.100 +/- (0.010)	24.554 +/- (1.083)	0.420 +/- (0.030)
			L1 RAVEN	0.310 +/- (0.013)	0.098 +/- (0.010)	24.710 +/- (1.106)	0.432 +/- (0.033)
			VGG-16 RAVEN	0.239 +/- (0.015)	0.099 +/- (0.009)	24.591 +/- (0.949)	0.426 +/- (0.031)
			SuperFormer	0.460 +/- (0.020)	0.106 +/- (0.008)	23.695 +/- (0.828)	0.403 +/- (0.032)
			Trilinear	0.462 +/- (0.021)	0.105 +/- (0.008)	23.852 +/- (0.880)	0.403 +/- (0.032)
	Deg. B	2×2×2	ArSSR-RDN	0.222 +/- (0.025)	0.045 +/- (0.006)	31.124 +/- (1.246)	0.811 +/- (0.032)
			ArSSR-ResCNN	0.257 +/- (0.025)	0.051 +/- (0.006)	30.021 +/- (1.201)	0.758 +/- (0.033)
			Lanczos	0.228 +/- (0.026)	0.036 +/- (0.005)	33.468 +/- (1.369)	0.860 +/- (0.026)
			NLMUPSAMPLE	0.247 +/- (0.075)	0.045 +/- (0.050)	31.801 +/- (2.583)	0.825 +/- (0.075)
			RAVEN	0.063 +/- (0.014)	0.026 +/- (0.005)	36.128 +/- (1.642)	0.905 +/- (0.023)
			4× RAVEN	0.093 +/- (0.018)	0.033 +/- (0.005)	33.927 +/- (1.408)	0.879 +/- (0.024)
			Augmented RAVEN	0.077 +/- (0.017)	0.030 +/- (0.005)	35.044 +/- (1.591)	0.889 +/- (0.024)
			In-vivo RAVEN	0.062 +/- (0.013)	0.030 +/- (0.005)	34.706 +/- (1.345)	0.896 +/- (0.022)
			L1 RAVEN	0.089 +/- (0.017)	0.027 +/- (0.005)	36.042 +/- (1.659)	0.905 +/- (0.022)
			VGG-16 RAVEN	0.066 +/- (0.015)	0.037 +/- (0.004)	32.338 +/- (0.975)	0.895 +/- (0.022)
			SuperFormer	0.190 +/- (0.017)	0.055 +/- (0.014)	29.104 +/- (2.198)	0.796 +/- (0.032)
			Trilinear	0.173 +/- (0.015)	0.044 +/- (0.005)	31.375 +/- (1.120)	0.812 +/- (0.027)
		3×3×3	ArSSR-RDN	0.318 +/- (0.018)	0.075 +/- (0.006)	26.534 +/- (0.755)	0.619 +/- (0.032)
			ArSSR-ResCNN	0.382 +/- (0.018)	0.082 +/- (0.006)	25.821 +/- (0.794)	0.563 +/- (0.032)
			Lanczos	0.386 +/- (0.024)	0.065 +/- (0.005)	27.707 +/- (0.825)	0.675 +/- (0.031)
			NLMUPSAMPLE	0.356 +/- (0.021)	0.066 +/- (0.005)	27.551 +/- (0.825)	0.664 +/- (0.031)
			RAVEN	0.204 +/- (0.021)	0.050 +/- (0.006)	30.047 +/- (1.014)	0.763 +/- (0.029)

			4× RAVEN	0.195 +/- (0.020)	0.052 +/- (0.006)	29.672 +/- (1.111)	0.757 +/- (0.031)
			Augmented RAVEN	0.200 +/- (0.018)	0.051 +/- (0.006)	29.912 +/- (1.140)	0.756 +/- (0.029)
			In-vivo RAVEN	0.187 +/- (0.020)	0.049 +/- (0.006)	30.273 +/- (1.171)	0.770 +/- (0.028)
			L1 RAVEN	0.278 +/- (0.024)	0.056 +/- (0.005)	29.100 +/- (0.900)	0.718 +/- (0.027)
			VGG-16 RAVEN	0.184 +/- (0.023)	0.051 +/- (0.005)	29.756 +/- (0.776)	0.789 +/- (0.029)
			SuperFormer	0.387 +/- (0.021)	0.084 +/- (0.011)	25.234 +/- (1.219)	0.589 +/- (0.037)
			Trilinear	0.387 +/- (0.021)	0.077 +/- (0.006)	26.198 +/- (0.728)	0.600 +/- (0.031)
		4×4×4	ArSSR-RDN	0.453 +/- (0.015)	0.096 +/- (0.006)	24.461 +/- (0.676)	0.465 +/- (0.030)
			ArSSR-ResCNN	0.481 +/- (0.015)	0.097 +/- (0.006)	24.304 +/- (0.635)	0.463 +/- (0.028)
			Lanczos	0.529 +/- (0.022)	0.106 +/- (0.008)	23.705 +/- (0.843)	0.401 +/- (0.033)
			NLMUPSAMPLE	0.539 +/- (0.047)	0.116 +/- (0.047)	23.153 +/- (2.323)	0.380 +/- (0.074)
			RAVEN	0.350 +/- (0.013)	0.104 +/- (0.009)	23.813 +/- (0.946)	0.399 +/- (0.033)
			4× RAVEN	0.272 +/- (0.014)	0.101 +/- (0.010)	24.252 +/- (0.992)	0.420 +/- (0.032)
			Augmented RAVEN	0.323 +/- (0.012)	0.103 +/- (0.010)	23.975 +/- (0.989)	0.396 +/- (0.031)
			In-vivo RAVEN	0.304 +/- (0.011)	0.103 +/- (0.010)	24.091 +/- (1.025)	0.399 +/- (0.031)
			L1 RAVEN	0.452 +/- (0.012)	0.105 +/- (0.009)	23.830 +/- (0.913)	0.396 +/- (0.032)
			VGG-16 RAVEN	0.316 +/- (0.013)	0.105 +/- (0.009)	23.660 +/- (0.819)	0.402 +/- (0.033)
			SuperFormer	0.540 +/- (0.022)	0.115 +/- (0.007)	22.837 +/- (0.674)	0.369 +/- (0.032)
			Trilinear	0.544 +/- (0.023)	0.112 +/- (0.008)	23.098 +/- (0.745)	0.370 +/- (0.033)
	Deg. C	2×2×2	ArSSR-RDN	0.196 +/- (0.028)	0.038 +/- (0.007)	32.759 +/- (1.560)	0.831 +/- (0.031)
			ArSSR-ResCNN	0.223 +/- (0.026)	0.043 +/- (0.007)	31.604 +/- (1.448)	0.794 +/- (0.030)
			Lanczos	0.195 +/- (0.026)	0.030 +/- (0.005)	34.853 +/- (1.495)	0.890 +/- (0.023)
			NLMUPSAMPLE	0.206 +/- (0.026)	0.032 +/- (0.005)	34.398 +/- (1.502)	0.875 +/- (0.026)
			RAVEN	0.067 +/- (0.013)	0.027 +/- (0.005)	35.813 +/- (1.604)	0.901 +/- (0.022)
			4× RAVEN	0.098 +/- (0.019)	0.032 +/- (0.005)	34.295 +/- (1.465)	0.883 +/- (0.023)
			Augmented RAVEN	0.086 +/- (0.018)	0.030 +/- (0.005)	34.950 +/- (1.497)	0.887 +/- (0.023)
			In-vivo RAVEN	0.068 +/- (0.013)	0.031 +/- (0.005)	34.398 +/- (1.456)	0.887 +/- (0.022)
			L1 RAVEN	0.087 +/- (0.017)	0.027 +/- (0.005)	35.920 +/- (1.634)	0.905 +/- (0.022)
			VGG-16 RAVEN	0.079 +/- (0.015)	0.039 +/- (0.004)	31.897 +/- (0.804)	0.883 +/- (0.022)
			SuperFormer	0.136 +/- (0.016)	0.051 +/- (0.015)	29.837 +/- (2.408)	0.831 +/- (0.028)
			Trilinear	0.119 +/- (0.014)	0.037 +/- (0.005)	33.124 +/- (1.319)	0.846 +/- (0.026)
		3×3×3	ArSSR-RDN	0.258 +/- (0.022)	0.064 +/- (0.008)	28.294 +/- (1.227)	0.666 +/- (0.029)
			ArSSR-ResCNN	0.283 +/- (0.022)	0.066 +/- (0.008)	27.970 +/- (1.280)	0.638 +/- (0.033)
			Lanczos	0.348 +/- (0.029)	0.046 +/- (0.005)	30.892 +/- (1.045)	0.776 +/- (0.027)
			NLMUPSAMPLE	0.270 +/- (0.026)	0.046 +/- (0.006)	31.099 +/- (1.137)	0.771 +/- (0.028)
			RAVEN	0.184 +/- (0.024)	0.041 +/- (0.006)	32.281 +/- (1.268)	0.804 +/- (0.028)
			4× RAVEN	0.194 +/- (0.023)	0.044 +/- (0.006)	31.390 +/- (1.205)	0.789 +/- (0.029)
			Augmented RAVEN	0.187 +/- (0.020)	0.043 +/- (0.006)	31.722 +/- (1.346)	0.788 +/- (0.031)
			In-vivo RAVEN	0.172 +/- (0.021)	0.042 +/- (0.006)	32.076 +/- (1.320)	0.801 +/- (0.029)
			L1 RAVEN	0.211 +/- (0.026)	0.041 +/- (0.006)	32.248 +/- (1.335)	0.799 +/- (0.028)
			VGG-16 RAVEN	0.192 +/- (0.022)	0.050 +/- (0.005)	29.860 +/- (0.766)	0.793 +/- (0.030)
			SuperFormer	0.291 +/- (0.020)	0.066 +/- (0.013)	27.397 +/- (1.763)	0.696 +/- (0.033)
			Trilinear	0.290 +/- (0.021)	0.058 +/- (0.005)	29.007 +/- (0.918)	0.708 +/- (0.029)
		4×4×4	ArSSR-RDN	0.300 +/- (0.019)	0.102 +/- (0.014)	24.352 +/- (1.513)	0.418 +/- (0.033)
			ArSSR-ResCNN	0.315 +/- (0.023)	0.101 +/- (0.014)	24.410 +/- (1.533)	0.423 +/- (0.034)
			Lanczos	0.418 +/- (0.025)	0.063 +/- (0.006)	28.093 +/- (0.832)	0.659 +/- (0.029)
			NLMUPSAMPLE	0.398 +/- (0.028)	0.065 +/- (0.013)	27.917 +/- (1.240)	0.642 +/- (0.064)
			RAVEN	0.217 +/- (0.011)	0.068 +/- (0.006)	27.221 +/- (0.869)	0.640 +/- (0.034)
			4× RAVEN	0.198 +/- (0.014)	0.064 +/- (0.006)	27.853 +/- (0.864)	0.648 +/- (0.031)
			Augmented RAVEN	0.207 +/- (0.013)	0.070 +/- (0.007)	26.971 +/- (0.936)	0.622 +/- (0.032)
			In-vivo RAVEN	0.196 +/- (0.011)	0.070 +/- (0.006)	26.872 +/- (0.920)	0.635 +/- (0.030)
			L1 RAVEN	0.243 +/- (0.015)	0.075 +/- (0.010)	25.870 +/- (1.545)	0.647 +/- (0.031)
			VGG-16 RAVEN	0.253 +/- (0.013)	0.076 +/- (0.008)	26.370 +/- (0.962)	0.588 +/- (0.030)
			SuperFormer	0.398 +/- (0.016)	0.082 +/- (0.007)	25.647 +/- (0.815)	0.585 +/- (0.031)
			Trilinear	0.380 +/- (0.022)	0.075 +/- (0.006)	26.465 +/- (0.760)	0.580 +/- (0.031)

CHEN	Deg. A	2×2×2	ArSSR-RDN	0.193 +/- (0.041)	0.034 +/- (0.012)	34.838 +/- (3.345)	0.832 +/- (0.071)
			ArSSR-ResCNN	0.231 +/- (0.042)	0.041 +/- (0.012)	32.376 +/- (2.581)	0.804 +/- (0.063)
			Lanczos	0.172 +/- (0.037)	0.024 +/- (0.006)	39.005 +/- (2.682)	0.892 +/- (0.021)
			NLMUPSAMPLE	0.186 +/- (0.034)	0.027 +/- (0.006)	36.790 +/- (1.957)	0.886 +/- (0.019)
			RAVEN	0.058 +/- (0.015)	0.024 +/- (0.007)	38.179 +/- (3.417)	0.899 +/- (0.026)
			4× RAVEN	0.083 +/- (0.019)	0.029 +/- (0.007)	36.109 +/- (2.506)	0.882 +/- (0.024)
			Augmented RAVEN	0.072 +/- (0.017)	0.034 +/- (0.008)	33.355 +/- (2.296)	0.881 +/- (0.024)
			In-vivo RAVEN	0.062 +/- (0.015)	0.026 +/- (0.007)	37.478 +/- (3.262)	0.890 +/- (0.027)
			L1 RAVEN	0.087 +/- (0.021)	0.024 +/- (0.006)	37.506 +/- (2.307)	0.904 +/- (0.020)
			VGG-16 RAVEN	0.062 +/- (0.015)	0.028 +/- (0.007)	36.952 +/- (3.517)	0.891 +/- (0.023)
			SuperFormer	0.147 +/- (0.017)	0.051 +/- (0.010)	30.440 +/- (2.225)	0.842 +/- (0.012)
			Trilinear	0.130 +/- (0.020)	0.030 +/- (0.006)	35.986 +/- (1.867)	0.871 +/- (0.021)
		3×3×3	ArSSR-RDN	0.289 +/- (0.052)	0.054 +/- (0.018)	30.668 +/- (3.053)	0.704 +/- (0.090)
			ArSSR-ResCNN	0.333 +/- (0.046)	0.061 +/- (0.017)	28.955 +/- (2.534)	0.672 +/- (0.087)
			Lanczos	0.361 +/- (0.044)	0.042 +/- (0.009)	32.962 +/- (1.892)	0.787 +/- (0.034)
			NLMUPSAMPLE	0.304 +/- (0.039)	0.042 +/- (0.008)	32.765 +/- (1.796)	0.791 +/- (0.032)
			RAVEN	0.181 +/- (0.023)	0.040 +/- (0.014)	33.132 +/- (3.574)	0.830 +/- (0.034)
			4× RAVEN	0.179 +/- (0.017)	0.046 +/- (0.013)	31.406 +/- (2.873)	0.812 +/- (0.037)
			Augmented RAVEN	0.173 +/- (0.025)	0.053 +/- (0.014)	29.388 +/- (2.811)	0.817 +/- (0.037)
			In-vivo RAVEN	0.178 +/- (0.029)	0.042 +/- (0.014)	32.674 +/- (3.032)	0.828 +/- (0.039)
			L1 RAVEN	0.253 +/- (0.026)	0.044 +/- (0.013)	31.777 +/- (2.797)	0.812 +/- (0.036)
			VGG-16 RAVEN	0.163 +/- (0.023)	0.040 +/- (0.007)	32.734 +/- (1.616)	0.846 +/- (0.030)
			SuperFormer	0.351 +/- (0.025)	0.072 +/- (0.002)	27.081 +/- (0.912)	0.705 +/- (0.035)
			Trilinear	0.343 +/- (0.030)	0.054 +/- (0.011)	30.286 +/- (1.779)	0.735 +/- (0.046)
		4×4×4	ArSSR-RDN	0.488 +/- (0.131)	0.100 +/- (0.035)	24.155 +/- (4.314)	0.446 +/- (0.134)
			ArSSR-ResCNN	0.479 +/- (0.102)	0.093 +/- (0.033)	25.034 +/- (4.347)	0.485 +/- (0.126)
			Lanczos	0.498 +/- (0.026)	0.067 +/- (0.012)	28.514 +/- (1.316)	0.639 +/- (0.109)
			NLMUPSAMPLE	0.491 +/- (0.025)	0.069 +/- (0.010)	28.073 +/- (0.979)	0.631 +/- (0.106)
			RAVEN	0.285 +/- (0.014)	0.073 +/- (0.015)	27.450 +/- (2.193)	0.628 +/- (0.111)
			4× RAVEN	0.224 +/- (0.008)	0.068 +/- (0.013)	27.934 +/- (1.759)	0.658 +/- (0.125)
			Augmented RAVEN	0.256 +/- (0.008)	0.082 +/- (0.013)	25.760 +/- (1.607)	0.621 +/- (0.111)
			In-vivo RAVEN	0.260 +/- (0.021)	0.072 +/- (0.015)	27.795 +/- (2.355)	0.633 +/- (0.117)
			L1 RAVEN	0.368 +/- (0.018)	0.073 +/- (0.011)	27.221 +/- (1.202)	0.634 +/- (0.107)
			VGG-16 RAVEN	0.265 +/- (0.007)	0.067 +/- (0.015)	28.445 +/- (1.675)	0.637 +/- (0.113)
			SuperFormer	0.502 +/- (0.019)	0.108 +/- (0.007)	22.883 +/- (1.107)	0.567 +/- (0.085)
			Trilinear	0.502 +/- (0.016)	0.080 +/- (0.009)	26.644 +/- (0.694)	0.587 +/- (0.100)
	Deg. B	2×2×2	ArSSR-RDN	0.236 +/- (0.044)	0.038 +/- (0.013)	33.925 +/- (3.202)	0.820 +/- (0.065)
			ArSSR-ResCNN	0.272 +/- (0.044)	0.044 +/- (0.013)	32.049 +/- (2.786)	0.788 +/- (0.066)
			Lanczos	0.216 +/- (0.038)	0.029 +/- (0.006)	36.305 +/- (1.897)	0.880 +/- (0.019)
			NLMUPSAMPLE	0.248 +/- (0.036)	0.034 +/- (0.007)	34.573 +/- (1.854)	0.856 +/- (0.024)
			RAVEN	0.069 +/- (0.017)	0.024 +/- (0.008)	37.748 +/- (3.891)	0.906 +/- (0.025)
			4× RAVEN	0.092 +/- (0.020)	0.031 +/- (0.008)	35.021 +/- (2.596)	0.883 +/- (0.023)
			Augmented RAVEN	0.082 +/- (0.020)	0.037 +/- (0.009)	32.314 +/- (2.467)	0.887 +/- (0.023)
			In-vivo RAVEN	0.071 +/- (0.019)	0.027 +/- (0.008)	36.839 +/- (3.104)	0.899 +/- (0.027)
			L1 RAVEN	0.110 +/- (0.024)	0.027 +/- (0.009)	36.282 +/- (3.244)	0.907 +/- (0.021)
			VGG-16 RAVEN	0.070 +/- (0.017)	0.028 +/- (0.006)	36.603 +/- (3.037)	0.905 +/- (0.019)
			SuperFormer	0.209 +/- (0.020)	0.057 +/- (0.008)	29.183 +/- (1.879)	0.813 +/- (0.015)
			Trilinear	0.193 +/- (0.025)	0.037 +/- (0.008)	33.830 +/- (1.821)	0.842 +/- (0.026)
		3×3×3	ArSSR-RDN	0.360 +/- (0.049)	0.061 +/- (0.018)	29.380 +/- (2.794)	0.675 +/- (0.090)
			ArSSR-ResCNN	0.407 +/- (0.044)	0.069 +/- (0.018)	27.860 +/- (2.410)	0.638 +/- (0.090)
			Lanczos	0.432 +/- (0.034)	0.054 +/- (0.011)	30.234 +/- (1.842)	0.735 +/- (0.045)
			NLMUPSAMPLE	0.401 +/- (0.033)	0.055 +/- (0.011)	30.047 +/- (1.831)	0.729 +/- (0.044)
			RAVEN	0.254 +/- (0.023)	0.053 +/- (0.017)	30.485 +/- (3.359)	0.772 +/- (0.042)
			4× RAVEN	0.223 +/- (0.014)	0.056 +/- (0.016)	29.573 +/- (2.837)	0.770 +/- (0.045)
			Augmented RAVEN	0.233 +/- (0.024)	0.065 +/- (0.018)	27.554 +/- (2.803)	0.767 +/- (0.045)

			In-vivo RAVEN	0.234 +/- (0.032)	0.052 +/- (0.017)	30.545 +/- (3.037)	0.780 +/- (0.047)
			L1 RAVEN	0.349 +/- (0.024)	0.056 +/- (0.015)	29.498 +/- (2.619)	0.748 +/- (0.044)
			VGG-16 RAVEN	0.221 +/- (0.022)	0.047 +/- (0.010)	31.201 +/- (1.922)	0.801 +/- (0.038)
			SuperFormer	0.442 +/- (0.022)	0.082 +/- (0.003)	25.822 +/- (0.707)	0.649 +/- (0.045)
			Trilinear	0.439 +/- (0.025)	0.065 +/- (0.013)	28.498 +/- (1.799)	0.678 +/- (0.056)
		4×4×4	ArSSR-RDN	0.522 +/- (0.082)	0.100 +/- (0.025)	23.906 +/- (3.602)	0.460 +/- (0.070)
			ArSSR-ResCNN	0.524 +/- (0.068)	0.099 +/- (0.024)	24.018 +/- (3.551)	0.476 +/- (0.066)
			Lanczos	0.569 +/- (0.020)	0.090 +/- (0.022)	25.822 +/- (1.477)	0.542 +/- (0.136)
			NLMUPSAMPLE	0.570 +/- (0.019)	0.092 +/- (0.021)	25.597 +/- (1.408)	0.533 +/- (0.132)
			RAVEN	0.419 +/- (0.013)	0.096 +/- (0.023)	25.186 +/- (2.211)	0.523 +/- (0.136)
			4× RAVEN	0.322 +/- (0.021)	0.091 +/- (0.022)	25.688 +/- (1.947)	0.554 +/- (0.151)
			Augmented RAVEN	0.369 +/- (0.013)	0.104 +/- (0.022)	23.954 +/- (1.912)	0.523 +/- (0.134)
			In-vivo RAVEN	0.368 +/- (0.028)	0.094 +/- (0.023)	25.620 +/- (2.476)	0.531 +/- (0.143)
			L1 RAVEN	0.510 +/- (0.012)	0.095 +/- (0.020)	25.039 +/- (1.450)	0.527 +/- (0.130)
			VGG-16 RAVEN	0.371 +/- (0.013)	0.086 +/- (0.024)	26.381 +/- (1.762)	0.539 +/- (0.138)
			SuperFormer	0.592 +/- (0.020)	0.125 +/- (0.011)	21.660 +/- (0.777)	0.483 +/- (0.107)
			Trilinear	0.596 +/- (0.019)	0.098 +/- (0.021)	24.826 +/- (1.371)	0.503 +/- (0.127)
	Deg. C	2×2×2	ArSSR-RDN	0.194 +/- (0.045)	0.034 +/- (0.012)	35.197 +/- (3.335)	0.830 +/- (0.070)
			ArSSR-ResCNN	0.226 +/- (0.046)	0.040 +/- (0.011)	32.348 +/- (2.259)	0.809 +/- (0.060)
			Lanczos	0.172 +/- (0.038)	0.022 +/- (0.005)	39.203 +/- (2.929)	0.897 +/- (0.020)
			NLMUPSAMPLE	0.192 +/- (0.039)	0.025 +/- (0.005)	37.968 +/- (2.177)	0.890 +/- (0.018)
			RAVEN	0.072 +/- (0.020)	0.023 +/- (0.006)	37.984 +/- (3.198)	0.902 +/- (0.025)
			4× RAVEN	0.090 +/- (0.021)	0.027 +/- (0.007)	36.651 +/- (2.628)	0.888 +/- (0.023)
			Augmented RAVEN	0.088 +/- (0.021)	0.032 +/- (0.007)	33.990 +/- (2.022)	0.882 +/- (0.024)
			In-vivo RAVEN	0.074 +/- (0.019)	0.026 +/- (0.007)	37.278 +/- (3.380)	0.891 +/- (0.026)
			L1 RAVEN	0.100 +/- (0.022)	0.023 +/- (0.006)	37.751 +/- (2.219)	0.908 +/- (0.018)
			VGG-16 RAVEN	0.080 +/- (0.019)	0.028 +/- (0.008)	36.654 +/- (3.512)	0.887 +/- (0.025)
			SuperFormer	0.141 +/- (0.021)	0.050 +/- (0.010)	30.713 +/- (1.832)	0.840 +/- (0.011)
			Trilinear	0.125 +/- (0.023)	0.029 +/- (0.006)	36.387 +/- (2.103)	0.866 +/- (0.021)
		3×3×3	ArSSR-RDN	0.275 +/- (0.052)	0.055 +/- (0.017)	30.120 +/- (2.513)	0.686 +/- (0.088)
			ArSSR-ResCNN	0.298 +/- (0.052)	0.059 +/- (0.016)	28.874 +/- (2.226)	0.677 +/- (0.082)
			Lanczos	0.351 +/- (0.052)	0.036 +/- (0.009)	34.997 +/- (3.078)	0.796 +/- (0.035)
			NLMUPSAMPLE	0.283 +/- (0.049)	0.035 +/- (0.007)	35.113 +/- (2.563)	0.800 +/- (0.028)
			RAVEN	0.208 +/- (0.032)	0.036 +/- (0.010)	34.438 +/- (3.455)	0.805 +/- (0.035)
			4× RAVEN	0.197 +/- (0.024)	0.039 +/- (0.010)	33.105 +/- (2.657)	0.799 +/- (0.037)
			Augmented RAVEN	0.203 +/- (0.034)	0.047 +/- (0.012)	30.764 +/- (2.489)	0.787 +/- (0.040)
			In-vivo RAVEN	0.200 +/- (0.033)	0.038 +/- (0.010)	33.976 +/- (3.052)	0.804 +/- (0.040)
			L1 RAVEN	0.249 +/- (0.035)	0.038 +/- (0.011)	33.552 +/- (2.681)	0.807 +/- (0.034)
			VGG-16 RAVEN	0.208 +/- (0.034)	0.039 +/- (0.009)	33.526 +/- (2.589)	0.800 +/- (0.035)
			SuperFormer	0.317 +/- (0.037)	0.065 +/- (0.005)	28.142 +/- (0.861)	0.727 +/- (0.031)
			Trilinear	0.308 +/- (0.039)	0.046 +/- (0.010)	32.004 +/- (1.873)	0.754 +/- (0.039)
		4×4×4	ArSSR-RDN	0.497 +/- (0.148)	0.108 +/- (0.035)	23.724 +/- (4.115)	0.388 +/- (0.096)
			ArSSR-ResCNN	0.469 +/- (0.104)	0.094 +/- (0.026)	25.028 +/- (3.303)	0.436 +/- (0.073)
			Lanczos	0.462 +/- (0.045)	0.048 +/- (0.013)	32.027 +/- (2.842)	0.709 +/- (0.055)
			NLMUPSAMPLE	0.445 +/- (0.042)	0.049 +/- (0.010)	31.812 +/- (2.038)	0.710 +/- (0.047)
			RAVEN	0.254 +/- (0.019)	0.062 +/- (0.014)	28.966 +/- (3.279)	0.678 +/- (0.057)
			4× RAVEN	0.214 +/- (0.010)	0.056 +/- (0.013)	29.758 +/- (2.795)	0.684 +/- (0.061)
			Augmented RAVEN	0.241 +/- (0.017)	0.068 +/- (0.012)	27.488 +/- (1.236)	0.649 +/- (0.064)
			In-vivo RAVEN	0.242 +/- (0.019)	0.057 +/- (0.012)	30.066 +/- (2.645)	0.673 +/- (0.062)
			L1 RAVEN	0.319 +/- (0.027)	0.056 +/- (0.011)	29.731 +/- (1.752)	0.694 +/- (0.050)
			VGG-16 RAVEN	0.295 +/- (0.015)	0.056 +/- (0.016)	30.326 +/- (2.578)	0.647 +/- (0.064)
			SuperFormer	0.439 +/- (0.027)	0.084 +/- (0.005)	25.161 +/- (0.672)	0.637 +/- (0.048)
			Trilinear	0.432 +/- (0.027)	0.061 +/- (0.013)	29.334 +/- (1.759)	0.658 +/- (0.058)
DBCBB	Deg. A	2×2×2	ArSSR-RDN	0.169 +/- (0.043)	0.065 +/- (0.024)	28.188 +/- (3.886)	0.807 +/- (0.087)
			ArSSR-ResCNN	0.210 +/- (0.042)	0.078 +/- (0.026)	26.130 +/- (3.462)	0.761 +/- (0.092)

			Lanczos	0.148 +/- (0.035)	0.043 +/- (0.012)	33.082 +/- (3.017)	0.886 +/- (0.035)
			NLMUPSAMPLE	0.155 +/- (0.028)	0.047 +/- (0.011)	31.570 +/- (2.674)	0.891 +/- (0.026)
			RAVEN	0.038 +/- (0.007)	0.044 +/- (0.018)	31.459 +/- (3.900)	0.926 +/- (0.020)
			4× RAVEN	0.061 +/- (0.009)	0.050 +/- (0.013)	30.874 +/- (2.465)	0.887 +/- (0.023)
			Augmented RAVEN	0.047 +/- (0.010)	0.048 +/- (0.014)	31.283 +/- (2.474)	0.898 +/- (0.022)
			In-vivo RAVEN	0.052 +/- (0.010)	0.054 +/- (0.015)	29.581 +/- (2.941)	0.893 +/- (0.020)
			L1 RAVEN	0.051 +/- (0.013)	0.047 +/- (0.020)	31.125 +/- (4.172)	0.916 +/- (0.018)
			VGG-16 RAVEN	0.045 +/- (0.010)	0.047 +/- (0.010)	31.461 +/- (2.045)	0.904 +/- (0.021)
			SuperFormer	0.155 +/- (0.014)	0.085 +/- (0.028)	26.510 +/- (3.931)	0.807 +/- (0.036)
			Trilinear	0.132 +/- (0.022)	0.052 +/- (0.011)	31.129 +/- (2.436)	0.856 +/- (0.030)
			ArSSR-RDN	0.281 +/- (0.046)	0.102 +/- (0.029)	24.397 +/- (2.970)	0.613 +/- (0.093)
			ArSSR-ResCNN	0.322 +/- (0.037)	0.112 +/- (0.029)	23.424 +/- (2.878)	0.572 +/- (0.093)
			Lanczos	0.336 +/- (0.049)	0.074 +/- (0.016)	27.836 +/- (2.183)	0.733 +/- (0.058)
			NLMUPSAMPLE	0.272 +/- (0.039)	0.071 +/- (0.015)	28.160 +/- (2.261)	0.756 +/- (0.050)
			RAVEN	0.140 +/- (0.021)	0.062 +/- (0.021)	28.704 +/- (3.330)	0.848 +/- (0.035)
		3×3×3	4× RAVEN	0.120 +/- (0.018)	0.060 +/- (0.016)	29.010 +/- (2.502)	0.837 +/- (0.033)
			Augmented RAVEN	0.136 +/- (0.027)	0.065 +/- (0.018)	28.169 +/- (2.596)	0.835 +/- (0.036)
			In-vivo RAVEN	0.153 +/- (0.029)	0.072 +/- (0.023)	27.346 +/- (3.374)	0.815 +/- (0.046)
			L1 RAVEN	0.188 +/- (0.023)	0.068 +/- (0.021)	28.038 +/- (3.175)	0.812 +/- (0.041)
			VGG-16 RAVEN	0.124 +/- (0.022)	0.059 +/- (0.015)	28.859 +/- (2.723)	0.861 +/- (0.029)
			SuperFormer	0.365 +/- (0.022)	0.113 +/- (0.025)	23.553 +/- (2.662)	0.623 +/- (0.062)
			Trilinear	0.342 +/- (0.041)	0.089 +/- (0.017)	26.133 +/- (1.979)	0.662 +/- (0.061)
			ArSSR-RDN	0.381 +/- (0.036)	0.121 +/- (0.032)	22.933 +/- (3.083)	0.525 +/- (0.092)
			ArSSR-ResCNN	0.382 +/- (0.036)	0.123 +/- (0.034)	22.494 +/- (3.202)	0.514 +/- (0.099)
			Lanczos	0.491 +/- (0.036)	0.143 +/- (0.028)	21.992 +/- (1.889)	0.408 +/- (0.072)
			NLMUPSAMPLE	0.479 +/- (0.032)	0.143 +/- (0.028)	22.146 +/- (1.909)	0.408 +/- (0.072)
			RAVEN	0.288 +/- (0.036)	0.149 +/- (0.031)	21.832 +/- (2.247)	0.399 +/- (0.070)
		4×4×4	4× RAVEN	0.195 +/- (0.017)	0.147 +/- (0.030)	22.081 +/- (2.307)	0.391 +/- (0.065)
			Augmented RAVEN	0.277 +/- (0.031)	0.150 +/- (0.030)	21.509 +/- (2.098)	0.398 +/- (0.066)
			In-vivo RAVEN	0.279 +/- (0.034)	0.152 +/- (0.031)	21.701 +/- (2.401)	0.393 +/- (0.065)
			L1 RAVEN	0.350 +/- (0.024)	0.147 +/- (0.031)	21.963 +/- (2.273)	0.406 +/- (0.070)
			VGG-16 RAVEN	0.273 +/- (0.028)	0.143 +/- (0.028)	22.323 +/- (2.033)	0.407 +/- (0.067)
			SuperFormer	0.514 +/- (0.026)	0.155 +/- (0.027)	20.843 +/- (1.769)	0.378 +/- (0.067)
			Trilinear	0.514 +/- (0.032)	0.149 +/- (0.028)	21.536 +/- (1.839)	0.381 +/- (0.069)
			ArSSR-RDN	0.208 +/- (0.040)	0.072 +/- (0.026)	27.190 +/- (3.814)	0.783 +/- (0.084)
			ArSSR-ResCNN	0.243 +/- (0.037)	0.082 +/- (0.027)	25.871 +/- (3.521)	0.739 +/- (0.088)
			Lanczos	0.191 +/- (0.035)	0.050 +/- (0.011)	31.396 +/- (2.426)	0.873 +/- (0.029)
			NLMUPSAMPLE	0.215 +/- (0.032)	0.057 +/- (0.012)	29.803 +/- (2.576)	0.846 +/- (0.032)
			RAVEN	0.043 +/- (0.008)	0.045 +/- (0.020)	31.298 +/- (4.262)	0.932 +/- (0.018)
		2×2×2	4× RAVEN	0.063 +/- (0.010)	0.050 +/- (0.014)	30.772 +/- (2.651)	0.891 +/- (0.022)
			Augmented RAVEN	0.050 +/- (0.013)	0.047 +/- (0.014)	31.157 +/- (2.626)	0.907 +/- (0.021)
			In-vivo RAVEN	0.057 +/- (0.013)	0.054 +/- (0.017)	29.363 +/- (3.263)	0.902 +/- (0.021)
			L1 RAVEN	0.063 +/- (0.018)	0.048 +/- (0.021)	30.938 +/- (4.395)	0.921 +/- (0.019)
			VGG-16 RAVEN	0.047 +/- (0.012)	0.045 +/- (0.010)	31.477 +/- (2.275)	0.917 +/- (0.019)
	Deg. B		SuperFormer	0.213 +/- (0.016)	0.090 +/- (0.026)	25.698 +/- (3.460)	0.772 +/- (0.041)
			Trilinear	0.187 +/- (0.026)	0.062 +/- (0.013)	29.238 +/- (2.419)	0.817 +/- (0.036)
			ArSSR-RDN	0.344 +/- (0.040)	0.113 +/- (0.031)	23.210 +/- (3.012)	0.563 +/- (0.091)
			ArSSR-ResCNN	0.387 +/- (0.032)	0.122 +/- (0.031)	22.536 +/- (2.988)	0.522 +/- (0.091)
			Lanczos	0.395 +/- (0.045)	0.088 +/- (0.017)	26.156 +/- (1.999)	0.663 +/- (0.064)
			NLMUPSAMPLE	0.369 +/- (0.041)	0.089 +/- (0.017)	26.055 +/- (2.020)	0.659 +/- (0.064)
		3×3×3	RAVEN	0.216 +/- (0.028)	0.077 +/- (0.021)	26.815 +/- (2.804)	0.768 +/- (0.052)
			4× RAVEN	0.146 +/- (0.020)	0.070 +/- (0.018)	27.619 +/- (2.371)	0.797 +/- (0.039)
			Augmented RAVEN	0.200 +/- (0.029)	0.079 +/- (0.019)	26.440 +/- (2.410)	0.767 +/- (0.047)
			In-vivo RAVEN	0.206 +/- (0.033)	0.082 +/- (0.023)	26.341 +/- (2.987)	0.756 +/- (0.057)
			L1 RAVEN	0.281 +/- (0.024)	0.084 +/- (0.020)	26.210 +/- (2.477)	0.718 +/- (0.057)

			VGG-16 RAVEN	0.180 +/- (0.027)	0.070 +/- (0.015)	27.505 +/- (2.477)	0.802 +/- (0.038)
			SuperFormer	0.444 +/- (0.023)	0.127 +/- (0.026)	22.411 +/- (2.314)	0.547 +/- (0.073)
			Trilinear	0.422 +/- (0.042)	0.103 +/- (0.019)	24.629 +/- (1.796)	0.584 +/- (0.071)
			ArSSR-RDN	0.481 +/- (0.030)	0.133 +/- (0.025)	22.340 +/- (1.868)	0.448 +/- (0.081)
			ArSSR-ResCNN	0.483 +/- (0.030)	0.142 +/- (0.036)	21.174 +/- (2.973)	0.429 +/- (0.091)
			Lanczos	0.549 +/- (0.029)	0.149 +/- (0.028)	21.484 +/- (1.820)	0.381 +/- (0.070)
			NLMUPSAMPLE	0.547 +/- (0.028)	0.150 +/- (0.028)	21.388 +/- (1.801)	0.375 +/- (0.070)
			RAVEN	0.407 +/- (0.041)	0.154 +/- (0.031)	21.145 +/- (2.061)	0.374 +/- (0.068)
		4×4×4	4× RAVEN	0.268 +/- (0.028)	0.149 +/- (0.030)	21.655 +/- (2.321)	0.392 +/- (0.064)
			Augmented RAVEN	0.390 +/- (0.037)	0.155 +/- (0.030)	20.993 +/- (1.992)	0.376 +/- (0.064)
			In-vivo RAVEN	0.377 +/- (0.040)	0.156 +/- (0.031)	21.190 +/- (2.190)	0.370 +/- (0.065)
			L1 RAVEN	0.489 +/- (0.025)	0.153 +/- (0.030)	21.191 +/- (1.992)	0.376 +/- (0.069)
			VGG-16 RAVEN	0.377 +/- (0.028)	0.147 +/- (0.028)	21.666 +/- (1.872)	0.390 +/- (0.065)
			SuperFormer	0.583 +/- (0.026)	0.163 +/- (0.028)	20.204 +/- (1.688)	0.346 +/- (0.066)
			Trilinear	0.581 +/- (0.029)	0.157 +/- (0.029)	20.810 +/- (1.729)	0.347 +/- (0.068)
			ArSSR-RDN	0.167 +/- (0.045)	0.063 +/- (0.022)	28.303 +/- (3.609)	0.810 +/- (0.082)
			ArSSR-ResCNN	0.202 +/- (0.039)	0.075 +/- (0.023)	26.487 +/- (3.274)	0.774 +/- (0.085)
			Lanczos	0.137 +/- (0.034)	0.042 +/- (0.012)	32.147 +/- (2.960)	0.893 +/- (0.037)
			NLMUPSAMPLE	0.156 +/- (0.032)	0.043 +/- (0.010)	32.715 +/- (2.358)	0.897 +/- (0.026)
			RAVEN	0.044 +/- (0.009)	0.042 +/- (0.016)	31.824 +/- (3.463)	0.925 +/- (0.020)
		2×2×2	4× RAVEN	0.063 +/- (0.010)	0.047 +/- (0.011)	31.163 +/- (2.231)	0.894 +/- (0.020)
			Augmented RAVEN	0.053 +/- (0.013)	0.046 +/- (0.013)	31.415 +/- (2.214)	0.901 +/- (0.020)
			In-vivo RAVEN	0.057 +/- (0.014)	0.051 +/- (0.014)	30.012 +/- (2.530)	0.893 +/- (0.017)
			L1 RAVEN	0.054 +/- (0.014)	0.044 +/- (0.016)	31.434 +/- (3.435)	0.919 +/- (0.015)
			VGG-16 RAVEN	0.053 +/- (0.013)	0.045 +/- (0.009)	31.362 +/- (1.721)	0.901 +/- (0.019)
			SuperFormer	0.141 +/- (0.013)	0.086 +/- (0.029)	26.357 +/- (3.689)	0.811 +/- (0.036)
			Trilinear	0.118 +/- (0.024)	0.052 +/- (0.012)	31.254 +/- (2.171)	0.852 +/- (0.035)
			ArSSR-RDN	0.275 +/- (0.051)	0.100 +/- (0.024)	24.362 +/- (2.493)	0.615 +/- (0.087)
			ArSSR-ResCNN	0.300 +/- (0.041)	0.107 +/- (0.024)	23.625 +/- (2.442)	0.590 +/- (0.087)
			Lanczos	0.326 +/- (0.055)	0.071 +/- (0.019)	27.593 +/- (2.926)	0.753 +/- (0.065)
			NLMUPSAMPLE	0.262 +/- (0.048)	0.065 +/- (0.016)	28.747 +/- (2.444)	0.771 +/- (0.055)
			RAVEN	0.180 +/- (0.031)	0.062 +/- (0.017)	28.775 +/- (2.805)	0.811 +/- (0.046)
	Deg. C	3×3×3	4× RAVEN	0.151 +/- (0.026)	0.059 +/- (0.015)	29.302 +/- (2.271)	0.819 +/- (0.039)
			Augmented RAVEN	0.172 +/- (0.043)	0.064 +/- (0.016)	28.456 +/- (2.289)	0.802 +/- (0.047)
			In-vivo RAVEN	0.178 +/- (0.040)	0.070 +/- (0.019)	27.625 +/- (2.736)	0.793 +/- (0.047)
			L1 RAVEN	0.193 +/- (0.034)	0.066 +/- (0.020)	28.381 +/- (3.135)	0.807 +/- (0.045)
			VGG-16 RAVEN	0.176 +/- (0.041)	0.061 +/- (0.012)	28.809 +/- (1.882)	0.808 +/- (0.043)
			SuperFormer	0.341 +/- (0.032)	0.110 +/- (0.027)	23.818 +/- (2.683)	0.651 +/- (0.062)
			Trilinear	0.321 +/- (0.051)	0.081 +/- (0.017)	26.816 +/- (2.010)	0.690 +/- (0.063)
			ArSSR-RDN	0.372 +/- (0.045)	0.141 +/- (0.025)	22.761 +/- (1.537)	0.393 +/- (0.067)
			ArSSR-ResCNN	0.374 +/- (0.041)	0.146 +/- (0.033)	22.193 +/- (2.575)	0.381 +/- (0.071)
			Lanczos	0.430 +/- (0.055)	0.094 +/- (0.025)	24.795 +/- (2.956)	0.623 +/- (0.089)
			NLMUPSAMPLE	0.415 +/- (0.052)	0.093 +/- (0.024)	25.109 +/- (2.834)	0.620 +/- (0.088)
			RAVEN	0.255 +/- (0.042)	0.101 +/- (0.025)	24.225 +/- (2.680)	0.626 +/- (0.079)
		4×4×4	4× RAVEN	0.171 +/- (0.026)	0.083 +/- (0.019)	26.208 +/- (2.307)	0.692 +/- (0.067)
			Augmented RAVEN	0.235 +/- (0.046)	0.101 +/- (0.024)	24.094 +/- (2.384)	0.612 +/- (0.077)
			In-vivo RAVEN	0.243 +/- (0.046)	0.104 +/- (0.024)	23.979 +/- (2.449)	0.612 +/- (0.072)
			L1 RAVEN	0.291 +/- (0.049)	0.106 +/- (0.028)	23.420 +/- (2.931)	0.620 +/- (0.079)
			VGG-16 RAVEN	0.289 +/- (0.048)	0.099 +/- (0.019)	24.706 +/- (1.812)	0.578 +/- (0.072)
			SuperFormer	0.444 +/- (0.054)	0.118 +/- (0.022)	22.701 +/- (1.831)	0.542 +/- (0.082)
			Trilinear	0.435 +/- (0.053)	0.106 +/- (0.022)	24.060 +/- (2.178)	0.553 +/- (0.084)
			ArSSR-RDN	0.182 +/- (0.020)	0.036 +/- (0.019)	34.700 +/- (6.384)	0.877 +/- (0.027)
			ArSSR-ResCNN	0.175 +/- (0.013)	0.048 +/- (0.009)	29.131 +/- (3.493)	0.876 +/- (0.015)
HBA	Deg. A	2×2×2	Lanczos	0.018 +/- (0.009)	0.011 +/- (0.011)	46.514 +/- (12.601)	0.989 +/- (0.006)
			NLMUPSAMPLE	0.039 +/- (0.012)	0.015 +/- (0.013)	43.722 +/- (10.850)	0.981 +/- (0.007)

			RAVEN	0.097 +/- (0.011)	0.050 +/- (0.039)	29.900 +/- (10.087)	0.939 +/- (0.005)
			4× RAVEN	0.148 +/- (0.010)	0.055 +/- (0.037)	29.013 +/- (8.775)	0.908 +/- (0.000)
			Augmented RAVEN	0.127 +/- (0.024)	0.043 +/- (0.027)	30.685 +/- (7.962)	0.927 +/- (0.004)
			In-vivo RAVEN	0.127 +/- (0.020)	0.055 +/- (0.031)	28.106 +/- (7.141)	0.920 +/- (0.013)
			L1 RAVEN	0.077 +/- (0.002)	0.038 +/- (0.025)	32.082 +/- (8.904)	0.957 +/- (0.001)
			VGG-16 RAVEN	0.130 +/- (0.005)	0.050 +/- (0.019)	29.373 +/- (3.827)	0.925 +/- (0.014)
			SuperFormer	0.156 +/- (0.058)	0.117 +/- (0.031)	19.741 +/- (2.170)	0.881 +/- (0.047)
			Trilinear	0.065 +/- (0.003)	0.019 +/- (0.016)	41.792 +/- (10.981)	0.971 +/- (0.010)
			ArSSR-RDN	0.123 +/- (0.003)	0.033 +/- (0.008)	35.780 +/- (4.062)	0.849 +/- (0.012)
			ArSSR-ResCNN	0.140 +/- (0.007)	0.035 +/- (0.010)	35.471 +/- (4.389)	0.843 +/- (0.016)
			Lanczos	0.111 +/- (0.015)	0.026 +/- (0.020)	38.950 +/- (10.030)	0.943 +/- (0.016)
			NLMUPSAMPLE	0.099 +/- (0.007)	0.026 +/- (0.019)	38.612 +/- (9.247)	0.940 +/- (0.014)
			RAVEN	0.073 +/- (0.004)	0.052 +/- (0.040)	29.573 +/- (10.231)	0.944 +/- (0.003)
		3×3×3	4× RAVEN	0.141 +/- (0.003)	0.057 +/- (0.041)	28.921 +/- (9.198)	0.910 +/- (0.004)
			Augmented RAVEN	0.101 +/- (0.010)	0.046 +/- (0.030)	30.331 +/- (8.469)	0.934 +/- (0.004)
			In-vivo RAVEN	0.103 +/- (0.011)	0.056 +/- (0.041)	28.836 +/- (9.522)	0.928 +/- (0.011)
			L1 RAVEN	0.091 +/- (0.010)	0.035 +/- (0.024)	32.944 +/- (9.309)	0.948 +/- (0.004)
			VGG-16 RAVEN	0.096 +/- (0.007)	0.041 +/- (0.007)	31.321 +/- (1.449)	0.936 +/- (0.015)
			SuperFormer	0.269 +/- (0.066)	0.121 +/- (0.028)	19.557 +/- (1.972)	0.817 +/- (0.041)
			Trilinear	0.184 +/- (0.009)	0.031 +/- (0.017)	36.153 +/- (6.670)	0.903 +/- (0.013)
			ArSSR-RDN	0.224 +/- (0.018)	0.048 +/- (0.010)	29.694 +/- (2.201)	0.808 +/- (0.015)
			ArSSR-ResCNN	0.238 +/- (0.010)	0.042 +/- (0.013)	31.704 +/- (4.256)	0.822 +/- (0.011)
			Lanczos	0.283 +/- (0.004)	0.059 +/- (0.007)	28.979 +/- (1.333)	0.724 +/- (0.020)
			NLMUPSAMPLE	0.289 +/- (0.001)	0.059 +/- (0.007)	28.978 +/- (1.299)	0.725 +/- (0.020)
			RAVEN	0.210 +/- (0.019)	0.092 +/- (0.033)	23.863 +/- (5.348)	0.709 +/- (0.014)
		4×4×4	4× RAVEN	0.235 +/- (0.004)	0.105 +/- (0.042)	22.730 +/- (5.645)	0.689 +/- (0.017)
			Augmented RAVEN	0.222 +/- (0.024)	0.095 +/- (0.031)	23.470 +/- (4.803)	0.701 +/- (0.018)
			In-vivo RAVEN	0.220 +/- (0.017)	0.098 +/- (0.038)	23.252 +/- (5.542)	0.700 +/- (0.018)
			L1 RAVEN	0.236 +/- (0.009)	0.080 +/- (0.017)	25.129 +/- (3.405)	0.716 +/- (0.014)
			VGG-16 RAVEN	0.226 +/- (0.033)	0.066 +/- (0.005)	28.491 +/- (0.883)	0.709 +/- (0.026)
			SuperFormer	0.340 +/- (0.002)	0.058 +/- (0.009)	31.078 +/- (2.639)	0.710 +/- (0.023)
			Trilinear	0.352 +/- (0.004)	0.060 +/- (0.007)	28.772 +/- (1.137)	0.714 +/- (0.021)
			ArSSR-RDN	0.188 +/- (0.010)	0.036 +/- (0.018)	33.990 +/- (5.302)	0.876 +/- (0.024)
			ArSSR-ResCNN	0.189 +/- (0.015)	0.053 +/- (0.015)	28.163 +/- (4.422)	0.868 +/- (0.011)
			Lanczos	0.043 +/- (0.013)	0.018 +/- (0.017)	42.386 +/- (11.769)	0.979 +/- (0.010)
			NLMUPSAMPLE	0.069 +/- (0.015)	0.021 +/- (0.018)	40.791 +/- (10.549)	0.967 +/- (0.011)
			RAVEN	0.085 +/- (0.011)	0.050 +/- (0.039)	29.941 +/- (10.144)	0.947 +/- (0.006)
		2×2×2	4× RAVEN	0.143 +/- (0.008)	0.055 +/- (0.039)	29.106 +/- (9.089)	0.914 +/- (0.000)
			Augmented RAVEN	0.115 +/- (0.024)	0.043 +/- (0.028)	30.916 +/- (8.247)	0.935 +/- (0.004)
			In-vivo RAVEN	0.117 +/- (0.019)	0.054 +/- (0.033)	28.439 +/- (7.859)	0.926 +/- (0.012)
			L1 RAVEN	0.071 +/- (0.011)	0.036 +/- (0.026)	32.701 +/- (9.691)	0.963 +/- (0.003)
			VGG-16 RAVEN	0.111 +/- (0.003)	0.049 +/- (0.017)	29.565 +/- (3.479)	0.936 +/- (0.013)
			SuperFormer	0.183 +/- (0.059)	0.119 +/- (0.027)	19.556 +/- (1.812)	0.869 +/- (0.043)
	Deg. B		Trilinear	0.094 +/- (0.009)	0.024 +/- (0.019)	39.761 +/- (10.111)	0.957 +/- (0.013)
			ArSSR-RDN	0.192 +/- (0.004)	0.041 +/- (0.014)	33.341 +/- (4.181)	0.832 +/- (0.018)
			ArSSR-ResCNN	0.215 +/- (0.002)	0.044 +/- (0.019)	33.062 +/- (5.299)	0.822 +/- (0.023)
			Lanczos	0.190 +/- (0.004)	0.031 +/- (0.018)	36.184 +/- (6.759)	0.908 +/- (0.013)
			NLMUPSAMPLE	0.181 +/- (0.005)	0.032 +/- (0.017)	35.842 +/- (6.292)	0.903 +/- (0.012)
			RAVEN	0.102 +/- (0.004)	0.057 +/- (0.041)	28.775 +/- (9.810)	0.930 +/- (0.003)
		3×3×3	4× RAVEN	0.154 +/- (0.006)	0.063 +/- (0.041)	27.531 +/- (8.018)	0.906 +/- (0.003)
			Augmented RAVEN	0.112 +/- (0.001)	0.053 +/- (0.033)	28.886 +/- (7.939)	0.927 +/- (0.004)
			In-vivo RAVEN	0.114 +/- (0.001)	0.058 +/- (0.041)	28.314 +/- (8.995)	0.925 +/- (0.004)
			L1 RAVEN	0.142 +/- (0.007)	0.040 +/- (0.021)	31.319 +/- (7.165)	0.923 +/- (0.001)
			VGG-16 RAVEN	0.105 +/- (0.005)	0.039 +/- (0.003)	31.897 +/- (0.285)	0.932 +/- (0.014)
			SuperFormer	0.335 +/- (0.074)	0.119 +/- (0.026)	19.823 +/- (1.966)	0.787 +/- (0.037)

			Trilinear	0.261 +/- (0.000)	0.036 +/- (0.015)	34.092 +/- (4.657)	0.868 +/- (0.011)
		4×4×4	ArSSR-RDN	0.322 +/- (0.038)	0.049 +/- (0.003)	30.048 +/- (0.413)	0.775 +/- (0.010)
			ArSSR-ResCNN	0.337 +/- (0.030)	0.043 +/- (0.007)	32.071 +/- (2.153)	0.780 +/- (0.011)
			Lanczos	0.373 +/- (0.015)	0.061 +/- (0.006)	28.500 +/- (0.949)	0.714 +/- (0.020)
			NLMUPSAMPLE	0.379 +/- (0.020)	0.062 +/- (0.006)	28.349 +/- (0.845)	0.712 +/- (0.020)
			RAVEN	0.300 +/- (0.023)	0.099 +/- (0.038)	23.049 +/- (5.400)	0.702 +/- (0.011)
			4× RAVEN	0.268 +/- (0.012)	0.115 +/- (0.048)	21.551 +/- (5.405)	0.698 +/- (0.012)
			Augmented RAVEN	0.291 +/- (0.030)	0.105 +/- (0.038)	22.417 +/- (5.052)	0.697 +/- (0.015)
			In-vivo RAVEN	0.287 +/- (0.035)	0.106 +/- (0.041)	22.229 +/- (5.085)	0.699 +/- (0.012)
			L1 RAVEN	0.344 +/- (0.027)	0.093 +/- (0.031)	23.985 +/- (4.025)	0.693 +/- (0.001)
			VGG-16 RAVEN	0.288 +/- (0.030)	0.069 +/- (0.002)	27.846 +/- (0.347)	0.706 +/- (0.024)
			SuperFormer	0.420 +/- (0.019)	0.060 +/- (0.010)	30.773 +/- (2.983)	0.694 +/- (0.023)
			Trilinear	0.432 +/- (0.014)	0.064 +/- (0.005)	27.895 +/- (0.708)	0.698 +/- (0.022)
	Deg. C	2×2×2	ArSSR-RDN	0.185 +/- (0.021)	0.037 +/- (0.019)	33.898 +/- (5.686)	0.872 +/- (0.030)
			ArSSR-ResCNN	0.174 +/- (0.016)	0.049 +/- (0.008)	28.804 +/- (3.379)	0.873 +/- (0.017)
			Lanczos	0.013 +/- (0.009)	0.011 +/- (0.012)	46.516 +/- (14.562)	0.989 +/- (0.007)
			NLMUPSAMPLE	0.028 +/- (0.013)	0.014 +/- (0.014)	44.494 +/- (13.007)	0.984 +/- (0.008)
			RAVEN	0.097 +/- (0.010)	0.049 +/- (0.038)	30.118 +/- (10.029)	0.936 +/- (0.006)
			4× RAVEN	0.150 +/- (0.009)	0.054 +/- (0.035)	28.784 +/- (8.150)	0.904 +/- (0.000)
			Augmented RAVEN	0.128 +/- (0.021)	0.043 +/- (0.027)	30.972 +/- (8.080)	0.922 +/- (0.003)
			In-vivo RAVEN	0.132 +/- (0.021)	0.056 +/- (0.025)	27.464 +/- (5.632)	0.914 +/- (0.014)
			L1 RAVEN	0.083 +/- (0.002)	0.040 +/- (0.022)	30.739 +/- (6.960)	0.950 +/- (0.002)
			VGG-16 RAVEN	0.138 +/- (0.010)	0.054 +/- (0.024)	28.774 +/- (4.602)	0.915 +/- (0.016)
			SuperFormer	0.149 +/- (0.052)	0.120 +/- (0.029)	19.585 +/- (2.079)	0.882 +/- (0.045)
			Trilinear	0.054 +/- (0.002)	0.020 +/- (0.019)	41.817 +/- (12.516)	0.972 +/- (0.012)
		3×3×3	ArSSR-RDN	0.100 +/- (0.003)	0.034 +/- (0.009)	35.825 +/- (4.139)	0.838 +/- (0.011)
			ArSSR-ResCNN	0.105 +/- (0.009)	0.033 +/- (0.007)	36.286 +/- (3.373)	0.839 +/- (0.014)
			Lanczos	0.075 +/- (0.018)	0.024 +/- (0.021)	39.279 +/- (11.819)	0.950 +/- (0.017)
			NLMUPSAMPLE	0.058 +/- (0.009)	0.023 +/- (0.019)	39.197 +/- (10.836)	0.949 +/- (0.015)
			RAVEN	0.073 +/- (0.003)	0.050 +/- (0.034)	29.416 +/- (8.926)	0.925 +/- (0.005)
			4× RAVEN	0.129 +/- (0.001)	0.057 +/- (0.037)	28.449 +/- (8.225)	0.890 +/- (0.001)
			Augmented RAVEN	0.104 +/- (0.004)	0.044 +/- (0.028)	30.643 +/- (8.083)	0.909 +/- (0.004)
			In-vivo RAVEN	0.108 +/- (0.019)	0.057 +/- (0.035)	28.116 +/- (7.955)	0.903 +/- (0.017)
			L1 RAVEN	0.080 +/- (0.013)	0.039 +/- (0.024)	31.549 +/- (8.347)	0.936 +/- (0.006)
			VGG-16 RAVEN	0.111 +/- (0.009)	0.046 +/- (0.017)	30.252 +/- (3.686)	0.903 +/- (0.019)
			SuperFormer	0.224 +/- (0.055)	0.126 +/- (0.029)	19.209 +/- (1.977)	0.831 +/- (0.042)
			Trilinear	0.131 +/- (0.013)	0.029 +/- (0.020)	37.654 +/- (8.770)	0.920 +/- (0.017)
		4×4×4	ArSSR-RDN	0.183 +/- (0.000)	0.046 +/- (0.011)	33.143 +/- (2.934)	0.750 +/- (0.017)
			ArSSR-ResCNN	0.189 +/- (0.002)	0.048 +/- (0.015)	32.992 +/- (4.424)	0.754 +/- (0.021)
			Lanczos	0.176 +/- (0.001)	0.029 +/- (0.012)	36.643 +/- (5.325)	0.872 +/- (0.007)
			NLMUPSAMPLE	0.172 +/- (0.001)	0.030 +/- (0.013)	36.482 +/- (5.461)	0.874 +/- (0.008)
			RAVEN	0.135 +/- (0.033)	0.063 +/- (0.026)	26.568 +/- (5.291)	0.837 +/- (0.003)
			4× RAVEN	0.188 +/- (0.003)	0.080 +/- (0.018)	24.031 +/- (2.971)	0.789 +/- (0.003)
			Augmented RAVEN	0.171 +/- (0.025)	0.057 +/- (0.018)	27.737 +/- (4.367)	0.814 +/- (0.001)
			In-vivo RAVEN	0.168 +/- (0.014)	0.069 +/- (0.025)	25.549 +/- (4.666)	0.816 +/- (0.006)
			L1 RAVEN	0.140 +/- (0.006)	0.049 +/- (0.011)	28.980 +/- (3.677)	0.850 +/- (0.006)
			VGG-16 RAVEN	0.201 +/- (0.027)	0.046 +/- (0.007)	30.846 +/- (1.478)	0.809 +/- (0.006)
			SuperFormer	0.259 +/- (0.006)	0.138 +/- (0.026)	19.005 +/- (1.626)	0.786 +/- (0.015)
			Trilinear	0.240 +/- (0.005)	0.036 +/- (0.013)	34.492 +/- (4.464)	0.841 +/- (0.010)
HCP	Deg. A	2×2×2	ArSSR-RDN	0.178 +/- (0.032)	0.043 +/- (0.011)	31.334 +/- (2.923)	0.838 +/- (0.028)
			ArSSR-ResCNN	0.231 +/- (0.030)	0.050 +/- (0.013)	30.016 +/- (3.121)	0.797 +/- (0.025)
			Lanczos	0.152 +/- (0.026)	0.027 +/- (0.004)	37.395 +/- (1.398)	0.891 +/- (0.026)
			NLMUPSAMPLE	0.165 +/- (0.025)	0.030 +/- (0.004)	36.019 +/- (1.435)	0.884 +/- (0.020)
			RAVEN	0.048 +/- (0.010)	0.025 +/- (0.003)	37.361 +/- (1.149)	0.911 +/- (0.017)
			4× RAVEN	0.067 +/- (0.012)	0.031 +/- (0.003)	35.161 +/- (1.186)	0.891 +/- (0.018)

			Augmented RAVEN	0.059 +/- (0.011)	0.030 +/- (0.005)	35.416 +/- (1.738)	0.894 +/- (0.018)
			In-vivo RAVEN	0.052 +/- (0.008)	0.031 +/- (0.003)	35.459 +/- (1.429)	0.897 +/- (0.016)
			L1 RAVEN	0.064 +/- (0.012)	0.025 +/- (0.003)	37.429 +/- (1.201)	0.911 +/- (0.015)
			VGG-16 RAVEN	0.052 +/- (0.009)	0.035 +/- (0.007)	34.003 +/- (2.727)	0.895 +/- (0.016)
			SuperFormer	0.141 +/- (0.010)	0.052 +/- (0.014)	30.227 +/- (2.958)	0.841 +/- (0.016)
			Trilinear	0.125 +/- (0.011)	0.033 +/- (0.004)	35.458 +/- (1.565)	0.862 +/- (0.020)
		3×3×3	ArSSR-RDN	0.281 +/- (0.042)	0.066 +/- (0.012)	27.728 +/- (2.333)	0.682 +/- (0.032)
			ArSSR-ResCNN	0.342 +/- (0.028)	0.074 +/- (0.017)	26.740 +/- (2.825)	0.642 +/- (0.028)
			Lanczos	0.354 +/- (0.031)	0.048 +/- (0.006)	31.667 +/- (1.560)	0.750 +/- (0.031)
			NLMUPSAMPLE	0.295 +/- (0.027)	0.047 +/- (0.006)	31.910 +/- (1.587)	0.762 +/- (0.027)
			RAVEN	0.132 +/- (0.022)	0.037 +/- (0.007)	33.546 +/- (1.971)	0.847 +/- (0.026)
			4× RAVEN	0.132 +/- (0.019)	0.042 +/- (0.006)	32.172 +/- (1.580)	0.829 +/- (0.022)
			Augmented RAVEN	0.131 +/- (0.023)	0.039 +/- (0.008)	32.792 +/- (2.055)	0.841 +/- (0.023)
			In-vivo RAVEN	0.127 +/- (0.023)	0.039 +/- (0.007)	32.901 +/- (1.867)	0.848 +/- (0.023)
			L1 RAVEN	0.193 +/- (0.030)	0.039 +/- (0.007)	33.274 +/- (1.814)	0.826 +/- (0.023)
			VGG-16 RAVEN	0.122 +/- (0.019)	0.042 +/- (0.008)	32.447 +/- (2.438)	0.852 +/- (0.021)
			SuperFormer	0.340 +/- (0.016)	0.074 +/- (0.015)	27.117 +/- (2.473)	0.679 +/- (0.022)
			Trilinear	0.334 +/- (0.015)	0.059 +/- (0.008)	29.503 +/- (1.764)	0.697 +/- (0.026)
		4×4×4	ArSSR-RDN	0.348 +/- (0.055)	0.077 +/- (0.010)	27.332 +/- (1.542)	0.588 +/- (0.039)
			ArSSR-ResCNN	0.371 +/- (0.051)	0.075 +/- (0.011)	27.509 +/- (1.540)	0.598 +/- (0.034)
			Lanczos	0.510 +/- (0.020)	0.093 +/- (0.009)	25.918 +/- (1.316)	0.493 +/- (0.028)
			NLMUPSAMPLE	0.500 +/- (0.020)	0.094 +/- (0.009)	25.799 +/- (1.354)	0.491 +/- (0.026)
			RAVEN	0.257 +/- (0.018)	0.094 +/- (0.009)	25.753 +/- (1.500)	0.486 +/- (0.028)
			4× RAVEN	0.203 +/- (0.017)	0.093 +/- (0.008)	26.076 +/- (1.322)	0.496 +/- (0.025)
			Augmented RAVEN	0.235 +/- (0.012)	0.093 +/- (0.009)	25.814 +/- (1.348)	0.484 +/- (0.025)
			In-vivo RAVEN	0.227 +/- (0.015)	0.094 +/- (0.010)	25.888 +/- (1.484)	0.486 +/- (0.023)
			L1 RAVEN	0.324 +/- (0.022)	0.092 +/- (0.009)	26.036 +/- (1.332)	0.494 +/- (0.026)
			VGG-16 RAVEN	0.249 +/- (0.011)	0.092 +/- (0.010)	26.004 +/- (1.900)	0.488 +/- (0.024)
			SuperFormer	0.497 +/- (0.012)	0.101 +/- (0.011)	24.718 +/- (1.318)	0.462 +/- (0.023)
			Trilinear	0.500 +/- (0.011)	0.101 +/- (0.011)	24.861 +/- (1.524)	0.462 +/- (0.024)
	Deg. B	2×2×2	ArSSR-RDN	0.220 +/- (0.037)	0.049 +/- (0.013)	30.085 +/- (3.115)	0.818 +/- (0.030)
			ArSSR-ResCNN	0.274 +/- (0.030)	0.055 +/- (0.015)	29.172 +/- (3.117)	0.772 +/- (0.027)
			Lanczos	0.197 +/- (0.029)	0.032 +/- (0.005)	35.268 +/- (1.546)	0.872 +/- (0.023)
			NLMUPSAMPLE	0.229 +/- (0.027)	0.037 +/- (0.005)	33.935 +/- (1.625)	0.845 +/- (0.021)
			RAVEN	0.055 +/- (0.011)	0.025 +/- (0.004)	36.947 +/- (1.529)	0.919 +/- (0.015)
			4× RAVEN	0.072 +/- (0.013)	0.031 +/- (0.003)	34.740 +/- (1.271)	0.894 +/- (0.016)
			Augmented RAVEN	0.064 +/- (0.013)	0.030 +/- (0.007)	35.040 +/- (2.241)	0.902 +/- (0.016)
			In-vivo RAVEN	0.055 +/- (0.009)	0.030 +/- (0.004)	35.398 +/- (1.606)	0.909 +/- (0.014)
			L1 RAVEN	0.077 +/- (0.015)	0.025 +/- (0.005)	37.017 +/- (1.821)	0.916 +/- (0.013)
			VGG-16 RAVEN	0.055 +/- (0.010)	0.035 +/- (0.008)	34.059 +/- (2.862)	0.907 +/- (0.013)
			SuperFormer	0.198 +/- (0.013)	0.057 +/- (0.014)	29.448 +/- (2.825)	0.805 +/- (0.017)
			Trilinear	0.181 +/- (0.014)	0.040 +/- (0.006)	33.266 +/- (1.704)	0.826 +/- (0.021)
		3×3×3	ArSSR-RDN	0.350 +/- (0.034)	0.068 +/- (0.009)	27.863 +/- (1.633)	0.648 +/- (0.026)
			ArSSR-ResCNN	0.418 +/- (0.021)	0.075 +/- (0.013)	27.093 +/- (1.817)	0.605 +/- (0.023)
			Lanczos	0.427 +/- (0.024)	0.059 +/- (0.009)	29.423 +/- (1.794)	0.696 +/- (0.027)
			NLMUPSAMPLE	0.398 +/- (0.023)	0.060 +/- (0.009)	29.300 +/- (1.783)	0.692 +/- (0.026)
			RAVEN	0.191 +/- (0.026)	0.047 +/- (0.008)	31.262 +/- (2.069)	0.791 +/- (0.031)
			4× RAVEN	0.162 +/- (0.019)	0.048 +/- (0.007)	30.823 +/- (1.717)	0.795 +/- (0.022)
			Augmented RAVEN	0.176 +/- (0.023)	0.047 +/- (0.009)	31.141 +/- (1.954)	0.798 +/- (0.023)
			In-vivo RAVEN	0.167 +/- (0.025)	0.046 +/- (0.009)	31.373 +/- (1.948)	0.809 +/- (0.024)
			L1 RAVEN	0.281 +/- (0.032)	0.051 +/- (0.008)	30.688 +/- (1.774)	0.756 +/- (0.024)
			VGG-16 RAVEN	0.166 +/- (0.020)	0.049 +/- (0.010)	30.934 +/- (2.448)	0.811 +/- (0.020)
			SuperFormer	0.431 +/- (0.015)	0.086 +/- (0.018)	25.503 +/- (2.583)	0.617 +/- (0.022)
			Trilinear	0.430 +/- (0.014)	0.071 +/- (0.011)	27.655 +/- (1.825)	0.636 +/- (0.025)
		4×4×4	ArSSR-RDN	0.474 +/- (0.034)	0.092 +/- (0.014)	25.364 +/- (1.840)	0.516 +/- (0.027)

			ArSSR-ResCNN	0.504 +/- (0.026)	0.090 +/- (0.013)	25.455 +/- (1.686)	0.521 +/- (0.024)
			Lanczos	0.569 +/- (0.018)	0.102 +/- (0.012)	24.699 +/- (1.545)	0.461 +/- (0.024)
			NLMUPSAMPLE	0.568 +/- (0.021)	0.104 +/- (0.015)	24.494 +/- (1.645)	0.452 +/- (0.030)
			RAVEN	0.373 +/- (0.018)	0.102 +/- (0.013)	24.529 +/- (1.743)	0.456 +/- (0.026)
			4× RAVEN	0.263 +/- (0.021)	0.098 +/- (0.012)	25.094 +/- (1.556)	0.480 +/- (0.027)
			Augmented RAVEN	0.328 +/- (0.016)	0.101 +/- (0.012)	24.693 +/- (1.590)	0.453 +/- (0.024)
			In-vivo RAVEN	0.311 +/- (0.014)	0.100 +/- (0.013)	24.924 +/- (1.603)	0.459 +/- (0.023)
			L1 RAVEN	0.469 +/- (0.021)	0.102 +/- (0.013)	24.688 +/- (1.602)	0.457 +/- (0.024)
			VGG-16 RAVEN	0.328 +/- (0.013)	0.101 +/- (0.013)	24.736 +/- (1.917)	0.460 +/- (0.022)
			SuperFormer	0.577 +/- (0.013)	0.111 +/- (0.013)	23.668 +/- (1.372)	0.425 +/- (0.021)
			Trilinear	0.582 +/- (0.012)	0.110 +/- (0.014)	23.748 +/- (1.657)	0.425 +/- (0.021)
			ArSSR-RDN	0.178 +/- (0.034)	0.041 +/- (0.008)	31.821 +/- (2.209)	0.835 +/- (0.029)
			ArSSR-ResCNN	0.221 +/- (0.033)	0.047 +/- (0.011)	30.604 +/- (2.740)	0.805 +/- (0.026)
			Lanczos	0.147 +/- (0.028)	0.026 +/- (0.004)	36.631 +/- (1.064)	0.896 +/- (0.027)
			NLMUPSAMPLE	0.168 +/- (0.030)	0.028 +/- (0.004)	36.475 +/- (1.093)	0.887 +/- (0.023)
			RAVEN	0.054 +/- (0.012)	0.025 +/- (0.003)	36.898 +/- (0.916)	0.909 +/- (0.017)
		2×2×2	4× RAVEN	0.071 +/- (0.013)	0.030 +/- (0.003)	35.363 +/- (1.138)	0.895 +/- (0.016)
			Augmented RAVEN	0.067 +/- (0.013)	0.029 +/- (0.004)	35.696 +/- (1.056)	0.894 +/- (0.017)
			In-vivo RAVEN	0.056 +/- (0.009)	0.030 +/- (0.003)	35.037 +/- (1.172)	0.894 +/- (0.017)
			L1 RAVEN	0.071 +/- (0.014)	0.025 +/- (0.002)	37.215 +/- (0.748)	0.912 +/- (0.015)
			VGG-16 RAVEN	0.063 +/- (0.011)	0.037 +/- (0.006)	33.061 +/- (2.148)	0.887 +/- (0.017)
			SuperFormer	0.128 +/- (0.012)	0.050 +/- (0.012)	30.349 +/- (2.495)	0.842 +/- (0.021)
			Trilinear	0.115 +/- (0.014)	0.033 +/- (0.004)	34.890 +/- (1.180)	0.854 +/- (0.026)
			ArSSR-RDN	0.271 +/- (0.042)	0.062 +/- (0.009)	28.572 +/- (1.739)	0.672 +/- (0.033)
			ArSSR-ResCNN	0.308 +/- (0.032)	0.067 +/- (0.013)	27.773 +/- (2.377)	0.656 +/- (0.028)
			Lanczos	0.340 +/- (0.040)	0.043 +/- (0.005)	32.334 +/- (0.966)	0.767 +/- (0.034)
			NLMUPSAMPLE	0.272 +/- (0.038)	0.042 +/- (0.005)	32.725 +/- (0.951)	0.771 +/- (0.031)
			RAVEN	0.160 +/- (0.027)	0.038 +/- (0.005)	33.329 +/- (1.106)	0.803 +/- (0.033)
	Deg. C	3×3×3	4× RAVEN	0.155 +/- (0.019)	0.042 +/- (0.004)	32.297 +/- (1.265)	0.795 +/- (0.027)
			Augmented RAVEN	0.161 +/- (0.026)	0.041 +/- (0.005)	32.755 +/- (1.079)	0.792 +/- (0.030)
			In-vivo RAVEN	0.150 +/- (0.024)	0.041 +/- (0.005)	32.684 +/- (1.163)	0.802 +/- (0.029)
			L1 RAVEN	0.201 +/- (0.035)	0.039 +/- (0.006)	33.391 +/- (1.229)	0.802 +/- (0.031)
			VGG-16 RAVEN	0.164 +/- (0.026)	0.047 +/- (0.006)	30.970 +/- (1.604)	0.786 +/- (0.033)
			SuperFormer	0.302 +/- (0.026)	0.065 +/- (0.009)	28.166 +/- (1.875)	0.703 +/- (0.026)
			Trilinear	0.298 +/- (0.026)	0.052 +/- (0.006)	30.693 +/- (1.431)	0.717 +/- (0.030)
			ArSSR-RDN	0.309 +/- (0.053)	0.098 +/- (0.011)	25.048 +/- (1.565)	0.450 +/- (0.026)
			ArSSR-ResCNN	0.326 +/- (0.057)	0.096 +/- (0.010)	25.130 +/- (1.525)	0.458 +/- (0.022)
			Lanczos	0.459 +/- (0.030)	0.056 +/- (0.006)	29.867 +/- (1.059)	0.671 +/- (0.035)
			NLMUPSAMPLE	0.442 +/- (0.030)	0.057 +/- (0.006)	29.906 +/- (1.184)	0.669 +/- (0.033)
			RAVEN	0.215 +/- (0.022)	0.057 +/- (0.007)	29.816 +/- (1.433)	0.662 +/- (0.041)
		4×4×4	4× RAVEN	0.182 +/- (0.057)	0.057 +/- (0.037)	29.995 +/- (2.065)	0.684 +/- (0.053)
			Augmented RAVEN	0.207 +/- (0.019)	0.058 +/- (0.005)	29.601 +/- (0.963)	0.646 +/- (0.036)
			In-vivo RAVEN	0.199 +/- (0.019)	0.058 +/- (0.006)	29.653 +/- (1.089)	0.658 +/- (0.034)
			L1 RAVEN	0.265 +/- (0.034)	0.055 +/- (0.007)	30.255 +/- (1.171)	0.675 +/- (0.035)
			VGG-16 RAVEN	0.267 +/- (0.051)	0.069 +/- (0.020)	27.702 +/- (1.954)	0.617 +/- (0.054)
			SuperFormer	0.426 +/- (0.019)	0.071 +/- (0.008)	27.605 +/- (1.293)	0.610 +/- (0.029)
			Trilinear	0.425 +/- (0.016)	0.068 +/- (0.008)	28.119 +/- (1.488)	0.612 +/- (0.029)
			ArSSR-RDN	0.169 +/- (0.032)	0.064 +/- (0.019)	27.758 +/- (3.888)	0.773 +/- (0.035)
			ArSSR-ResCNN	0.193 +/- (0.006)	0.069 +/- (0.035)	27.284 +/- (5.842)	0.772 +/- (0.048)
			Lanczos	0.094 +/- (0.049)	0.029 +/- (0.017)	35.775 +/- (7.588)	0.940 +/- (0.034)
LUSEBRINK	Deg. A	2×2×2	NLMUPSAMPLE	0.117 +/- (0.042)	0.040 +/- (0.023)	32.609 +/- (7.365)	0.925 +/- (0.033)
			RAVEN	0.054 +/- (0.010)	0.033 +/- (0.006)	33.748 +/- (1.692)	0.917 +/- (0.006)
			4× RAVEN	0.073 +/- (0.013)	0.039 +/- (0.004)	32.558 +/- (0.480)	0.892 +/- (0.007)
			Augmented RAVEN	0.069 +/- (0.016)	0.032 +/- (0.009)	34.635 +/- (2.598)	0.900 +/- (0.008)
			In-vivo RAVEN	0.062 +/- (0.016)	0.037 +/- (0.008)	33.146 +/- (2.721)	0.902 +/- (0.010)

			L1 RAVEN	0.072 +/- (0.016)	0.028 +/- (0.005)	35.098 +/- (0.909)	0.925 +/- (0.008)
			VGG-16 RAVEN	0.066 +/- (0.023)	0.042 +/- (0.005)	31.392 +/- (1.214)	0.897 +/- (0.017)
			SuperFormer	0.125 +/- (0.009)	0.067 +/- (0.015)	27.114 +/- (2.582)	0.869 +/- (0.008)
			Trilinear	0.105 +/- (0.017)	0.045 +/- (0.025)	31.701 +/- (6.892)	0.909 +/- (0.038)
		3×3×3	ArSSR-RDN	0.217 +/- (0.037)	0.103 +/- (0.052)	24.323 +/- (7.021)	0.627 +/- (0.120)
			ArSSR-ResCNN	0.230 +/- (0.023)	0.106 +/- (0.052)	23.952 +/- (6.777)	0.618 +/- (0.116)
			Lanczos	0.264 +/- (0.066)	0.060 +/- (0.031)	28.936 +/- (6.166)	0.816 +/- (0.067)
			NLMUPSAMPLE	0.217 +/- (0.050)	0.060 +/- (0.031)	28.880 +/- (6.210)	0.818 +/- (0.063)
			RAVEN	0.135 +/- (0.033)	0.062 +/- (0.027)	27.703 +/- (4.425)	0.847 +/- (0.040)
			4× RAVEN	0.139 +/- (0.033)	0.061 +/- (0.018)	27.901 +/- (3.097)	0.822 +/- (0.048)
			Augmented RAVEN	0.127 +/- (0.037)	0.060 +/- (0.026)	28.123 +/- (4.956)	0.838 +/- (0.046)
			In-vivo RAVEN	0.130 +/- (0.031)	0.055 +/- (0.020)	29.271 +/- (4.212)	0.841 +/- (0.035)
			L1 RAVEN	0.195 +/- (0.026)	0.064 +/- (0.029)	27.655 +/- (5.078)	0.833 +/- (0.049)
			VGG-16 RAVEN	0.120 +/- (0.042)	0.056 +/- (0.011)	28.755 +/- (2.322)	0.849 +/- (0.031)
			SuperFormer	0.312 +/- (0.011)	0.088 +/- (0.015)	24.790 +/- (1.737)	0.714 +/- (0.049)
			Trilinear	0.289 +/- (0.040)	0.072 +/- (0.032)	26.992 +/- (5.210)	0.753 +/- (0.079)
		4×4×4	ArSSR-RDN	0.309 +/- (0.018)	0.088 +/- (0.040)	25.452 +/- (5.729)	0.632 +/- (0.117)
			ArSSR-ResCNN	0.328 +/- (0.016)	0.104 +/- (0.057)	23.954 +/- (7.279)	0.617 +/- (0.127)
			Lanczos	0.449 +/- (0.065)	0.112 +/- (0.041)	23.643 +/- (4.380)	0.503 +/- (0.131)
			NLMUPSAMPLE	0.446 +/- (0.061)	0.112 +/- (0.041)	23.538 +/- (4.322)	0.502 +/- (0.132)
			RAVEN	0.268 +/- (0.028)	0.119 +/- (0.037)	22.674 +/- (3.318)	0.481 +/- (0.124)
			4× RAVEN	0.233 +/- (0.021)	0.111 +/- (0.032)	23.450 +/- (2.538)	0.478 +/- (0.113)
			Augmented RAVEN	0.248 +/- (0.031)	0.113 +/- (0.037)	23.388 +/- (3.587)	0.478 +/- (0.119)
			In-vivo RAVEN	0.249 +/- (0.029)	0.113 +/- (0.036)	23.368 +/- (3.305)	0.477 +/- (0.119)
			L1 RAVEN	0.319 +/- (0.028)	0.116 +/- (0.039)	23.082 +/- (3.813)	0.493 +/- (0.127)
			VGG-16 RAVEN	0.263 +/- (0.041)	0.113 +/- (0.030)	23.181 +/- (2.383)	0.479 +/- (0.114)
			SuperFormer	0.473 +/- (0.039)	0.121 +/- (0.022)	22.396 +/- (1.812)	0.468 +/- (0.127)
			Trilinear	0.488 +/- (0.048)	0.116 +/- (0.040)	23.027 +/- (3.902)	0.478 +/- (0.139)
	Deg. B	2×2×2	ArSSR-RDN	0.187 +/- (0.022)	0.067 +/- (0.028)	27.459 +/- (5.318)	0.773 +/- (0.021)
			ArSSR-ResCNN	0.219 +/- (0.004)	0.074 +/- (0.038)	26.757 +/- (6.201)	0.757 +/- (0.050)
			Lanczos	0.132 +/- (0.050)	0.042 +/- (0.024)	32.258 +/- (7.352)	0.924 +/- (0.036)
			NLMUPSAMPLE	0.167 +/- (0.045)	0.050 +/- (0.028)	30.597 +/- (6.783)	0.895 +/- (0.042)
			RAVEN	0.058 +/- (0.004)	0.035 +/- (0.008)	32.979 +/- (3.014)	0.921 +/- (0.007)
			4× RAVEN	0.076 +/- (0.009)	0.036 +/- (0.008)	33.372 +/- (2.223)	0.894 +/- (0.009)
			Augmented RAVEN	0.068 +/- (0.009)	0.032 +/- (0.012)	34.515 +/- (3.715)	0.907 +/- (0.010)
			In-vivo RAVEN	0.062 +/- (0.011)	0.035 +/- (0.007)	33.300 +/- (2.016)	0.909 +/- (0.008)
			L1 RAVEN	0.080 +/- (0.008)	0.028 +/- (0.010)	35.218 +/- (3.409)	0.929 +/- (0.010)
			VGG-16 RAVEN	0.060 +/- (0.011)	0.038 +/- (0.006)	32.467 +/- (1.473)	0.911 +/- (0.011)
			SuperFormer	0.177 +/- (0.017)	0.073 +/- (0.018)	26.339 +/- (2.618)	0.840 +/- (0.015)
			Trilinear	0.152 +/- (0.019)	0.053 +/- (0.029)	29.908 +/- (6.421)	0.879 +/- (0.045)
		3×3×3	ArSSR-RDN	0.292 +/- (0.037)	0.104 +/- (0.050)	24.018 +/- (6.202)	0.615 +/- (0.114)
			ArSSR-ResCNN	0.317 +/- (0.034)	0.110 +/- (0.053)	23.427 +/- (6.268)	0.595 +/- (0.120)
			Lanczos	0.354 +/- (0.061)	0.072 +/- (0.033)	26.979 +/- (5.287)	0.754 +/- (0.079)
			NLMUPSAMPLE	0.324 +/- (0.052)	0.073 +/- (0.033)	26.896 +/- (5.206)	0.745 +/- (0.081)
			RAVEN	0.200 +/- (0.030)	0.073 +/- (0.029)	26.115 +/- (3.952)	0.793 +/- (0.057)
			4× RAVEN	0.184 +/- (0.033)	0.071 +/- (0.022)	26.535 +/- (3.140)	0.780 +/- (0.065)
			Augmented RAVEN	0.181 +/- (0.036)	0.072 +/- (0.031)	26.439 +/- (4.704)	0.792 +/- (0.059)
			In-vivo RAVEN	0.181 +/- (0.030)	0.064 +/- (0.024)	27.854 +/- (3.974)	0.798 +/- (0.049)
			L1 RAVEN	0.281 +/- (0.034)	0.075 +/- (0.032)	26.012 +/- (4.420)	0.770 +/- (0.064)
			VGG-16 RAVEN	0.177 +/- (0.043)	0.067 +/- (0.015)	27.002 +/- (2.305)	0.801 +/- (0.046)
			SuperFormer	0.409 +/- (0.020)	0.090 +/- (0.016)	24.767 +/- (2.276)	0.645 +/- (0.068)
			Trilinear	0.398 +/- (0.047)	0.082 +/- (0.033)	25.778 +/- (4.614)	0.685 +/- (0.094)
		4×4×4	ArSSR-RDN	0.423 +/- (0.015)	0.096 +/- (0.038)	24.685 +/- (4.267)	0.569 +/- (0.133)
			ArSSR-ResCNN	0.460 +/- (0.015)	0.103 +/- (0.044)	23.907 +/- (5.036)	0.564 +/- (0.126)
			Lanczos	0.543 +/- (0.053)	0.116 +/- (0.040)	22.995 +/- (3.820)	0.479 +/- (0.139)

			NLMUPSAMPLE	0.543 +/- (0.051)	0.117 +/- (0.040)	22.934 +/- (3.758)	0.473 +/- (0.141)
			RAVEN	0.368 +/- (0.014)	0.122 +/- (0.034)	22.171 +/- (2.722)	0.462 +/- (0.133)
			4× RAVEN	0.293 +/- (0.014)	0.119 +/- (0.030)	22.446 +/- (2.161)	0.472 +/- (0.121)
			Augmented RAVEN	0.333 +/- (0.014)	0.121 +/- (0.037)	22.336 +/- (3.126)	0.460 +/- (0.130)
			In-vivo RAVEN	0.331 +/- (0.016)	0.118 +/- (0.034)	22.688 +/- (2.705)	0.460 +/- (0.128)
			L1 RAVEN	0.449 +/- (0.037)	0.120 +/- (0.038)	22.443 +/- (3.228)	0.469 +/- (0.135)
			VGG-16 RAVEN	0.339 +/- (0.019)	0.120 +/- (0.028)	22.322 +/- (1.952)	0.459 +/- (0.125)
			SuperFormer	0.571 +/- (0.034)	0.121 +/- (0.026)	22.147 +/- (1.616)	0.438 +/- (0.136)
			Trilinear	0.591 +/- (0.044)	0.120 +/- (0.040)	22.601 +/- (3.507)	0.448 +/- (0.148)
			ArSSR-RDN	0.166 +/- (0.033)	0.061 +/- (0.014)	28.202 +/- (3.124)	0.768 +/- (0.040)
			ArSSR-ResCNN	0.183 +/- (0.010)	0.067 +/- (0.032)	27.617 +/- (5.498)	0.776 +/- (0.049)
			Lanczos	0.090 +/- (0.053)	0.024 +/- (0.014)	37.539 +/- (7.320)	0.941 +/- (0.033)
			NLMUPSAMPLE	0.106 +/- (0.050)	0.034 +/- (0.020)	34.154 +/- (7.650)	0.932 +/- (0.033)
			RAVEN	0.055 +/- (0.011)	0.032 +/- (0.003)	33.799 +/- (0.840)	0.913 +/- (0.005)
		2×2×2	4× RAVEN	0.075 +/- (0.013)	0.039 +/- (0.004)	32.339 +/- (0.402)	0.892 +/- (0.007)
			Augmented RAVEN	0.073 +/- (0.016)	0.032 +/- (0.008)	34.306 +/- (1.910)	0.895 +/- (0.007)
			In-vivo RAVEN	0.062 +/- (0.016)	0.037 +/- (0.009)	32.838 +/- (2.758)	0.899 +/- (0.010)
			L1 RAVEN	0.077 +/- (0.015)	0.031 +/- (0.002)	33.926 +/- (0.764)	0.922 +/- (0.005)
			VGG-16 RAVEN	0.075 +/- (0.023)	0.045 +/- (0.004)	30.565 +/- (0.839)	0.885 +/- (0.020)
			SuperFormer	0.110 +/- (0.004)	0.066 +/- (0.012)	27.204 +/- (2.133)	0.873 +/- (0.013)
			Trilinear	0.092 +/- (0.020)	0.041 +/- (0.023)	32.504 +/- (6.991)	0.907 +/- (0.042)
			ArSSR-RDN	0.190 +/- (0.046)	0.101 +/- (0.049)	24.750 +/- (6.757)	0.609 +/- (0.111)
			ArSSR-ResCNN	0.195 +/- (0.038)	0.103 +/- (0.049)	24.614 +/- (6.737)	0.612 +/- (0.110)
			Lanczos	0.240 +/- (0.086)	0.049 +/- (0.024)	30.854 +/- (6.126)	0.826 +/- (0.060)
			NLMUPSAMPLE	0.177 +/- (0.065)	0.048 +/- (0.024)	31.080 +/- (6.198)	0.825 +/- (0.056)
			RAVEN	0.135 +/- (0.043)	0.057 +/- (0.024)	28.895 +/- (4.609)	0.823 +/- (0.036)
	Deg. C	3×3×3	4× RAVEN	0.139 +/- (0.042)	0.055 +/- (0.014)	29.008 +/- (2.757)	0.804 +/- (0.039)
			Augmented RAVEN	0.139 +/- (0.039)	0.055 +/- (0.022)	29.266 +/- (4.617)	0.802 +/- (0.037)
			In-vivo RAVEN	0.133 +/- (0.041)	0.052 +/- (0.017)	29.924 +/- (3.794)	0.816 +/- (0.034)
			L1 RAVEN	0.176 +/- (0.036)	0.052 +/- (0.021)	29.855 +/- (4.893)	0.829 +/- (0.045)
			VGG-16 RAVEN	0.139 +/- (0.041)	0.053 +/- (0.010)	29.489 +/- (1.699)	0.801 +/- (0.023)
			SuperFormer	0.255 +/- (0.018)	0.082 +/- (0.012)	25.237 +/- (1.665)	0.745 +/- (0.039)
			Trilinear	0.233 +/- (0.053)	0.064 +/- (0.029)	28.241 +/- (5.380)	0.775 +/- (0.074)
			ArSSR-RDN	0.280 +/- (0.022)	0.114 +/- (0.048)	23.867 +/- (5.476)	0.502 +/- (0.131)
			ArSSR-ResCNN	0.298 +/- (0.021)	0.117 +/- (0.049)	23.586 +/- (5.537)	0.497 +/- (0.130)
			Lanczos	0.369 +/- (0.079)	0.070 +/- (0.031)	27.525 +/- (5.305)	0.703 +/- (0.084)
			NLMUPSAMPLE	0.352 +/- (0.076)	0.070 +/- (0.032)	27.489 +/- (5.450)	0.703 +/- (0.089)
			RAVEN	0.207 +/- (0.044)	0.084 +/- (0.032)	25.265 +/- (4.127)	0.664 +/- (0.072)
		4×4×4	4× RAVEN	0.191 +/- (0.029)	0.075 +/- (0.022)	26.419 +/- (2.853)	0.653 +/- (0.062)
			Augmented RAVEN	0.205 +/- (0.033)	0.077 +/- (0.027)	26.421 +/- (3.883)	0.638 +/- (0.066)
			In-vivo RAVEN	0.204 +/- (0.042)	0.076 +/- (0.028)	26.494 +/- (4.112)	0.655 +/- (0.067)
			L1 RAVEN	0.254 +/- (0.050)	0.085 +/- (0.033)	25.181 +/- (4.492)	0.671 +/- (0.086)
			VGG-16 RAVEN	0.255 +/- (0.046)	0.081 +/- (0.026)	25.815 +/- (2.838)	0.613 +/- (0.068)
			SuperFormer	0.377 +/- (0.047)	0.108 +/- (0.024)	23.021 +/- (2.386)	0.613 +/- (0.096)
			Trilinear	0.386 +/- (0.057)	0.081 +/- (0.033)	25.992 +/- (4.577)	0.650 +/- (0.104)
			ArSSR-RDN	0.247 +/- (0.026)	0.069 +/- (0.005)	27.517 +/- (1.110)	0.640 +/- (0.026)
			ArSSR-ResCNN	0.343 +/- (0.038)	0.060 +/- (0.010)	28.478 +/- (2.077)	0.693 +/- (0.021)
			Lanczos	0.212 +/- (0.018)	0.043 +/- (0.011)	31.625 +/- (2.979)	0.812 +/- (0.024)
			NLMUPSAMPLE	0.253 +/- (0.027)	0.048 +/- (0.013)	30.493 +/- (2.886)	0.792 +/- (0.026)
OSCBS	Deg. A	2×2×2	RAVEN	0.135 +/- (0.025)	0.052 +/- (0.015)	29.678 +/- (3.054)	0.783 +/- (0.029)
			4× RAVEN	0.180 +/- (0.031)	0.051 +/- (0.015)	29.963 +/- (3.112)	0.782 +/- (0.029)
			Augmented RAVEN	0.165 +/- (0.030)	0.053 +/- (0.017)	29.585 +/- (3.386)	0.780 +/- (0.032)
			In-vivo RAVEN	0.136 +/- (0.029)	0.050 +/- (0.013)	30.090 +/- (2.667)	0.783 +/- (0.028)
			L1 RAVEN	0.194 +/- (0.030)	0.049 +/- (0.015)	30.205 +/- (3.122)	0.792 +/- (0.031)
			VGG-16 RAVEN	0.124 +/- (0.023)	0.050 +/- (0.011)	30.192 +/- (2.307)	0.789 +/- (0.029)

			SuperFormer	0.177 +/- (0.019)	0.049 +/- (0.008)	30.521 +/- (1.870)	0.776 +/- (0.020)
			Trilinear	0.167 +/- (0.017)	0.049 +/- (0.012)	30.423 +/- (2.813)	0.785 +/- (0.020)
		3×3×3	ArSSR-RDN	0.335 +/- (0.042)	0.085 +/- (0.005)	25.757 +/- (1.158)	0.509 +/- (0.014)
			ArSSR-ResCNN	0.475 +/- (0.045)	0.078 +/- (0.007)	26.264 +/- (1.380)	0.545 +/- (0.015)
			Lanczos	0.452 +/- (0.039)	0.062 +/- (0.010)	28.360 +/- (1.949)	0.652 +/- (0.026)
			NLMUPSAMPLE	0.403 +/- (0.042)	0.061 +/- (0.011)	28.442 +/- (2.062)	0.659 +/- (0.029)
			RAVEN	0.289 +/- (0.045)	0.063 +/- (0.016)	27.945 +/- (2.776)	0.693 +/- (0.037)
			4× RAVEN	0.278 +/- (0.043)	0.063 +/- (0.016)	28.056 +/- (2.932)	0.688 +/- (0.035)
			Augmented RAVEN	0.292 +/- (0.044)	0.064 +/- (0.017)	27.861 +/- (3.028)	0.694 +/- (0.037)
			In-vivo RAVEN	0.281 +/- (0.045)	0.062 +/- (0.015)	28.154 +/- (2.653)	0.699 +/- (0.035)
			L1 RAVEN	0.366 +/- (0.051)	0.061 +/- (0.015)	28.274 +/- (2.735)	0.683 +/- (0.036)
			VGG-16 RAVEN	0.249 +/- (0.040)	0.058 +/- (0.012)	28.835 +/- (2.260)	0.717 +/- (0.038)
			SuperFormer	0.445 +/- (0.036)	0.069 +/- (0.005)	27.489 +/- (1.062)	0.593 +/- (0.020)
			Trilinear	0.451 +/- (0.037)	0.069 +/- (0.008)	27.433 +/- (1.476)	0.598 +/- (0.020)
		4×4×4	ArSSR-RDN	0.496 +/- (0.046)	0.091 +/- (0.009)	24.888 +/- (1.485)	0.482 +/- (0.013)
			ArSSR-ResCNN	0.513 +/- (0.047)	0.090 +/- (0.008)	24.958 +/- (1.401)	0.482 +/- (0.012)
			Lanczos	0.624 +/- (0.041)	0.090 +/- (0.005)	25.308 +/- (0.981)	0.473 +/- (0.014)
			NLMUPSAMPLE	0.616 +/- (0.042)	0.090 +/- (0.005)	25.286 +/- (0.953)	0.473 +/- (0.014)
			RAVEN	0.371 +/- (0.046)	0.092 +/- (0.008)	25.003 +/- (1.325)	0.485 +/- (0.016)
			4× RAVEN	0.317 +/- (0.048)	0.091 +/- (0.011)	25.242 +/- (1.826)	0.500 +/- (0.022)
			Augmented RAVEN	0.343 +/- (0.044)	0.093 +/- (0.010)	24.941 +/- (1.663)	0.487 +/- (0.018)
			In-vivo RAVEN	0.321 +/- (0.044)	0.092 +/- (0.009)	25.062 +/- (1.549)	0.491 +/- (0.017)
			L1 RAVEN	0.464 +/- (0.052)	0.090 +/- (0.008)	25.187 +/- (1.384)	0.486 +/- (0.017)
			VGG-16 RAVEN	0.305 +/- (0.037)	0.088 +/- (0.004)	25.542 +/- (0.860)	0.492 +/- (0.017)
			SuperFormer	0.625 +/- (0.039)	0.093 +/- (0.004)	24.919 +/- (0.645)	0.442 +/- (0.012)
			Trilinear	0.634 +/- (0.039)	0.094 +/- (0.004)	24.861 +/- (0.711)	0.442 +/- (0.013)
	Deg. B	2×2×2	ArSSR-RDN	0.285 +/- (0.033)	0.069 +/- (0.005)	27.437 +/- (0.967)	0.637 +/- (0.020)
			ArSSR-ResCNN	0.404 +/- (0.043)	0.063 +/- (0.010)	27.993 +/- (2.050)	0.666 +/- (0.020)
			Lanczos	0.275 +/- (0.025)	0.049 +/- (0.013)	30.283 +/- (2.825)	0.790 +/- (0.023)
			NLMUPSAMPLE	0.336 +/- (0.035)	0.053 +/- (0.012)	29.526 +/- (2.502)	0.748 +/- (0.026)
			RAVEN	0.169 +/- (0.030)	0.053 +/- (0.017)	29.400 +/- (3.230)	0.792 +/- (0.029)
			4× RAVEN	0.204 +/- (0.035)	0.053 +/- (0.016)	29.556 +/- (3.323)	0.784 +/- (0.029)
			Augmented RAVEN	0.195 +/- (0.033)	0.054 +/- (0.018)	29.249 +/- (3.589)	0.786 +/- (0.032)
			In-vivo RAVEN	0.167 +/- (0.033)	0.051 +/- (0.015)	29.895 +/- (2.843)	0.794 +/- (0.028)
			L1 RAVEN	0.238 +/- (0.035)	0.050 +/- (0.015)	29.984 +/- (3.202)	0.792 +/- (0.031)
			VGG-16 RAVEN	0.148 +/- (0.026)	0.049 +/- (0.012)	30.290 +/- (2.458)	0.807 +/- (0.028)
			SuperFormer	0.276 +/- (0.030)	0.055 +/- (0.008)	29.397 +/- (1.750)	0.732 +/- (0.022)
			Trilinear	0.269 +/- (0.029)	0.055 +/- (0.011)	29.311 +/- (2.349)	0.739 +/- (0.022)
		3×3×3	ArSSR-RDN	0.418 +/- (0.042)	0.086 +/- (0.006)	25.524 +/- (1.228)	0.496 +/- (0.012)
			ArSSR-ResCNN	0.559 +/- (0.046)	0.083 +/- (0.007)	25.745 +/- (1.297)	0.505 +/- (0.013)
			Lanczos	0.551 +/- (0.042)	0.069 +/- (0.008)	27.391 +/- (1.451)	0.592 +/- (0.021)
			NLMUPSAMPLE	0.525 +/- (0.043)	0.069 +/- (0.008)	27.373 +/- (1.459)	0.589 +/- (0.022)
			RAVEN	0.372 +/- (0.052)	0.068 +/- (0.014)	27.358 +/- (2.313)	0.641 +/- (0.031)
			4× RAVEN	0.327 +/- (0.049)	0.068 +/- (0.016)	27.330 +/- (2.759)	0.650 +/- (0.032)
			Augmented RAVEN	0.355 +/- (0.049)	0.068 +/- (0.015)	27.306 +/- (2.627)	0.651 +/- (0.031)
			In-vivo RAVEN	0.345 +/- (0.050)	0.066 +/- (0.014)	27.527 +/- (2.417)	0.658 +/- (0.031)
			L1 RAVEN	0.463 +/- (0.054)	0.068 +/- (0.013)	27.385 +/- (2.207)	0.617 +/- (0.030)
			VGG-16 RAVEN	0.316 +/- (0.046)	0.061 +/- (0.009)	28.381 +/- (1.593)	0.679 +/- (0.034)
			SuperFormer	0.557 +/- (0.039)	0.076 +/- (0.004)	26.646 +/- (0.775)	0.534 +/- (0.016)
			Trilinear	0.570 +/- (0.041)	0.076 +/- (0.006)	26.629 +/- (1.092)	0.540 +/- (0.016)
		4×4×4	ArSSR-RDN	0.578 +/- (0.036)	0.096 +/- (0.008)	24.549 +/- (1.360)	0.441 +/- (0.013)
			ArSSR-ResCNN	0.606 +/- (0.039)	0.096 +/- (0.007)	24.396 +/- (1.127)	0.434 +/- (0.010)
			Lanczos	0.684 +/- (0.040)	0.094 +/- (0.004)	24.787 +/- (0.677)	0.436 +/- (0.013)
			NLMUPSAMPLE	0.681 +/- (0.040)	0.095 +/- (0.004)	24.720 +/- (0.652)	0.431 +/- (0.013)
			RAVEN	0.503 +/- (0.050)	0.096 +/- (0.006)	24.621 +/- (0.930)	0.441 +/- (0.013)

			4× RAVEN	0.391 +/- (0.052)	0.094 +/- (0.010)	24.790 +/- (1.579)	0.469 +/- (0.016)
			Augmented RAVEN	0.459 +/- (0.047)	0.097 +/- (0.007)	24.577 +/- (1.226)	0.444 +/- (0.014)
			In-vivo RAVEN	0.422 +/- (0.045)	0.097 +/- (0.008)	24.537 +/- (1.267)	0.446 +/- (0.013)
			L1 RAVEN	0.599 +/- (0.047)	0.095 +/- (0.006)	24.683 +/- (0.967)	0.438 +/- (0.013)
			VGG-16 RAVEN	0.427 +/- (0.042)	0.093 +/- (0.003)	24.993 +/- (0.528)	0.450 +/- (0.013)
			SuperFormer	0.691 +/- (0.038)	0.099 +/- (0.005)	24.334 +/- (0.478)	0.405 +/- (0.011)
			Trilinear	0.703 +/- (0.040)	0.099 +/- (0.004)	24.312 +/- (0.508)	0.406 +/- (0.012)
			ArSSR-RDN	0.248 +/- (0.027)	0.069 +/- (0.004)	27.589 +/- (0.872)	0.644 +/- (0.023)
			ArSSR-ResCNN	0.341 +/- (0.041)	0.059 +/- (0.010)	28.667 +/- (2.078)	0.703 +/- (0.021)
			Lanczos	0.231 +/- (0.021)	0.041 +/- (0.009)	32.001 +/- (2.482)	0.830 +/- (0.021)
			NLMUPSAMPLE	0.264 +/- (0.028)	0.045 +/- (0.011)	30.980 +/- (2.493)	0.800 +/- (0.025)
			RAVEN	0.161 +/- (0.025)	0.048 +/- (0.012)	30.316 +/- (2.430)	0.803 +/- (0.024)
		2×2×2	4× RAVEN	0.200 +/- (0.032)	0.048 +/- (0.011)	30.573 +/- (2.408)	0.796 +/- (0.024)
			Augmented RAVEN	0.186 +/- (0.029)	0.049 +/- (0.014)	30.247 +/- (2.973)	0.799 +/- (0.027)
			In-vivo RAVEN	0.152 +/- (0.028)	0.048 +/- (0.011)	30.431 +/- (2.321)	0.799 +/- (0.024)
			L1 RAVEN	0.212 +/- (0.030)	0.046 +/- (0.012)	30.757 +/- (2.707)	0.809 +/- (0.026)
			VGG-16 RAVEN	0.153 +/- (0.021)	0.048 +/- (0.007)	30.450 +/- (1.362)	0.803 +/- (0.023)
			SuperFormer	0.177 +/- (0.022)	0.048 +/- (0.005)	30.578 +/- (1.288)	0.775 +/- (0.021)
			Trilinear	0.167 +/- (0.019)	0.049 +/- (0.010)	30.459 +/- (2.334)	0.778 +/- (0.021)
			ArSSR-RDN	0.303 +/- (0.043)	0.086 +/- (0.002)	25.708 +/- (0.494)	0.496 +/- (0.012)
			ArSSR-ResCNN	0.421 +/- (0.046)	0.076 +/- (0.006)	26.551 +/- (1.301)	0.561 +/- (0.016)
			Lanczos	0.424 +/- (0.038)	0.056 +/- (0.007)	29.270 +/- (1.620)	0.678 +/- (0.026)
			NLMUPSAMPLE	0.358 +/- (0.040)	0.056 +/- (0.008)	29.304 +/- (1.760)	0.680 +/- (0.029)
			RAVEN	0.291 +/- (0.039)	0.061 +/- (0.012)	28.475 +/- (2.180)	0.674 +/- (0.031)
	Deg. C	3×3×3	4× RAVEN	0.287 +/- (0.040)	0.060 +/- (0.010)	28.713 +/- (1.996)	0.669 +/- (0.029)
			Augmented RAVEN	0.289 +/- (0.039)	0.061 +/- (0.013)	28.448 +/- (2.407)	0.673 +/- (0.031)
			In-vivo RAVEN	0.265 +/- (0.040)	0.061 +/- (0.011)	28.495 +/- (2.068)	0.673 +/- (0.029)
			L1 RAVEN	0.338 +/- (0.047)	0.059 +/- (0.011)	28.766 +/- (2.213)	0.677 +/- (0.032)
			VGG-16 RAVEN	0.262 +/- (0.034)	0.058 +/- (0.005)	28.875 +/- (1.022)	0.676 +/- (0.028)
			SuperFormer	0.379 +/- (0.032)	0.065 +/- (0.005)	27.987 +/- (1.057)	0.617 +/- (0.021)
			Trilinear	0.384 +/- (0.032)	0.064 +/- (0.007)	28.054 +/- (1.522)	0.621 +/- (0.022)
			ArSSR-RDN	0.492 +/- (0.049)	0.121 +/- (0.004)	23.111 +/- (0.987)	0.341 +/- (0.010)
			ArSSR-ResCNN	0.504 +/- (0.049)	0.122 +/- (0.004)	22.980 +/- (1.012)	0.339 +/- (0.011)
			Lanczos	0.572 +/- (0.041)	0.080 +/- (0.006)	26.294 +/- (1.204)	0.525 +/- (0.019)
			NLMUPSAMPLE	0.562 +/- (0.040)	0.080 +/- (0.007)	26.293 +/- (1.310)	0.526 +/- (0.019)
			RAVEN	0.360 +/- (0.045)	0.104 +/- (0.018)	23.350 +/- (2.354)	0.512 +/- (0.023)
		4×4×4	4× RAVEN	0.317 +/- (0.047)	0.102 +/- (0.015)	23.790 +/- (2.039)	0.494 +/- (0.025)
			Augmented RAVEN	0.305 +/- (0.040)	0.106 +/- (0.017)	23.514 +/- (2.190)	0.492 +/- (0.023)
			In-vivo RAVEN	0.307 +/- (0.042)	0.105 +/- (0.017)	23.499 +/- (2.276)	0.495 +/- (0.022)
			L1 RAVEN	0.411 +/- (0.043)	0.116 +/- (0.020)	21.859 +/- (2.049)	0.510 +/- (0.023)
			VGG-16 RAVEN	0.308 +/- (0.025)	0.086 +/- (0.003)	25.704 +/- (0.503)	0.483 +/- (0.017)
			SuperFormer	0.570 +/- (0.035)	0.114 +/- (0.006)	22.006 +/- (0.684)	0.475 +/- (0.017)
			Trilinear	0.561 +/- (0.038)	0.084 +/- (0.006)	25.815 +/- (1.115)	0.490 +/- (0.016)

Table S4: Performance of tested methods per target voxel size, degradation type and upsampling factor (scale). Metrics reported as "mean ± (standard deviation)".

Voxel Size	Degra-dation	Scale	Upsampling Method	LPIPS ↓	MAE ↓	PSNR ↑	SSIM ↑
<= 0.60 mm	Deg. A	2×2×2	ArSSR-RDN	0.192 +/- (0.052)	0.057 +/- (0.020)	29.624 +/- (4.805)	0.763 +/- (0.107)
			ArSSR-ResCNN	0.241 +/- (0.082)	0.060 +/- (0.021)	28.099 +/- (3.488)	0.773 +/- (0.081)
			Lanczos	0.119 +/- (0.083)	0.029 +/- (0.016)	37.310 +/- (8.242)	0.902 +/- (0.078)
			NLMUPSAMPLE	0.146 +/- (0.091)	0.035 +/- (0.019)	35.440 +/- (7.464)	0.891 +/- (0.082)
			RAVEN	0.086 +/- (0.046)	0.041 +/- (0.020)	32.276 +/- (4.929)	0.882 +/- (0.077)
			4× RAVEN	0.121 +/- (0.060)	0.044 +/- (0.018)	31.724 +/- (4.456)	0.862 +/- (0.062)
			Augmented RAVEN	0.109 +/- (0.056)	0.040 +/- (0.017)	32.397 +/- (4.381)	0.869 +/- (0.070)
			In-vivo RAVEN	0.098 +/- (0.046)	0.045 +/- (0.016)	31.047 +/- (3.859)	0.867 +/- (0.066)
			L1 RAVEN	0.109 +/- (0.068)	0.037 +/- (0.016)	33.166 +/- (4.537)	0.889 +/- (0.076)
			VGG-16 RAVEN	0.095 +/- (0.043)	0.044 +/- (0.012)	31.365 +/- (2.955)	0.870 +/- (0.065)
			SuperFormer	0.159 +/- (0.031)	0.079 +/- (0.031)	25.808 +/- (4.740)	0.829 +/- (0.052)
			Trilinear	0.122 +/- (0.045)	0.038 +/- (0.019)	34.684 +/- (6.961)	0.877 +/- (0.079)
		3×3×3	ArSSR-RDN	0.247 +/- (0.094)	0.073 +/- (0.034)	28.137 +/- (5.762)	0.660 +/- (0.139)
			ArSSR-ResCNN	0.313 +/- (0.143)	0.072 +/- (0.034)	28.145 +/- (5.659)	0.660 +/- (0.122)
			Lanczos	0.303 +/- (0.144)	0.050 +/- (0.022)	32.032 +/- (6.377)	0.787 +/- (0.120)
			NLMUPSAMPLE	0.261 +/- (0.129)	0.049 +/- (0.022)	32.054 +/- (6.105)	0.793 +/- (0.116)
			RAVEN	0.165 +/- (0.100)	0.054 +/- (0.023)	29.568 +/- (5.057)	0.828 +/- (0.109)
			4× RAVEN	0.172 +/- (0.086)	0.055 +/- (0.021)	29.546 +/- (4.521)	0.813 +/- (0.099)
			Augmented RAVEN	0.169 +/- (0.098)	0.054 +/- (0.020)	29.192 +/- (4.268)	0.823 +/- (0.103)
			In-vivo RAVEN	0.168 +/- (0.090)	0.054 +/- (0.020)	29.506 +/- (4.502)	0.821 +/- (0.097)
			L1 RAVEN	0.221 +/- (0.120)	0.051 +/- (0.022)	30.258 +/- (4.961)	0.817 +/- (0.111)
			VGG-16 RAVEN	0.148 +/- (0.081)	0.049 +/- (0.012)	30.352 +/- (2.444)	0.837 +/- (0.096)
			SuperFormer	0.363 +/- (0.080)	0.093 +/- (0.026)	24.118 +/- (3.586)	0.693 +/- (0.094)
			Trilinear	0.333 +/- (0.113)	0.058 +/- (0.023)	30.125 +/- (5.020)	0.736 +/- (0.125)
		4×4×4	ArSSR-RDN	0.374 +/- (0.114)	0.075 +/- (0.025)	27.170 +/- (3.447)	0.630 +/- (0.136)
			ArSSR-ResCNN	0.385 +/- (0.114)	0.078 +/- (0.036)	27.102 +/- (4.847)	0.629 +/- (0.141)
			Lanczos	0.478 +/- (0.141)	0.085 +/- (0.022)	26.199 +/- (2.537)	0.548 +/- (0.115)
			NLMUPSAMPLE	0.474 +/- (0.135)	0.086 +/- (0.022)	26.172 +/- (2.529)	0.549 +/- (0.115)
			RAVEN	0.291 +/- (0.071)	0.095 +/- (0.021)	24.728 +/- (2.698)	0.542 +/- (0.106)
			4× RAVEN	0.246 +/- (0.062)	0.095 +/- (0.019)	24.906 +/- (2.777)	0.538 +/- (0.098)
			Augmented RAVEN	0.277 +/- (0.059)	0.095 +/- (0.017)	24.742 +/- (2.494)	0.539 +/- (0.102)
			In-vivo RAVEN	0.269 +/- (0.050)	0.095 +/- (0.019)	24.864 +/- (2.704)	0.537 +/- (0.104)
			L1 RAVEN	0.360 +/- (0.096)	0.091 +/- (0.018)	25.242 +/- (2.258)	0.549 +/- (0.107)
			VGG-16 RAVEN	0.266 +/- (0.042)	0.086 +/- (0.015)	26.088 +/- (1.812)	0.543 +/- (0.103)
			SuperFormer	0.503 +/- (0.117)	0.094 +/- (0.023)	25.516 +/- (3.443)	0.521 +/- (0.119)
			Trilinear	0.512 +/- (0.117)	0.089 +/- (0.023)	25.670 +/- (2.490)	0.528 +/- (0.121)
	Deg. B	2×2×2	ArSSR-RDN	0.218 +/- (0.058)	0.059 +/- (0.023)	29.201 +/- (4.774)	0.758 +/- (0.104)
			ArSSR-ResCNN	0.278 +/- (0.101)	0.061 +/- (0.022)	28.324 +/- (3.768)	0.760 +/- (0.086)
			Lanczos	0.164 +/- (0.098)	0.036 +/- (0.019)	35.024 +/- (7.393)	0.886 +/- (0.082)
			NLMUPSAMPLE	0.205 +/- (0.113)	0.041 +/- (0.021)	33.616 +/- (6.801)	0.859 +/- (0.093)
			RAVEN	0.096 +/- (0.060)	0.041 +/- (0.020)	32.047 +/- (5.094)	0.889 +/- (0.076)
			4× RAVEN	0.128 +/- (0.069)	0.044 +/- (0.020)	31.984 +/- (4.828)	0.866 +/- (0.064)
			Augmented RAVEN	0.115 +/- (0.068)	0.041 +/- (0.018)	32.287 +/- (4.593)	0.877 +/- (0.071)
			In-vivo RAVEN	0.105 +/- (0.057)	0.044 +/- (0.017)	31.065 +/- (3.955)	0.877 +/- (0.065)
			L1 RAVEN	0.124 +/- (0.089)	0.036 +/- (0.017)	33.642 +/- (5.086)	0.893 +/- (0.079)
			VGG-16 RAVEN	0.096 +/- (0.049)	0.042 +/- (0.012)	31.793 +/- (3.055)	0.886 +/- (0.062)
			SuperFormer	0.223 +/- (0.051)	0.084 +/- (0.029)	25.123 +/- (4.353)	0.799 +/- (0.063)
			Trilinear	0.186 +/- (0.075)	0.044 +/- (0.021)	33.009 +/- (6.440)	0.845 +/- (0.092)
		3×3×3	ArSSR-RDN	0.326 +/- (0.097)	0.078 +/- (0.034)	27.042 +/- (5.098)	0.638 +/- (0.137)
			ArSSR-ResCNN	0.395 +/- (0.143)	0.076 +/- (0.033)	27.621 +/- (4.899)	0.634 +/- (0.129)

			Lanczos	0.393 +/- (0.150)	0.058 +/- (0.023)	30.121 +/- (5.082)	0.735 +/- (0.129)
			NLMUPSAMPLE	0.371 +/- (0.143)	0.058 +/- (0.023)	30.008 +/- (4.883)	0.730 +/- (0.128)
			RAVEN	0.232 +/- (0.116)	0.061 +/- (0.024)	28.424 +/- (4.680)	0.784 +/- (0.118)
			4× RAVEN	0.207 +/- (0.097)	0.061 +/- (0.022)	28.324 +/- (4.107)	0.785 +/- (0.109)
			Augmented RAVEN	0.220 +/- (0.110)	0.062 +/- (0.022)	27.893 +/- (3.972)	0.785 +/- (0.113)
			In-vivo RAVEN	0.216 +/- (0.104)	0.059 +/- (0.021)	28.658 +/- (4.187)	0.787 +/- (0.109)
			L1 RAVEN	0.311 +/- (0.134)	0.060 +/- (0.023)	28.690 +/- (4.096)	0.759 +/- (0.123)
			VGG-16 RAVEN	0.198 +/- (0.097)	0.053 +/- (0.014)	29.802 +/- (2.731)	0.804 +/- (0.104)
			SuperFormer	0.455 +/- (0.096)	0.096 +/- (0.024)	23.888 +/- (3.339)	0.640 +/- (0.105)
			Trilinear	0.434 +/- (0.128)	0.065 +/- (0.023)	28.696 +/- (4.197)	0.681 +/- (0.134)
		4×4×4	ArSSR-RDN	0.467 +/- (0.105)	0.080 +/- (0.024)	26.515 +/- (2.941)	0.582 +/- (0.139)
			ArSSR-ResCNN	0.489 +/- (0.108)	0.082 +/- (0.030)	26.612 +/- (4.122)	0.577 +/- (0.144)
			Lanczos	0.551 +/- (0.126)	0.090 +/- (0.023)	25.552 +/- (2.398)	0.527 +/- (0.122)
			NLMUPSAMPLE	0.550 +/- (0.123)	0.090 +/- (0.023)	25.445 +/- (2.351)	0.523 +/- (0.123)
			RAVEN	0.408 +/- (0.086)	0.100 +/- (0.021)	24.079 +/- (2.550)	0.521 +/- (0.115)
			4× RAVEN	0.315 +/- (0.064)	0.100 +/- (0.023)	24.126 +/- (2.948)	0.532 +/- (0.104)
			Augmented RAVEN	0.379 +/- (0.076)	0.102 +/- (0.021)	23.895 +/- (2.584)	0.520 +/- (0.111)
			In-vivo RAVEN	0.361 +/- (0.063)	0.100 +/- (0.020)	24.135 +/- (2.588)	0.519 +/- (0.113)
			L1 RAVEN	0.490 +/- (0.104)	0.098 +/- (0.020)	24.399 +/- (2.208)	0.520 +/- (0.112)
			VGG-16 RAVEN	0.365 +/- (0.062)	0.091 +/- (0.016)	25.347 +/- (1.826)	0.525 +/- (0.112)
			SuperFormer	0.577 +/- (0.109)	0.097 +/- (0.024)	25.095 +/- (3.495)	0.496 +/- (0.125)
			Trilinear	0.588 +/- (0.111)	0.094 +/- (0.023)	24.974 +/- (2.252)	0.502 +/- (0.128)
	Deg. C	2×2×2	ArSSR-RDN	0.193 +/- (0.053)	0.056 +/- (0.019)	29.598 +/- (4.209)	0.761 +/- (0.104)
			ArSSR-ResCNN	0.236 +/- (0.084)	0.059 +/- (0.018)	28.206 +/- (3.259)	0.778 +/- (0.077)
			Lanczos	0.119 +/- (0.094)	0.027 +/- (0.015)	37.582 +/- (8.531)	0.909 +/- (0.070)
			NLMUPSAMPLE	0.143 +/- (0.102)	0.032 +/- (0.017)	36.441 +/- (7.975)	0.896 +/- (0.080)
			RAVEN	0.097 +/- (0.055)	0.039 +/- (0.018)	32.484 +/- (4.675)	0.886 +/- (0.065)
			4× RAVEN	0.129 +/- (0.067)	0.043 +/- (0.017)	31.802 +/- (4.115)	0.866 +/- (0.055)
			Augmented RAVEN	0.118 +/- (0.062)	0.039 +/- (0.015)	32.513 +/- (4.006)	0.872 +/- (0.058)
			In-vivo RAVEN	0.106 +/- (0.050)	0.044 +/- (0.014)	31.105 +/- (3.643)	0.869 +/- (0.055)
			L1 RAVEN	0.118 +/- (0.074)	0.038 +/- (0.012)	32.416 +/- (3.529)	0.891 +/- (0.064)
			VGG-16 RAVEN	0.110 +/- (0.049)	0.045 +/- (0.012)	30.999 +/- (2.764)	0.868 +/- (0.053)
			SuperFormer	0.150 +/- (0.035)	0.079 +/- (0.032)	25.850 +/- (4.687)	0.830 +/- (0.054)
			Trilinear	0.113 +/- (0.050)	0.037 +/- (0.018)	34.827 +/- (7.238)	0.874 +/- (0.083)
		3×3×3	ArSSR-RDN	0.224 +/- (0.094)	0.072 +/- (0.031)	28.299 +/- (5.535)	0.646 +/- (0.139)
			ArSSR-ResCNN	0.272 +/- (0.136)	0.070 +/- (0.031)	28.553 +/- (5.577)	0.666 +/- (0.115)
			Lanczos	0.273 +/- (0.148)	0.044 +/- (0.019)	32.696 +/- (6.517)	0.802 +/- (0.112)
			NLMUPSAMPLE	0.222 +/- (0.129)	0.043 +/- (0.019)	33.046 +/- (6.243)	0.805 +/- (0.110)
			RAVEN	0.176 +/- (0.098)	0.052 +/- (0.020)	30.031 +/- (4.628)	0.802 +/- (0.104)
			4× RAVEN	0.179 +/- (0.086)	0.052 +/- (0.018)	29.965 +/- (4.020)	0.790 +/- (0.096)
			Augmented RAVEN	0.181 +/- (0.090)	0.051 +/- (0.017)	30.008 +/- (4.046)	0.792 +/- (0.098)
			In-vivo RAVEN	0.173 +/- (0.079)	0.053 +/- (0.017)	29.589 +/- (3.840)	0.791 +/- (0.096)
			L1 RAVEN	0.205 +/- (0.113)	0.048 +/- (0.017)	30.833 +/- (4.351)	0.807 +/- (0.107)
			VGG-16 RAVEN	0.174 +/- (0.076)	0.050 +/- (0.010)	30.232 +/- (2.084)	0.789 +/- (0.094)
			SuperFormer	0.309 +/- (0.076)	0.092 +/- (0.029)	24.327 +/- (3.842)	0.714 +/- (0.090)
			Trilinear	0.277 +/- (0.111)	0.053 +/- (0.021)	31.179 +/- (5.658)	0.756 +/- (0.122)
		4×4×4	ArSSR-RDN	0.350 +/- (0.132)	0.094 +/- (0.035)	26.578 +/- (4.760)	0.511 +/- (0.166)
			ArSSR-ResCNN	0.359 +/- (0.133)	0.096 +/- (0.035)	26.335 +/- (4.936)	0.508 +/- (0.169)
			Lanczos	0.404 +/- (0.165)	0.060 +/- (0.025)	29.920 +/- (5.213)	0.689 +/- (0.142)
			NLMUPSAMPLE	0.394 +/- (0.162)	0.060 +/- (0.025)	29.972 +/- (5.225)	0.691 +/- (0.143)
			RAVEN	0.249 +/- (0.099)	0.080 +/- (0.028)	25.855 +/- (3.884)	0.668 +/- (0.132)
			4× RAVEN	0.222 +/- (0.077)	0.078 +/- (0.024)	26.110 +/- (3.575)	0.657 +/- (0.129)
			Augmented RAVEN	0.234 +/- (0.064)	0.077 +/- (0.027)	26.426 +/- (3.430)	0.647 +/- (0.131)
			In-vivo RAVEN	0.235 +/- (0.067)	0.080 +/- (0.026)	25.863 +/- (3.588)	0.650 +/- (0.130)
			L1 RAVEN	0.287 +/- (0.115)	0.081 +/- (0.035)	25.882 +/- (4.387)	0.671 +/- (0.139)

			VGG-16 RAVEN	0.265 +/- (0.053)	0.068 +/- (0.017)	27.974 +/- (2.323)	0.630 +/- (0.129)
			SuperFormer	0.430 +/- (0.132)	0.110 +/- (0.021)	22.403 +/- (2.396)	0.617 +/- (0.126)
			Trilinear	0.423 +/- (0.135)	0.067 +/- (0.024)	28.613 +/- (4.427)	0.648 +/- (0.143)
0.60-0.90 mm	Deg. A	2×2×2	ArSSR-RDN	0.186 +/- (0.046)	0.050 +/- (0.022)	30.506 +/- (3.638)	0.823 +/- (0.057)
			ArSSR-ResCNN	0.236 +/- (0.045)	0.057 +/- (0.025)	29.126 +/- (3.721)	0.782 +/- (0.055)
			Lanczos	0.159 +/- (0.038)	0.034 +/- (0.019)	35.816 +/- (4.164)	0.878 +/- (0.052)
			NLMUPSAMPLE	0.172 +/- (0.039)	0.036 +/- (0.019)	34.583 +/- (3.649)	0.875 +/- (0.043)
			RAVEN	0.052 +/- (0.022)	0.031 +/- (0.018)	35.887 +/- (3.672)	0.902 +/- (0.046)
			4× RAVEN	0.073 +/- (0.027)	0.037 +/- (0.015)	34.012 +/- (2.908)	0.880 +/- (0.043)
			Augmented RAVEN	0.065 +/- (0.030)	0.037 +/- (0.020)	34.180 +/- (3.454)	0.883 +/- (0.046)
			In-vivo RAVEN	0.058 +/- (0.022)	0.037 +/- (0.018)	34.056 +/- (3.474)	0.886 +/- (0.042)
			L1 RAVEN	0.070 +/- (0.032)	0.032 +/- (0.019)	35.745 +/- (4.038)	0.901 +/- (0.043)
			VGG-16 RAVEN	0.056 +/- (0.023)	0.040 +/- (0.015)	33.142 +/- (3.415)	0.886 +/- (0.043)
			SuperFormer	0.149 +/- (0.028)	0.061 +/- (0.034)	29.164 +/- (4.036)	0.825 +/- (0.053)
			Trilinear	0.132 +/- (0.024)	0.040 +/- (0.019)	34.055 +/- (3.587)	0.852 +/- (0.041)
		3×3×3	ArSSR-RDN	0.290 +/- (0.053)	0.075 +/- (0.028)	26.977 +/- (3.084)	0.663 +/- (0.061)
			ArSSR-ResCNN	0.348 +/- (0.043)	0.083 +/- (0.030)	26.008 +/- (3.287)	0.624 +/- (0.058)
			Lanczos	0.361 +/- (0.050)	0.058 +/- (0.027)	30.365 +/- (3.473)	0.737 +/- (0.055)
			NLMUPSAMPLE	0.302 +/- (0.047)	0.056 +/- (0.025)	30.657 +/- (3.371)	0.751 +/- (0.049)
			RAVEN	0.143 +/- (0.041)	0.046 +/- (0.026)	32.138 +/- (3.840)	0.834 +/- (0.052)
			4× RAVEN	0.141 +/- (0.041)	0.049 +/- (0.025)	31.090 +/- (3.182)	0.818 +/- (0.049)
			Augmented RAVEN	0.144 +/- (0.050)	0.048 +/- (0.028)	31.437 +/- (3.731)	0.827 +/- (0.053)
			In-vivo RAVEN	0.143 +/- (0.049)	0.049 +/- (0.027)	31.458 +/- (3.764)	0.831 +/- (0.052)
			L1 RAVEN	0.205 +/- (0.052)	0.048 +/- (0.027)	31.765 +/- (3.841)	0.812 +/- (0.050)
			VGG-16 RAVEN	0.133 +/- (0.042)	0.049 +/- (0.024)	31.265 +/- (3.708)	0.842 +/- (0.050)
			SuperFormer	0.352 +/- (0.035)	0.083 +/- (0.028)	26.206 +/- (3.185)	0.663 +/- (0.048)
			Trilinear	0.344 +/- (0.040)	0.069 +/- (0.027)	28.434 +/- (3.094)	0.682 +/- (0.050)
		4×4×4	ArSSR-RDN	0.363 +/- (0.064)	0.090 +/- (0.032)	26.189 +/- (2.990)	0.561 +/- (0.078)
			ArSSR-ResCNN	0.383 +/- (0.060)	0.088 +/- (0.033)	26.294 +/- (3.088)	0.569 +/- (0.078)
			Lanczos	0.515 +/- (0.040)	0.104 +/- (0.031)	24.973 +/- (2.578)	0.477 +/- (0.066)
			NLMUPSAMPLE	0.506 +/- (0.040)	0.105 +/- (0.030)	24.917 +/- (2.498)	0.475 +/- (0.064)
			RAVEN	0.268 +/- (0.032)	0.105 +/- (0.032)	24.874 +/- (2.636)	0.471 +/- (0.068)
			4× RAVEN	0.209 +/- (0.033)	0.104 +/- (0.032)	25.217 +/- (2.608)	0.480 +/- (0.074)
			Augmented RAVEN	0.249 +/- (0.037)	0.105 +/- (0.033)	24.876 +/- (2.658)	0.469 +/- (0.066)
			In-vivo RAVEN	0.242 +/- (0.037)	0.106 +/- (0.033)	25.004 +/- (2.683)	0.471 +/- (0.069)
			L1 RAVEN	0.336 +/- (0.039)	0.104 +/- (0.032)	25.095 +/- (2.595)	0.478 +/- (0.067)
			VGG-16 RAVEN	0.258 +/- (0.028)	0.104 +/- (0.031)	25.109 +/- (2.780)	0.474 +/- (0.065)
			SuperFormer	0.508 +/- (0.034)	0.113 +/- (0.031)	23.836 +/- (2.374)	0.446 +/- (0.058)
			Trilinear	0.511 +/- (0.037)	0.111 +/- (0.030)	24.101 +/- (2.406)	0.447 +/- (0.059)
	Deg. B	2×2×2	ArSSR-RDN	0.229 +/- (0.052)	0.057 +/- (0.026)	29.238 +/- (3.833)	0.803 +/- (0.057)
			ArSSR-ResCNN	0.279 +/- (0.049)	0.063 +/- (0.028)	28.263 +/- (3.775)	0.757 +/- (0.055)
			Lanczos	0.206 +/- (0.045)	0.040 +/- (0.022)	33.834 +/- (3.810)	0.861 +/- (0.048)
			NLMUPSAMPLE	0.238 +/- (0.046)	0.045 +/- (0.023)	32.550 +/- (3.647)	0.835 +/- (0.045)
			RAVEN	0.059 +/- (0.026)	0.032 +/- (0.021)	35.455 +/- (3.936)	0.909 +/- (0.044)
			4× RAVEN	0.079 +/- (0.031)	0.038 +/- (0.018)	33.551 +/- (3.134)	0.883 +/- (0.042)
			Augmented RAVEN	0.071 +/- (0.038)	0.037 +/- (0.023)	33.801 +/- (3.758)	0.891 +/- (0.046)
			In-vivo RAVEN	0.063 +/- (0.029)	0.037 +/- (0.021)	33.905 +/- (3.750)	0.897 +/- (0.042)
			L1 RAVEN	0.086 +/- (0.041)	0.033 +/- (0.023)	35.289 +/- (4.358)	0.906 +/- (0.044)
			VGG-16 RAVEN	0.061 +/- (0.028)	0.040 +/- (0.018)	33.113 +/- (3.689)	0.898 +/- (0.042)
			SuperFormer	0.208 +/- (0.033)	0.066 +/- (0.029)	28.369 +/- (3.782)	0.790 +/- (0.048)
			Trilinear	0.191 +/- (0.035)	0.049 +/- (0.024)	31.916 +/- (3.592)	0.814 +/- (0.045)
		3×3×3	ArSSR-RDN	0.358 +/- (0.046)	0.079 +/- (0.031)	26.806 +/- (3.060)	0.627 +/- (0.061)
			ArSSR-ResCNN	0.421 +/- (0.040)	0.087 +/- (0.031)	26.034 +/- (3.036)	0.585 +/- (0.058)
			Lanczos	0.432 +/- (0.048)	0.069 +/- (0.028)	28.366 +/- (3.107)	0.681 +/- (0.051)
			NLMUPSAMPLE	0.403 +/- (0.046)	0.069 +/- (0.027)	28.263 +/- (3.066)	0.678 +/- (0.050)

			RAVEN	0.206 +/- (0.048)	0.057 +/- (0.029)	29.959 +/- (3.622)	0.775 +/- (0.056)
			4× RAVEN	0.172 +/- (0.044)	0.057 +/- (0.027)	29.759 +/- (3.163)	0.783 +/- (0.051)
			Augmented RAVEN	0.193 +/- (0.053)	0.057 +/- (0.030)	29.808 +/- (3.574)	0.780 +/- (0.054)
			In-vivo RAVEN	0.186 +/- (0.053)	0.057 +/- (0.029)	30.032 +/- (3.584)	0.789 +/- (0.056)
			L1 RAVEN	0.294 +/- (0.053)	0.061 +/- (0.029)	29.396 +/- (3.410)	0.739 +/- (0.052)
			VGG-16 RAVEN	0.180 +/- (0.046)	0.057 +/- (0.027)	29.775 +/- (3.638)	0.798 +/- (0.050)
			SuperFormer	0.441 +/- (0.035)	0.096 +/- (0.030)	24.763 +/- (3.048)	0.599 +/- (0.050)
			Trilinear	0.438 +/- (0.041)	0.080 +/- (0.028)	26.768 +/- (2.812)	0.620 +/- (0.051)
		4×4×4	ArSSR-RDN	0.480 +/- (0.037)	0.103 +/- (0.031)	24.551 +/- (2.637)	0.494 +/- (0.061)
			ArSSR-ResCNN	0.506 +/- (0.033)	0.103 +/- (0.034)	24.426 +/- (2.886)	0.495 +/- (0.065)
			Lanczos	0.574 +/- (0.037)	0.112 +/- (0.030)	23.959 +/- (2.372)	0.445 +/- (0.058)
			NLMUPSAMPLE	0.573 +/- (0.037)	0.114 +/- (0.031)	23.785 +/- (2.381)	0.437 +/- (0.059)
			RAVEN	0.384 +/- (0.032)	0.114 +/- (0.032)	23.784 +/- (2.556)	0.440 +/- (0.060)
			4× RAVEN	0.271 +/- (0.036)	0.109 +/- (0.033)	24.311 +/- (2.584)	0.464 +/- (0.065)
			Augmented RAVEN	0.345 +/- (0.041)	0.113 +/- (0.034)	23.858 +/- (2.584)	0.438 +/- (0.058)
			In-vivo RAVEN	0.327 +/- (0.038)	0.112 +/- (0.033)	24.099 +/- (2.571)	0.443 +/- (0.060)
			L1 RAVEN	0.480 +/- (0.035)	0.113 +/- (0.032)	23.891 +/- (2.477)	0.441 +/- (0.058)
			VGG-16 RAVEN	0.341 +/- (0.031)	0.112 +/- (0.031)	23.965 +/- (2.622)	0.445 +/- (0.056)
			SuperFormer	0.585 +/- (0.031)	0.122 +/- (0.030)	22.919 +/- (2.203)	0.409 +/- (0.052)
			Trilinear	0.590 +/- (0.033)	0.120 +/- (0.030)	23.113 +/- (2.282)	0.410 +/- (0.053)
	Deg. C	2×2×2	ArSSR-RDN	0.187 +/- (0.052)	0.049 +/- (0.021)	30.819 +/- (3.399)	0.821 +/- (0.056)
			ArSSR-ResCNN	0.229 +/- (0.051)	0.055 +/- (0.024)	29.570 +/- (3.582)	0.791 +/- (0.054)
			Lanczos	0.156 +/- (0.045)	0.033 +/- (0.020)	35.084 +/- (3.976)	0.884 +/- (0.051)
			NLMUPSAMPLE	0.177 +/- (0.049)	0.035 +/- (0.019)	35.057 +/- (3.686)	0.878 +/- (0.046)
			RAVEN	0.060 +/- (0.030)	0.031 +/- (0.018)	35.367 +/- (3.713)	0.901 +/- (0.044)
			4× RAVEN	0.079 +/- (0.034)	0.036 +/- (0.016)	34.082 +/- (3.150)	0.884 +/- (0.042)
			Augmented RAVEN	0.074 +/- (0.038)	0.035 +/- (0.018)	34.340 +/- (3.294)	0.884 +/- (0.043)
			In-vivo RAVEN	0.064 +/- (0.030)	0.037 +/- (0.017)	33.683 +/- (3.273)	0.884 +/- (0.041)
			L1 RAVEN	0.078 +/- (0.039)	0.032 +/- (0.019)	35.505 +/- (3.977)	0.902 +/- (0.043)
			VGG-16 RAVEN	0.070 +/- (0.033)	0.041 +/- (0.014)	32.287 +/- (3.026)	0.879 +/- (0.042)
			SuperFormer	0.137 +/- (0.031)	0.058 +/- (0.025)	29.313 +/- (3.522)	0.828 +/- (0.046)
			Trilinear	0.123 +/- (0.033)	0.041 +/- (0.021)	33.536 +/- (3.544)	0.843 +/- (0.048)
		3×3×3	ArSSR-RDN	0.280 +/- (0.053)	0.071 +/- (0.025)	27.643 +/- (2.776)	0.655 +/- (0.058)
			ArSSR-ResCNN	0.316 +/- (0.046)	0.076 +/- (0.027)	26.882 +/- (3.038)	0.638 +/- (0.056)
			Lanczos	0.348 +/- (0.057)	0.053 +/- (0.025)	30.891 +/- (3.569)	0.753 +/- (0.058)
			NLMUPSAMPLE	0.281 +/- (0.054)	0.050 +/- (0.022)	31.437 +/- (3.259)	0.761 +/- (0.052)
			RAVEN	0.173 +/- (0.045)	0.047 +/- (0.023)	31.970 +/- (3.368)	0.793 +/- (0.054)
			4× RAVEN	0.165 +/- (0.043)	0.049 +/- (0.022)	31.239 +/- (2.947)	0.787 +/- (0.051)
			Augmented RAVEN	0.175 +/- (0.051)	0.049 +/- (0.023)	31.443 +/- (3.175)	0.782 +/- (0.053)
			In-vivo RAVEN	0.166 +/- (0.048)	0.050 +/- (0.023)	31.333 +/- (3.280)	0.789 +/- (0.052)
			L1 RAVEN	0.211 +/- (0.053)	0.048 +/- (0.024)	31.941 +/- (3.516)	0.792 +/- (0.053)
			VGG-16 RAVEN	0.177 +/- (0.046)	0.054 +/- (0.019)	30.100 +/- (2.809)	0.779 +/- (0.054)
			SuperFormer	0.316 +/- (0.042)	0.076 +/- (0.026)	27.095 +/- (3.009)	0.687 +/- (0.051)
			Trilinear	0.311 +/- (0.046)	0.062 +/- (0.026)	29.476 +/- (3.171)	0.703 +/- (0.053)
		4×4×4	ArSSR-RDN	0.333 +/- (0.074)	0.109 +/- (0.028)	24.320 +/- (2.263)	0.433 +/- (0.049)
			ArSSR-ResCNN	0.347 +/- (0.073)	0.108 +/- (0.030)	24.391 +/- (2.397)	0.439 +/- (0.048)
			Lanczos	0.464 +/- (0.051)	0.068 +/- (0.032)	28.419 +/- (3.509)	0.652 +/- (0.067)
			NLMUPSAMPLE	0.448 +/- (0.051)	0.068 +/- (0.031)	28.511 +/- (3.438)	0.651 +/- (0.064)
			RAVEN	0.230 +/- (0.046)	0.071 +/- (0.039)	28.242 +/- (3.779)	0.645 +/- (0.068)
			4× RAVEN	0.191 +/- (0.065)	0.068 +/- (0.047)	28.739 +/- (3.631)	0.672 +/- (0.074)
			Augmented RAVEN	0.221 +/- (0.044)	0.072 +/- (0.037)	28.072 +/- (3.532)	0.629 +/- (0.064)
			In-vivo RAVEN	0.214 +/- (0.043)	0.072 +/- (0.038)	28.149 +/- (3.587)	0.639 +/- (0.064)
			L1 RAVEN	0.281 +/- (0.055)	0.070 +/- (0.038)	28.487 +/- (3.958)	0.655 +/- (0.067)
			VGG-16 RAVEN	0.277 +/- (0.054)	0.079 +/- (0.030)	26.759 +/- (2.912)	0.601 +/- (0.068)
			SuperFormer	0.438 +/- (0.045)	0.082 +/- (0.026)	26.421 +/- (2.693)	0.590 +/- (0.060)

			Trilinear	0.436 +/- (0.044)	0.079 +/- (0.029)	26.990 +/- (2.986)	0.594 +/- (0.059)
> 0.90 mm	Deg. A	2×2×2	ArSSR-RDN	0.193 +/- (0.063)	0.042 +/- (0.039)	32.534 +/- (2.673)	0.825 +/- (0.069)
			ArSSR-ResCNN	0.225 +/- (0.025)	0.045 +/- (0.007)	31.229 +/- (1.491)	0.783 +/- (0.031)
			Lanczos	0.195 +/- (0.026)	0.031 +/- (0.006)	35.423 +/- (1.603)	0.877 +/- (0.027)
			NLMUPSAMPLE	0.195 +/- (0.034)	0.035 +/- (0.017)	33.904 +/- (1.661)	0.867 +/- (0.036)
			RAVEN	0.052 +/- (0.010)	0.028 +/- (0.005)	35.905 +/- (1.757)	0.895 +/- (0.025)
			4× RAVEN	0.086 +/- (0.017)	0.033 +/- (0.006)	34.170 +/- (1.466)	0.876 +/- (0.025)
			Augmented RAVEN	0.066 +/- (0.014)	0.031 +/- (0.006)	34.934 +/- (1.618)	0.881 +/- (0.026)
			In-vivo RAVEN	0.055 +/- (0.010)	0.032 +/- (0.005)	34.377 +/- (1.398)	0.883 +/- (0.025)
			L1 RAVEN	0.069 +/- (0.014)	0.028 +/- (0.005)	36.052 +/- (1.690)	0.898 +/- (0.024)
			VGG-16 RAVEN	0.056 +/- (0.011)	0.039 +/- (0.005)	32.121 +/- (0.988)	0.879 +/- (0.026)
			SuperFormer	0.143 +/- (0.015)	0.051 +/- (0.015)	29.884 +/- (2.554)	0.828 +/- (0.029)
			Trilinear	0.125 +/- (0.012)	0.037 +/- (0.005)	33.456 +/- (1.343)	0.848 +/- (0.025)
		3×3×3	ArSSR-RDN	0.265 +/- (0.020)	0.067 +/- (0.008)	27.708 +/- (1.140)	0.662 +/- (0.034)
			ArSSR-ResCNN	0.309 +/- (0.022)	0.073 +/- (0.008)	26.974 +/- (1.107)	0.613 +/- (0.035)
			Lanczos	0.338 +/- (0.027)	0.052 +/- (0.006)	30.027 +/- (1.054)	0.747 +/- (0.030)
			NLMUPSAMPLE	0.276 +/- (0.023)	0.052 +/- (0.006)	29.958 +/- (1.076)	0.747 +/- (0.030)
			RAVEN	0.157 +/- (0.023)	0.040 +/- (0.006)	32.324 +/- (1.281)	0.826 +/- (0.026)
			4× RAVEN	0.167 +/- (0.022)	0.045 +/- (0.006)	30.982 +/- (1.191)	0.801 +/- (0.028)
			Augmented RAVEN	0.161 +/- (0.020)	0.043 +/- (0.007)	31.658 +/- (1.357)	0.810 +/- (0.029)
			In-vivo RAVEN	0.151 +/- (0.021)	0.042 +/- (0.006)	31.805 +/- (1.261)	0.822 +/- (0.027)
			L1 RAVEN	0.205 +/- (0.025)	0.042 +/- (0.006)	31.660 +/- (1.208)	0.801 +/- (0.026)
			VGG-16 RAVEN	0.151 +/- (0.023)	0.045 +/- (0.005)	30.939 +/- (0.921)	0.834 +/- (0.027)
			SuperFormer	0.311 +/- (0.020)	0.074 +/- (0.014)	26.457 +/- (1.708)	0.661 +/- (0.038)
			Trilinear	0.307 +/- (0.020)	0.065 +/- (0.006)	27.789 +/- (0.872)	0.673 +/- (0.030)
		4×4×4	ArSSR-RDN	0.329 +/- (0.019)	0.083 +/- (0.007)	26.042 +/- (0.883)	0.541 +/- (0.028)
			ArSSR-ResCNN	0.351 +/- (0.021)	0.082 +/- (0.007)	26.059 +/- (0.911)	0.550 +/- (0.029)
			Lanczos	0.472 +/- (0.019)	0.100 +/- (0.010)	24.488 +/- (1.051)	0.433 +/- (0.032)
			NLMUPSAMPLE	0.465 +/- (0.034)	0.103 +/- (0.024)	24.275 +/- (1.591)	0.424 +/- (0.054)
			RAVEN	0.249 +/- (0.013)	0.100 +/- (0.011)	24.434 +/- (1.092)	0.424 +/- (0.033)
			4× RAVEN	0.210 +/- (0.016)	0.099 +/- (0.011)	24.647 +/- (1.109)	0.433 +/- (0.031)
			Augmented RAVEN	0.240 +/- (0.014)	0.099 +/- (0.011)	24.596 +/- (1.159)	0.421 +/- (0.030)
			In-vivo RAVEN	0.221 +/- (0.015)	0.101 +/- (0.011)	24.528 +/- (1.123)	0.420 +/- (0.030)
			L1 RAVEN	0.310 +/- (0.013)	0.098 +/- (0.011)	24.681 +/- (1.154)	0.431 +/- (0.033)
			VGG-16 RAVEN	0.239 +/- (0.016)	0.099 +/- (0.010)	24.565 +/- (0.993)	0.425 +/- (0.032)
			SuperFormer	0.460 +/- (0.020)	0.106 +/- (0.009)	23.673 +/- (0.867)	0.403 +/- (0.032)
			Trilinear	0.462 +/- (0.021)	0.105 +/- (0.009)	23.828 +/- (0.919)	0.403 +/- (0.033)
	Deg. B	2×2×2	ArSSR-RDN	0.222 +/- (0.025)	0.045 +/- (0.006)	31.095 +/- (1.287)	0.811 +/- (0.032)
			ArSSR-ResCNN	0.256 +/- (0.025)	0.052 +/- (0.007)	29.992 +/- (1.246)	0.758 +/- (0.033)
			Lanczos	0.228 +/- (0.027)	0.036 +/- (0.005)	33.440 +/- (1.404)	0.861 +/- (0.026)
			NLMUPSAMPLE	0.247 +/- (0.075)	0.046 +/- (0.049)	31.776 +/- (2.590)	0.826 +/- (0.075)
			RAVEN	0.063 +/- (0.013)	0.027 +/- (0.005)	36.090 +/- (1.696)	0.905 +/- (0.023)
			4× RAVEN	0.093 +/- (0.018)	0.033 +/- (0.005)	33.907 +/- (1.422)	0.879 +/- (0.024)
			Augmented RAVEN	0.077 +/- (0.017)	0.030 +/- (0.006)	35.009 +/- (1.637)	0.889 +/- (0.024)
			In-vivo RAVEN	0.062 +/- (0.013)	0.030 +/- (0.005)	34.689 +/- (1.355)	0.896 +/- (0.022)
			L1 RAVEN	0.089 +/- (0.017)	0.027 +/- (0.005)	36.008 +/- (1.701)	0.905 +/- (0.022)
			VGG-16 RAVEN	0.066 +/- (0.015)	0.037 +/- (0.004)	32.338 +/- (0.971)	0.896 +/- (0.022)
			SuperFormer	0.190 +/- (0.017)	0.055 +/- (0.014)	29.102 +/- (2.190)	0.796 +/- (0.032)
			Trilinear	0.173 +/- (0.015)	0.045 +/- (0.005)	31.349 +/- (1.155)	0.812 +/- (0.027)
		3×3×3	ArSSR-RDN	0.318 +/- (0.018)	0.076 +/- (0.007)	26.504 +/- (0.831)	0.618 +/- (0.033)
			ArSSR-ResCNN	0.381 +/- (0.020)	0.082 +/- (0.007)	25.787 +/- (0.883)	0.562 +/- (0.033)
			Lanczos	0.385 +/- (0.025)	0.065 +/- (0.006)	27.689 +/- (0.850)	0.675 +/- (0.031)
			NLMUPSAMPLE	0.356 +/- (0.021)	0.067 +/- (0.006)	27.533 +/- (0.851)	0.664 +/- (0.031)
			RAVEN	0.205 +/- (0.021)	0.050 +/- (0.006)	30.017 +/- (1.071)	0.763 +/- (0.029)
			4× RAVEN	0.195 +/- (0.020)	0.052 +/- (0.007)	29.644 +/- (1.154)	0.757 +/- (0.031)

			Augmented RAVEN	0.200 +/- (0.018)	0.051 +/- (0.007)	29.880 +/- (1.196)	0.756 +/- (0.029)
			In-vivo RAVEN	0.187 +/- (0.020)	0.049 +/- (0.007)	30.244 +/- (1.215)	0.770 +/- (0.028)
			L1 RAVEN	0.278 +/- (0.024)	0.056 +/- (0.006)	29.072 +/- (0.955)	0.718 +/- (0.027)
			VGG-16 RAVEN	0.184 +/- (0.023)	0.051 +/- (0.005)	29.734 +/- (0.816)	0.788 +/- (0.029)
			SuperFormer	0.387 +/- (0.021)	0.084 +/- (0.011)	25.225 +/- (1.219)	0.589 +/- (0.036)
			Trilinear	0.387 +/- (0.021)	0.077 +/- (0.006)	26.182 +/- (0.750)	0.600 +/- (0.031)
		4×4×4	ArSSR-RDN	0.453 +/- (0.016)	0.096 +/- (0.006)	24.444 +/- (0.703)	0.464 +/- (0.030)
			ArSSR-ResCNN	0.480 +/- (0.015)	0.097 +/- (0.006)	24.289 +/- (0.657)	0.463 +/- (0.028)
			Lanczos	0.529 +/- (0.022)	0.106 +/- (0.009)	23.683 +/- (0.879)	0.401 +/- (0.033)
			NLMUPSAMPLE	0.539 +/- (0.047)	0.117 +/- (0.046)	23.135 +/- (2.325)	0.380 +/- (0.073)
			RAVEN	0.350 +/- (0.013)	0.105 +/- (0.010)	23.790 +/- (0.979)	0.398 +/- (0.034)
			4× RAVEN	0.272 +/- (0.014)	0.101 +/- (0.010)	24.229 +/- (1.026)	0.419 +/- (0.032)
			Augmented RAVEN	0.324 +/- (0.012)	0.104 +/- (0.010)	23.951 +/- (1.025)	0.395 +/- (0.031)
			In-vivo RAVEN	0.304 +/- (0.012)	0.103 +/- (0.011)	24.068 +/- (1.059)	0.398 +/- (0.032)
			L1 RAVEN	0.451 +/- (0.013)	0.105 +/- (0.009)	23.807 +/- (0.950)	0.396 +/- (0.033)
			VGG-16 RAVEN	0.316 +/- (0.014)	0.105 +/- (0.009)	23.639 +/- (0.855)	0.402 +/- (0.033)
			SuperFormer	0.541 +/- (0.023)	0.115 +/- (0.008)	22.819 +/- (0.704)	0.368 +/- (0.032)
			Trilinear	0.545 +/- (0.023)	0.112 +/- (0.008)	23.079 +/- (0.777)	0.369 +/- (0.033)
	Deg. C	2×2×2	ArSSR-RDN	0.196 +/- (0.028)	0.039 +/- (0.007)	32.729 +/- (1.595)	0.830 +/- (0.031)
			ArSSR-ResCNN	0.223 +/- (0.026)	0.043 +/- (0.007)	31.573 +/- (1.491)	0.794 +/- (0.030)
			Lanczos	0.194 +/- (0.026)	0.030 +/- (0.005)	34.846 +/- (1.491)	0.890 +/- (0.023)
			NLMUPSAMPLE	0.205 +/- (0.027)	0.032 +/- (0.005)	34.372 +/- (1.526)	0.875 +/- (0.026)
			RAVEN	0.067 +/- (0.013)	0.027 +/- (0.005)	35.793 +/- (1.615)	0.901 +/- (0.022)
			4× RAVEN	0.098 +/- (0.019)	0.032 +/- (0.005)	34.277 +/- (1.474)	0.883 +/- (0.023)
			Augmented RAVEN	0.086 +/- (0.018)	0.030 +/- (0.005)	34.930 +/- (1.511)	0.887 +/- (0.023)
			In-vivo RAVEN	0.068 +/- (0.013)	0.031 +/- (0.005)	34.380 +/- (1.465)	0.887 +/- (0.022)
			L1 RAVEN	0.087 +/- (0.017)	0.027 +/- (0.005)	35.909 +/- (1.634)	0.905 +/- (0.021)
			VGG-16 RAVEN	0.079 +/- (0.015)	0.039 +/- (0.004)	31.887 +/- (0.811)	0.883 +/- (0.022)
			SuperFormer	0.136 +/- (0.016)	0.051 +/- (0.014)	29.836 +/- (2.399)	0.832 +/- (0.028)
			Trilinear	0.119 +/- (0.013)	0.037 +/- (0.005)	33.103 +/- (1.338)	0.846 +/- (0.026)
		3×3×3	ArSSR-RDN	0.257 +/- (0.022)	0.064 +/- (0.009)	28.246 +/- (1.348)	0.665 +/- (0.032)
			ArSSR-ResCNN	0.282 +/- (0.023)	0.066 +/- (0.010)	27.924 +/- (1.386)	0.637 +/- (0.034)
			Lanczos	0.347 +/- (0.030)	0.047 +/- (0.005)	30.878 +/- (1.054)	0.776 +/- (0.027)
			NLMUPSAMPLE	0.269 +/- (0.026)	0.046 +/- (0.006)	31.082 +/- (1.149)	0.771 +/- (0.028)
			RAVEN	0.184 +/- (0.024)	0.041 +/- (0.006)	32.254 +/- (1.303)	0.803 +/- (0.027)
			4× RAVEN	0.193 +/- (0.023)	0.044 +/- (0.006)	31.365 +/- (1.235)	0.789 +/- (0.029)
			Augmented RAVEN	0.187 +/- (0.020)	0.043 +/- (0.007)	31.695 +/- (1.380)	0.788 +/- (0.030)
			In-vivo RAVEN	0.172 +/- (0.021)	0.042 +/- (0.006)	32.051 +/- (1.347)	0.801 +/- (0.029)
			L1 RAVEN	0.211 +/- (0.025)	0.041 +/- (0.006)	32.219 +/- (1.372)	0.799 +/- (0.028)
			VGG-16 RAVEN	0.191 +/- (0.022)	0.050 +/- (0.005)	29.849 +/- (0.774)	0.793 +/- (0.030)
			SuperFormer	0.291 +/- (0.020)	0.067 +/- (0.013)	27.391 +/- (1.759)	0.696 +/- (0.033)
			Trilinear	0.290 +/- (0.021)	0.058 +/- (0.005)	28.988 +/- (0.940)	0.708 +/- (0.029)
		4×4×4	ArSSR-RDN	0.299 +/- (0.019)	0.102 +/- (0.014)	24.326 +/- (1.538)	0.418 +/- (0.033)
			ArSSR-ResCNN	0.315 +/- (0.023)	0.101 +/- (0.014)	24.382 +/- (1.562)	0.423 +/- (0.034)
			Lanczos	0.418 +/- (0.025)	0.063 +/- (0.006)	28.075 +/- (0.857)	0.659 +/- (0.029)
			NLMUPSAMPLE	0.398 +/- (0.028)	0.065 +/- (0.013)	27.900 +/- (1.252)	0.642 +/- (0.064)
			RAVEN	0.218 +/- (0.011)	0.068 +/- (0.007)	27.204 +/- (0.891)	0.639 +/- (0.034)
			4× RAVEN	0.198 +/- (0.014)	0.064 +/- (0.007)	27.831 +/- (0.899)	0.647 +/- (0.032)
			Augmented RAVEN	0.207 +/- (0.013)	0.070 +/- (0.007)	26.954 +/- (0.954)	0.621 +/- (0.032)
			In-vivo RAVEN	0.196 +/- (0.012)	0.070 +/- (0.006)	26.859 +/- (0.930)	0.635 +/- (0.031)
			L1 RAVEN	0.243 +/- (0.015)	0.076 +/- (0.010)	25.862 +/- (1.543)	0.646 +/- (0.031)
			VGG-16 RAVEN	0.253 +/- (0.014)	0.076 +/- (0.008)	26.345 +/- (1.002)	0.588 +/- (0.030)
			SuperFormer	0.398 +/- (0.016)	0.082 +/- (0.008)	25.610 +/- (0.918)	0.585 +/- (0.032)
			Trilinear	0.380 +/- (0.022)	0.075 +/- (0.006)	26.448 +/- (0.785)	0.580 +/- (0.031)

Table S5: Performance of the different upsampling methods on ground truth LR-HR image pairs (see section 4.6)

Scale	Modality	Method	LPIPS ↓	MAE ↓	PSNR ↑	SSIM ↑
2×2×2	T1w	ArSSR-RDN	0.174 ± (0.026)	0.036 ± (0.016)	39.169 ± (2.604)	0.684 ± (0.045)
		ArSSR-ResCNN	0.172 ± (0.031)	0.036 ± (0.015)	39.255 ± (2.628)	0.697 ± (0.044)
		Lanczos	0.165 ± (0.031)	0.033 ± (0.014)	45.062 ± (3.027)	0.813 ± (0.030)
		NLMUPSAMPLE	0.165 ± (0.028)	0.033 ± (0.014)	44.400 ± (2.977)	0.805 ± (0.032)
		RAVEN	0.159 ± (0.022)	0.034 ± (0.014)	40.365 ± (1.217)	0.783 ± (0.029)
		RAVEN-4x	0.154 ± (0.024)	0.035 ± (0.016)	37.018 ± (1.198)	0.697 ± (0.044)
		RAVEN_L1	0.172 ± (0.025)	0.036 ± (0.016)	37.588 ± (1.378)	0.710 ± (0.050)
		RAVEN-VGG16	0.153 ± (0.032)	0.046 ± (0.018)	30.880 ± (2.189)	0.492 ± (0.100)
		RAVEN-Augmented	0.150 ± (0.027)	0.034 ± (0.015)	40.186 ± (2.169)	0.732 ± (0.031)
		RAVEN_Invivo	0.170 ± (0.019)	0.039 ± (0.015)	35.514 ± (1.280)	0.646 ± (0.055)
		SuperFormer	0.173 ± (0.034)	0.035 ± (0.016)	39.580 ± (2.562)	0.730 ± (0.044)
		Trilinear	0.179 ± (0.025)	0.034 ± (0.015)	43.600 ± (2.826)	0.799 ± (0.035)
	T2w	ArSSR-RDN	0.153 ± (0.033)	0.026 ± (0.012)	38.910 ± (2.248)	0.778 ± (0.042)
		ArSSR-ResCNN	0.157 ± (0.032)	0.027 ± (0.012)	38.864 ± (2.197)	0.779 ± (0.042)
		Lanczos	0.151 ± (0.036)	0.027 ± (0.011)	37.100 ± (3.488)	0.690 ± (0.081)
		NLMUPSAMPLE	0.133 ± (0.035)	0.021 ± (0.010)	47.514 ± (3.560)	0.885 ± (0.041)
		RAVEN	0.116 ± (0.030)	0.022 ± (0.010)	41.983 ± (4.113)	0.829 ± (0.060)
		RAVEN-4x	0.114 ± (0.029)	0.024 ± (0.010)	40.831 ± (5.062)	0.786 ± (0.092)
		RAVEN_L1	0.131 ± (0.029)	0.024 ± (0.010)	39.762 ± (4.282)	0.762 ± (0.091)
		RAVEN-VGG16	0.129 ± (0.027)	0.037 ± (0.012)	33.861 ± (5.178)	0.621 ± (0.158)
		RAVEN-Augmented	0.116 ± (0.031)	0.022 ± (0.012)	40.070 ± (2.446)	0.824 ± (0.037)
		RAVEN_Invivo	0.130 ± (0.022)	0.028 ± (0.010)	37.257 ± (4.254)	0.693 ± (0.103)
		SuperFormer	0.157 ± (0.035)	0.026 ± (0.012)	39.421 ± (2.290)	0.817 ± (0.042)
		Trilinear	0.162 ± (0.032)	0.023 ± (0.011)	44.978 ± (2.768)	0.865 ± (0.045)
3×3×3	T1w	ArSSR-RDN	0.212 ± (0.032)	0.044 ± (0.018)	38.471 ± (2.913)	0.631 ± (0.062)
		ArSSR-ResCNN	0.208 ± (0.041)	0.043 ± (0.019)	38.650 ± (2.999)	0.656 ± (0.064)
		Lanczos	0.242 ± (0.039)	0.040 ± (0.018)	42.713 ± (3.708)	0.743 ± (0.055)
		NLMUPSAMPLE	0.234 ± (0.034)	0.039 ± (0.018)	43.022 ± (3.156)	0.747 ± (0.046)
		RAVEN	0.206 ± (0.022)	0.039 ± (0.018)	40.062 ± (1.963)	0.746 ± (0.044)
		RAVEN-4x	0.190 ± (0.026)	0.041 ± (0.019)	36.870 ± (1.186)	0.673 ± (0.034)
		RAVEN_L1	0.226 ± (0.024)	0.041 ± (0.020)	37.150 ± (1.387)	0.675 ± (0.041)
		RAVEN-VGG16	0.197 ± (0.031)	0.050 ± (0.021)	30.279 ± (1.583)	0.462 ± (0.058)
		RAVEN-Augmented	0.194 ± (0.027)	0.040 ± (0.018)	39.445 ± (2.535)	0.685 ± (0.041)
		RAVEN_Invivo	0.211 ± (0.017)	0.044 ± (0.019)	35.243 ± (1.505)	0.618 ± (0.040)
		SuperFormer	0.241 ± (0.041)	0.041 ± (0.019)	38.948 ± (2.810)	0.680 ± (0.057)
		Trilinear	0.255 ± (0.041)	0.042 ± (0.018)	41.295 ± (2.653)	0.728 ± (0.055)

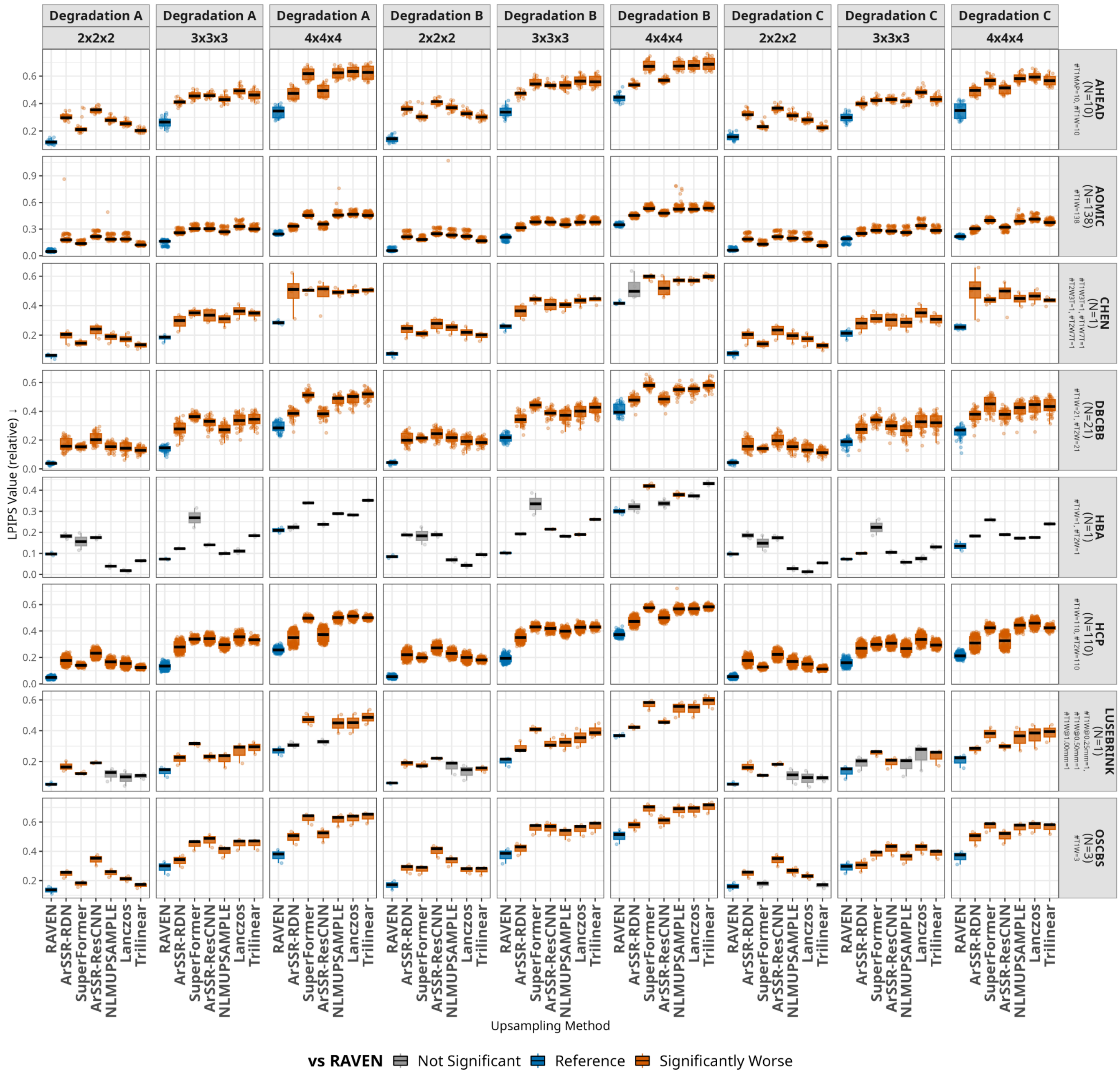

Figure S1: LPIPS (lower is better) benchmark results for super-resolution tasks across datasets and upsampling factors. The color of NLMUPSAMPLE and Trilinear boxplots indicate if no significant differences were found with RAVEN (gray), or if the given method was significantly better (green) or worse (orange) than RAVEN.

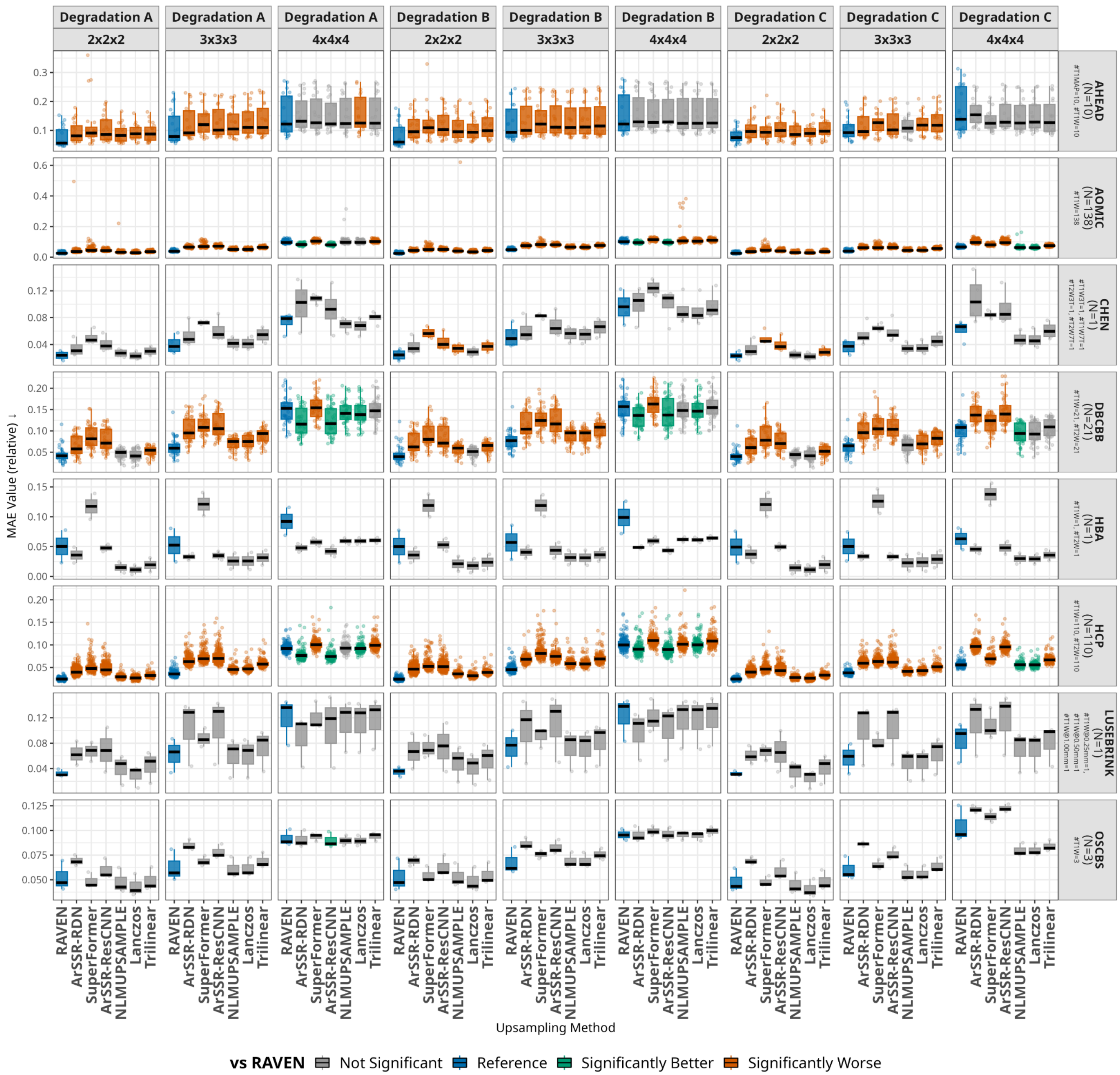

Figure S2: MAE (lower is better) benchmark results for super-resolution tasks across datasets and upsampling factors. The color of NLMUPSAMPLE and Trilinear boxplots indicate if no significant differences were found with RAVEN (gray), or if the given method was significantly better (green) or worse (orange) than RAVEN. The color represents the significance after performing paired t-tests on RAVEN vs each of the methods tested separately.

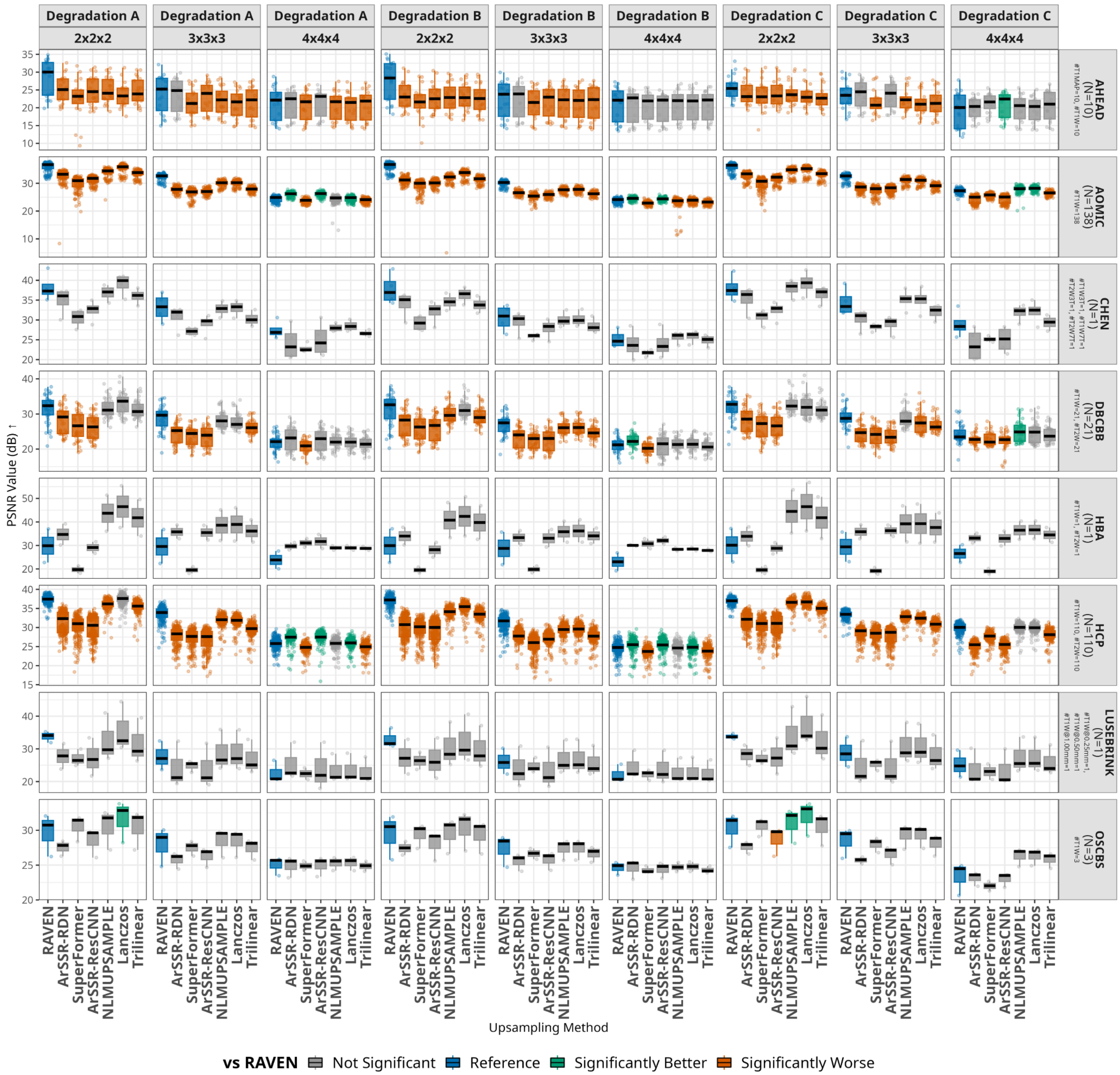

Figure S3: PSNR (higher is better, in dB) benchmark results for super-resolution tasks across datasets and upsampling factors. The color of NLMUPSAMPLE and Trilinear boxplots indicate if no significant differences were found with RAVEN (gray), or if the given method was significantly better (green) or worse (orange) than RAVEN. The color represents the significance after performing paired t-tests on RAVEN vs each of the methods tested separately.

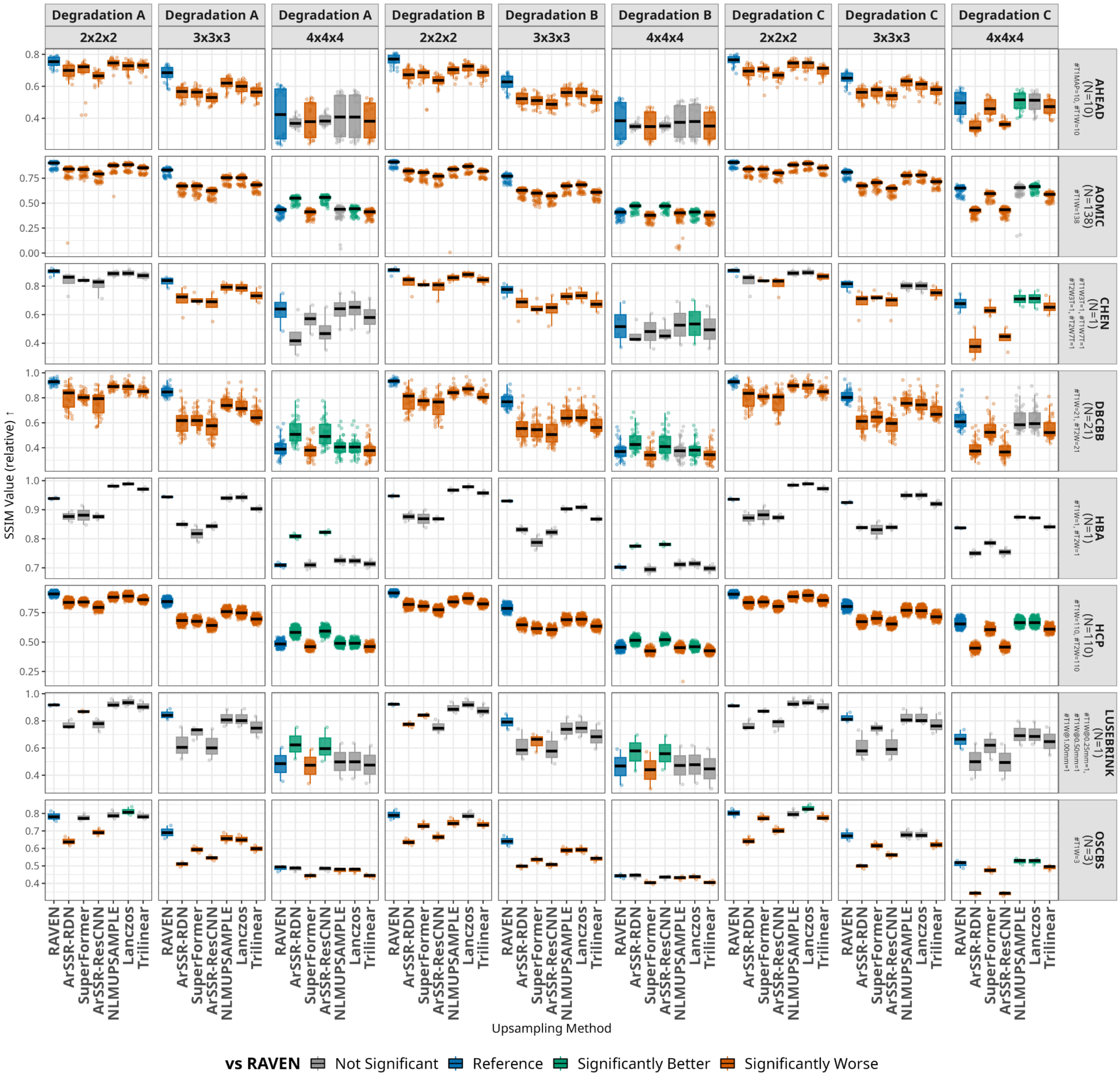

Figure S4: SSIM (higher is better) benchmark results for super-resolution tasks across datasets and upsampling factors. The color of NLMUPSAMPLE and Trilinear boxplots indicate if no significant differences were found with RAVEN (gray), or if the given method was significantly better (green) or worse (orange) than RAVEN. The color represents the significance after performing paired t-tests on RAVEN vs each of the methods tested separately.

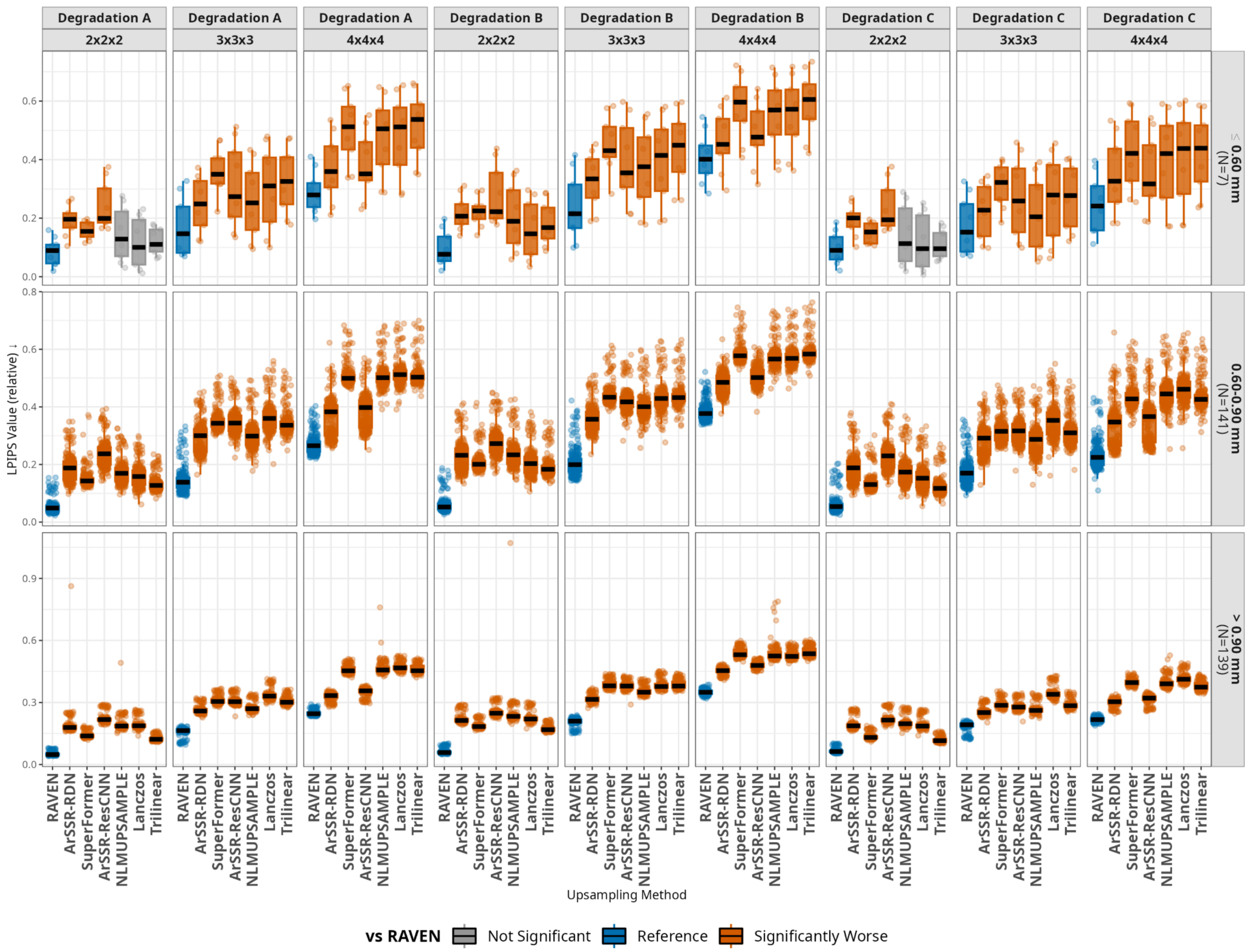

Figure S5: LPIPS (lower is better) benchmark results for super-resolution tasks across different target voxel sizes (row names on the right) and upsampling factors. The color of NLMUPSAMPLE and Trilinear boxplots indicate if no significant differences were found with RAVEN (gray), or if the given method was significantly better (green) or worse (orange) than RAVEN. The color represents the significance after performing paired t-tests on RAVEN vs each of the methods tested separately.

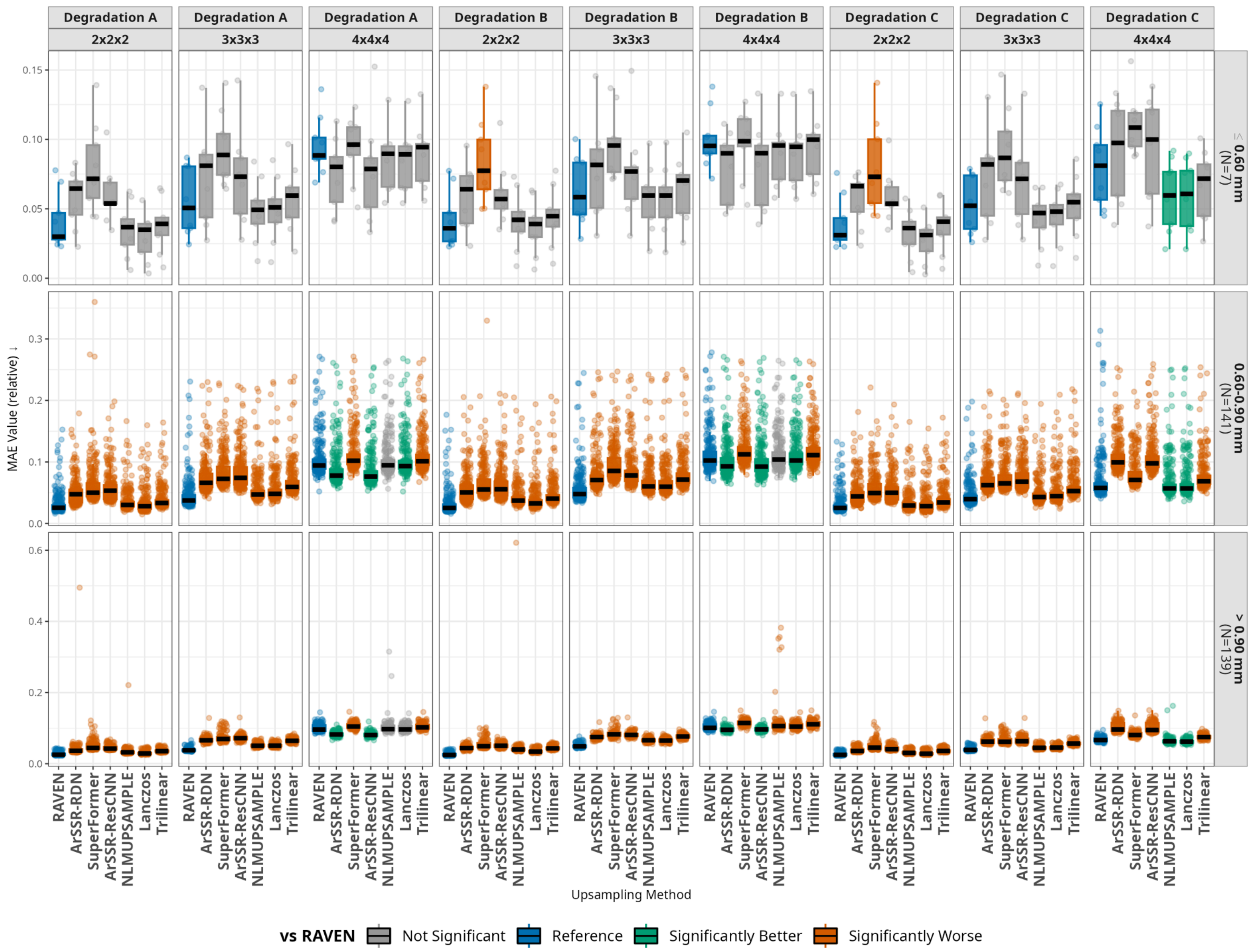

Figure S6: MAE (lower is better) benchmark results for super-resolution tasks across different target voxel sizes (row names on the right) and upsampling factors. The color of NLMUPSAMPLE and Trilinear boxplots indicate if no significant differences were found with RAVEN (gray), or if the given method was significantly better (green) or worse (orange) than RAVEN. The color represents the significance after performing paired t-tests on RAVEN vs each of the methods tested separately.

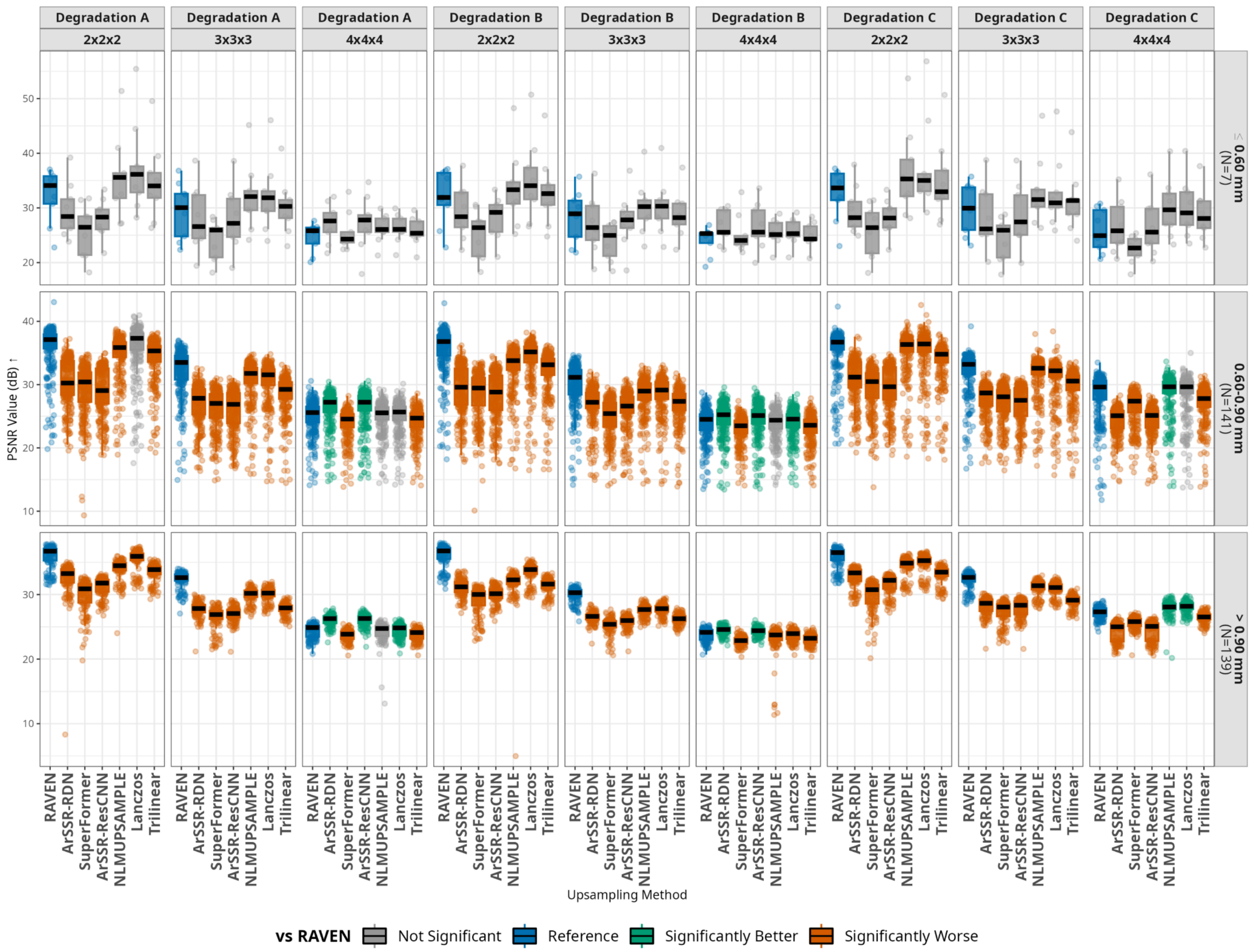

Figure S7: PSNR (higher is better, in dB) benchmark results for super-resolution tasks across different target voxel sizes (row names on the right) and upsampling factors. The color of NLMUPSAMPLE and Trilinear boxplots indicate if no significant differences were found with RAVEN (gray), or if the given method was significantly better (green) or worse (orange) than RAVEN. The color represents the significance after performing paired t-tests on RAVEN vs each of the methods tested separately.

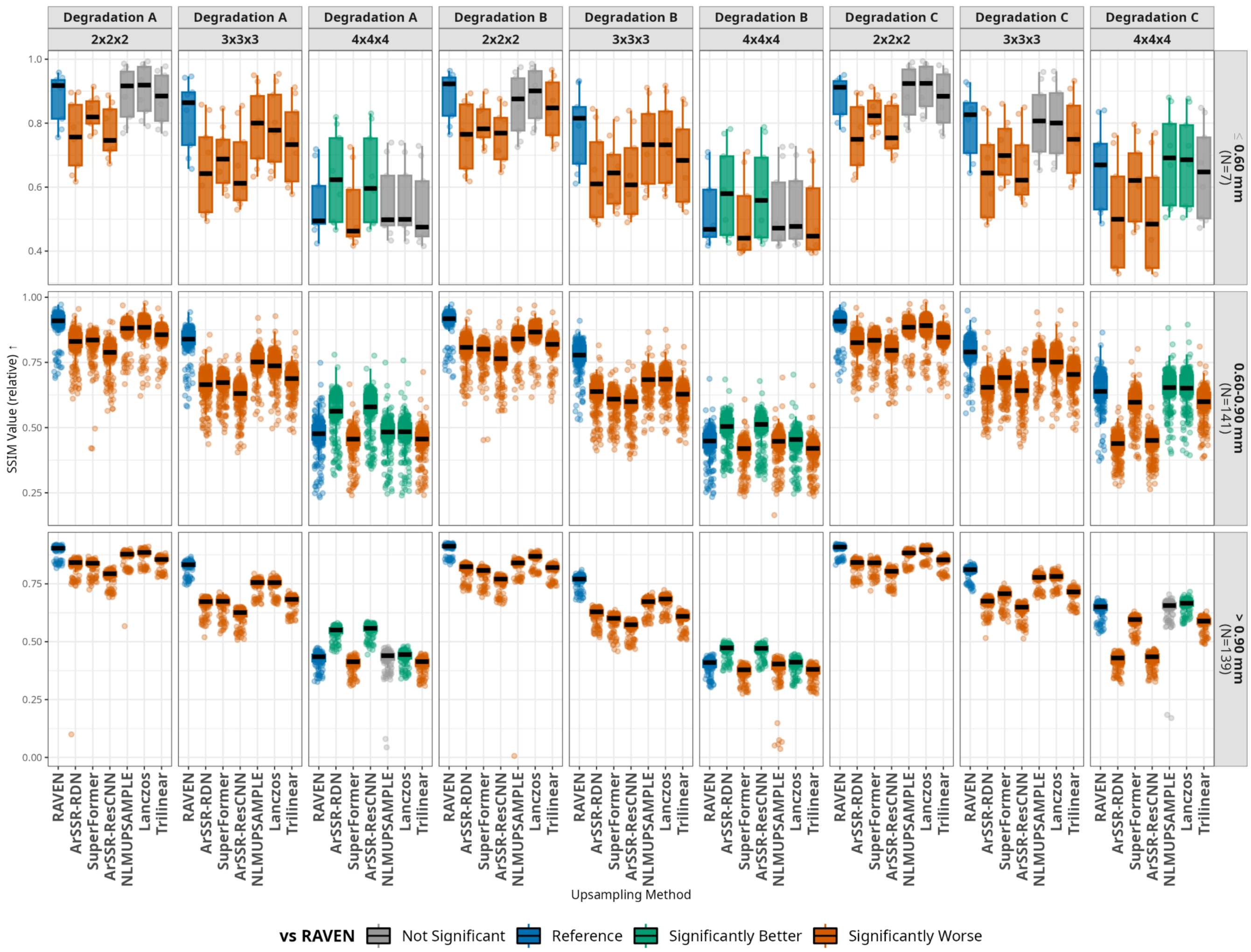

Figure S8: SSIM (higher is better) benchmark results for super-resolution tasks across different target voxel sizes (row names on the right) and upsampling factors. The color of NLMUPSAMPLE and Trilinear boxplots indicate if no significant differences were found with RAVEN (gray), or if the given method was significantly better (green) or worse (orange) than RAVEN. The color represents the significance after performing paired t-tests on RAVEN vs each of the methods tested separately.

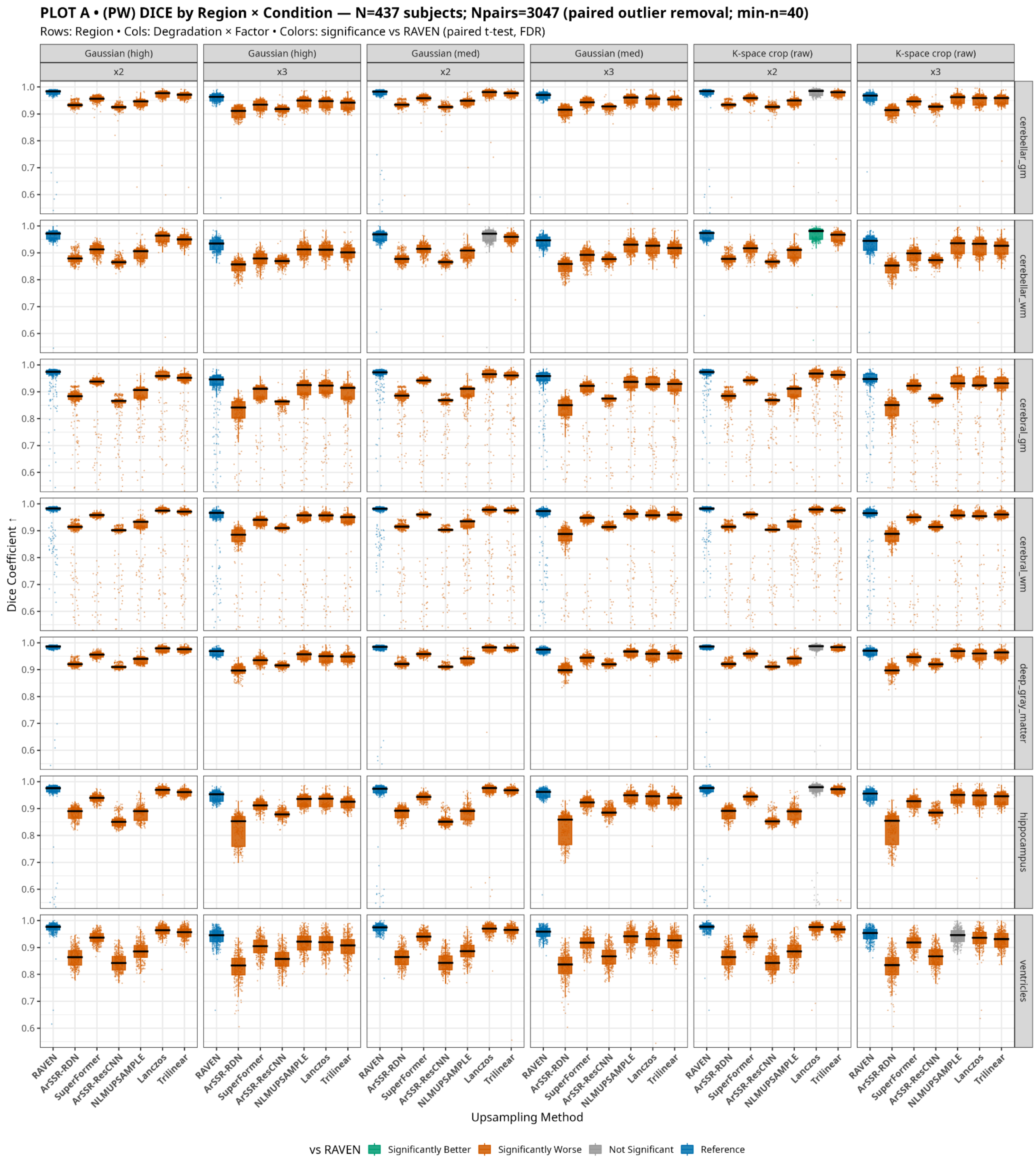

Figure S9: IOU (higher is better) for 7 regions of interest generated by SynthSeg after resampling them to the input's native space. Results separated for different degradation methods and upsampling factors. Blue is the reference method (RAVEN), and orange depicts statistically worse results vs the reference method after FDR correction. The color represents the significance after performing paired t-tests on RAVEN vs each of the methods tested separately.

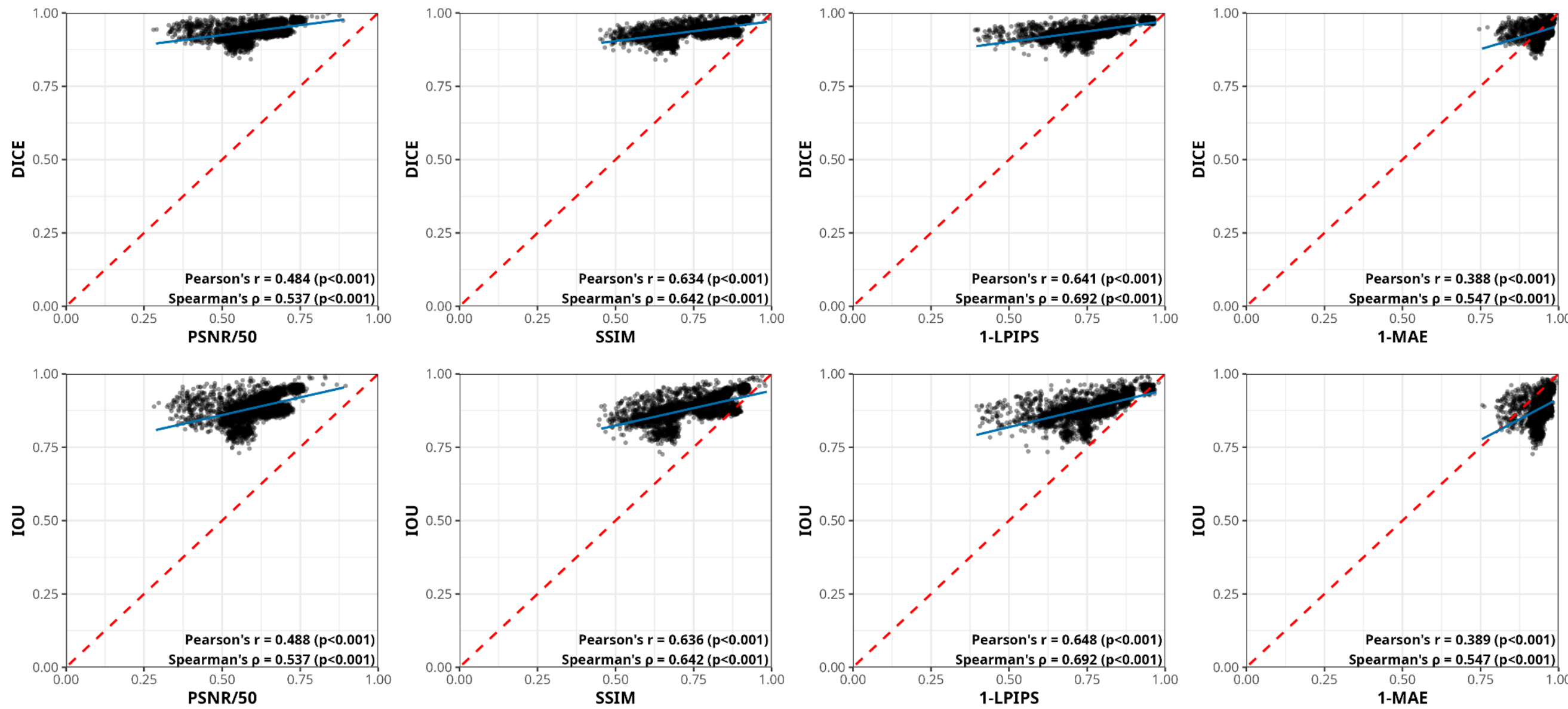

Figure S10. Correlation between image similarity metrics (PSNR, SSIM, LPIPS, and MAE), and segmentation agreement metrics (IOU and DICE) for bilateral hippocampal labels generated by SynthSeg. The correlation between 1-LPIPS was the highest compared to 1-MAE, SSIM, and PSNR/50 (PSNR divided by 50 for visualization purposes).

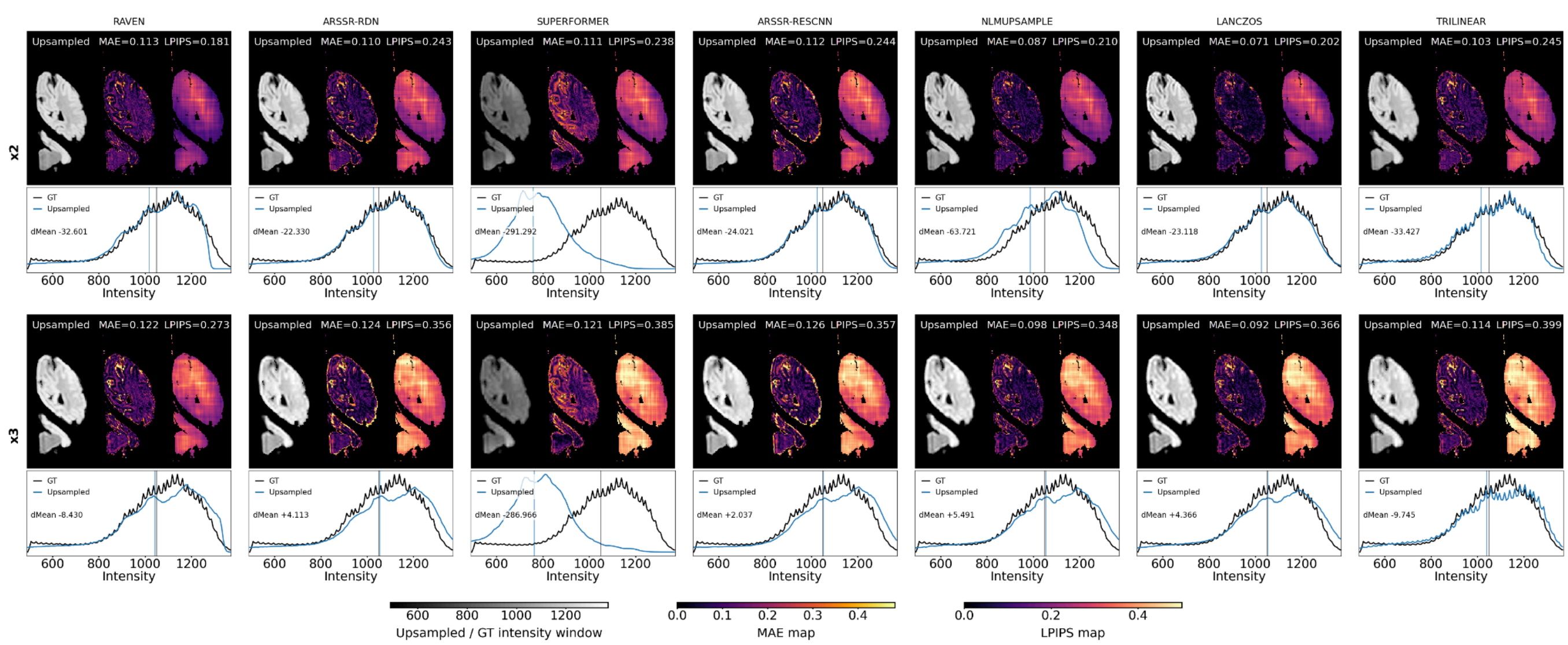

Figure S11. **Top row**: upsampling from native 1.4 mm isotropic post-mortem T1w MRI to 0.7 mm isotropic. **Bottom row**: upsampling from native 2.1 mm isotropic post-mortem T1w MRI to 0.7 mm isotropic. Each column corresponds to a different upsampling method. The error maps show local MAE and LPIPS values relative to the ground-truth (GT) native T1w MRI acquired at 0.7 mm isotropic resolution. The distribution plots below the maps show the voxel-intensity distributions within the brain mask for each method output (blue curves), overlaid with the intensity distribution of the GT reference image (black curves). “dMean” shows the distance between the two curves' mean values. Compared with the other methods, SuperFormer shows a pronounced global intensity shift toward lower intensities, consistent with the visual intensity differences observed in the reconstructed image.
